# Spatially Explicit Trends in the Global Conservation Status of Vertebrates

**DOI:** 10.1371/journal.pone.0113934

**Published:** 2014-11-26

**Authors:** Ana S. L. Rodrigues, Thomas M. Brooks, Stuart H. M. Butchart, Janice Chanson, Neil Cox, Michael Hoffmann, Simon N. Stuart

**Affiliations:** 1 Centre d′Ecologie Fonctionnelle et Evolutive, CNRS UMR5175, 1919 Route de Mende, 34293, Montpellier, France; 2 IUCN, 28 rue Mauverney, CH-1196, Gland, Switzerland; 3 World Agroforestry Center (ICRAF), University of the Philippines Los Baños, Laguna 4031, Philippines; 4 School of Geography and Environmental Studies, University of Tasmania, Hobart TAS 7001, Australia; 5 BirdLife International, Wellbrook Court, Girton Road, Cambridge, CB3 0NA, United Kingdom; 6 Conservation International, 2011 Crystal Drive Ste 500, Arlington, VA, 22202, United States of America; 7 United Nations Environment Programme World Conservation Monitoring Centre, 219 Huntingdon Road, Cambridge, CB3 0DL, United Kingdom; 8 Department of Biology and Biochemistry, University of Bath, Bath, BA2 7AY, United Kingdom; 9 Al Ain Zoo, P.O. Box 45553, Abu Dhabi, United Arab Emirates; Institute of Agronomy, University of Lisbon, Portugal

## Abstract

The world's governments have committed to preventing the extinction of threatened species and improving their conservation status by 2020. However, biodiversity is not evenly distributed across space, and neither are the drivers of its decline, and so different regions face very different challenges. Here, we quantify the contribution of regions and countries towards recent global trends in vertebrate conservation status (as measured by the Red List Index), to guide action towards the 2020 target. We found that>50% of the global deterioration in the conservation status of birds, mammals and amphibians is concentrated in <1% of the surface area, 39/1098 ecoregions (4%) and eight/195 countries (4%) – Australia, China, Colombia, Ecuador, Indonesia, Malaysia, Mexico, and the United States. These countries hold a third of global diversity in these vertebrate groups, partially explaining why they concentrate most of the losses. Yet, other megadiverse countries – most notably Brazil (responsible for 10% of species but just 1% of deterioration), plus India and Madagascar – performed better in conserving their share of global vertebrate diversity. Very few countries, mostly island nations (e.g. Cook Islands, Fiji, Mauritius, Seychelles, and Tonga), have achieved net improvements. Per capita wealth does not explain these patterns, with two of the richest countries – United States and Australia – fairing conspicuously poorly. Different countries were affected by different combinations of threats. Reducing global rates of biodiversity loss will require investment in the regions and countries with the highest responsibility for the world's biodiversity, focusing on conserving those species and areas most in peril and on reducing the drivers with the highest impacts.

## Introduction

Increasing awareness about the accelerating rates of species extinctions [1] had prompted the 193 governments Parties to the Convention on Biological Diversity (CBD) to commit in 2002 to “achieve by 2010 a significant reduction of the current rate of biodiversity loss” [2]. This target was missed, as the mounting pressure from threats to biodiversity surpassed the conservation efforts to reduce them [3]. As a response, governments have recently agreed to an ambitious strategic plan for biodiversity, aiming at inspiring broad-based action by all countries and stakeholders in support of biodiversity between 2010 and 2020 [4]. This plan includes a new target 12 stating that “by 2020 the extinction of known threatened species has been prevented and their conservation status, particularly of those most in decline, has been improved and sustained”.

Global biodiversity targets are important in raising awareness among the public and policy makers, auditing management actions, and informing policy choices [5], but only if progress towards them can be rigorously quantified [6]. The International Union for Conservation of Nature (IUCN) Red List Index [7]–[9] is one of the indicators developed within the context of the 2010 target, and is now key for monitoring advances towards target 12 of the new strategic plan. This index is an aggregated measure of species extinction risk as assessed for the IUCN Red List of Threatened Species [10], based on genuine deteriorations (species moving closer to extinction) or improvements (reduced extinction risk) in species' status between time periods. A negative slope in the Red List Index occurs when deteriorations exceed improvements, corresponding to acceleration in the aggregated biodiversity loss for the group. Recent studies revealed a mean decline in the Red List Index in recent years of 0.02% per year for birds, 0.07% for mammals, and 0.14% for amphibians, hence an acceleration in the rates of loss for these taxa [7].

Given that biodiversity is not evenly distributed across space [11]–[13], and neither are the drivers of its decline [14],[15], nor the resources available to its conservation [16], there should be substantial spatial variation in biodiversity trends. This is confirmed by applications of the IUCN Red List Index at national scales, calculated from changes in species' threat status according to national red lists. National red list indices have, for example, revealed a recent deterioration in the conservation status of birds in Finland [17] and in Australia [18], but an improvement in Denmark [19] and in China [20].

These country-level indices are useful for monitoring national biodiversity targets, but they are of limited value to informing progress towards global biodiversity targets. Indeed, they do not take into account the fact that different countries have different levels of global responsibility towards the conservation of the species they harbour. For example, the return of the Osprey *Pandion haliaetus* to Denmark as a breeding species contributed to this country's improving national red list index [19], but was inconsequential to the global Index, because Denmark holds a tiny fraction of this widespread species' population. In contrast, an improvement in the conservation status of Albert's Lyrebird *Menura alberti* in Australia (from Vulnerable to Near Threatened) [18] is of global significance, because this species cannot be found anywhere else. Counter-intuitively, a country can have an improving national index while making a negative contribution to the global Red List Index, if improvements concern mainly species that are marginaly represented within the country and deteriorations species for which the country is highly responsible.

Failure to meet the 2010 global biodiversity cannot therefore be subsumed to a collection of failures to meet the corresponding national targets. But wheras attention to biodiversity indicators at the national [17]–[20] and even sub-national [21],[22] levels is increasing, there is little understanding of conservation performance at these spatial scales adds up to the global trajectories of biodiversity loss.

Here, we present a spatially-explicit assessment of progress towards reducing global rates of biodiversity loss, by quantifying the contribution of different regions and countries towards recent global trends in vertebrate conservation status [7]. Our results highlight lessons from the failed 2010 target and provide guidance for future conservation strategies, highlighting regions and countries likely to play a crucial role for meeting target 12 under the new 2020 strategic plan. Now – when governments still have 6 years to implement it – is the time to provide such guidance.

## Materials and Methods

### Species data

We obtained data on the spatial distribution of three vertebrate groups – 9800 birds (including 1234 classified as threatened), 5393 mammals (1141 threatened) and 6123 amphibians (1885 threatened) from the IUCN Red List of Threatened Species [10] – mapped as polygons representing species' extent of occurrence. We focus on these comprehensively assessed taxonomic groups as surrogates for broader vertebrate diversity because we understand trends in their conservation status far better than for other taxa.

### Economic data

Data on the Gross Domestic Product based on purchasing power parity per capita were obtained from the 2010 World Economic Outlook database [23]. We also obtained allocations for biodiversity under the Global Environment Facility fifth replenishment (2010–2014), under the System for Transparent Allocation of Resources (STAR) [24].

### Changes in species' IUCN Red List categories

For each species, we obtained its Red List category at two time points (the earliest and latest dates for which complete assessments are available for the group): 1988 and 2008 for birds; 1996 and 2008 for mammals; and 1980 and 2004 for amphibians [7]. In each taxon, earlier assessments were corrected in the light of current knowledge, to ensure that changes in Red List status between years correspond to genuine changes in the conservation status of species, rather than being factors such as changes in knowledge, taxonomy, or assessment criteria [7]–[9]. Analyses focused on 887 species that underwent a net change in Red List status: 232 birds (203 deteriorations, 29 improvements), 195 mammals (171 deteriorations, 24 improvements), and 460 amphibians (456 deteriorations, 4 improvements; full list in Table S6 in reference [7]). For each of these species we calculated the number of step changes in Red List category, corresponding to the number of changes between adjacent category levels. The following levels were considered: 0, Least Concern; 1, Near Threatened; 2, Vulnerable; 3, Endangered; 4, Critically Endangered; 5, Critically Endangered (Possibly Extinct), Critically Endangered (Possibly Extinct in the Wild), Extinct in the Wild, or Extinct. ‘Possibly Extinct’ and ‘Possibly Extinct in the Wild’ are tags applied to Critically Endangered species that are likely to be (but not confirmed as) already extinct [25]. Data Deficient species are not included. Negative step changes correspond to deteriorations in Red List status (i.e. increased extinction risk; e.g., changing from Vulnerable to Extinct corresponds to -3 step changes), positive step changes correspond to improvements (i.e. decreased extinction risk). For deteriorating species, we obtained a coding of the relative importance of different threats to the change, classified as primary (believed to have caused>50% of the decline) and secondary (10–49% of the decline). If known, the most important of the secondary drivers (if there was more than one) was identified. See reference [7] for further details.

### Spatial units

We considered three types of spatial units: equal-area hexagons (∼23,322 km^2^) [26]; ecoregions, large biogeographic units covering both land [27] and marine coastal and shelf areas [28]; and countries, including both land area and marine area (within the Exclusive Economic Zone [29]), with territories grouped according to sovereignty (e.g. New Caledonia and Martinique within France). A species was considered present in a given spatial unit (hexagon, ecoregion, or country) whenever its mapped range overlaps the unit. Using Geographic Information Systems, we calculated the area of each species' range *s* within each of the spatial units *u* considered (*r_su_*).

### Weighted change in Red List status per year

For each taxon, the weighted change in Red List status per year in each unit *u*, *W_u_*, was calculated as:
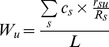
(1)


Where: *C_s_* is the number of step changes in Red List status species *s* undertook; *R_s_* is the species' total range size; *r_su_* is as defined above; *L* is the number of years between assessments (20 for birds; 12 for mammals; 24 for amphibians). The total weighted change in Red List status per year in each unit was then obtained by summing the values for birds, mammals and amphibians. This measures the net contribution of each spatial unit to the global change in vertebrate conservation status (i.e., the unit's relative contribution to the global Red List Index; see Supporting Materials and Methods).

### Weighted threat impact

For each taxon, and in each country *u*, we calculated the weighted impact of each threat, *T_u_*, as:
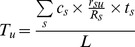
(2)


Where *C_s_*, *R_s_*, *r_su_* and *L* are as defined for equation 1, and *t_s_* is the importance of the threat to the deterioration in species *s*: 1 for primary threats; 0.5 for main secondary; 0.33 for other secondary; and 0 for threats that had no impact on the species. The total weighted threat impact per year in each unit was then obtained by summing the values for birds, mammals and amphibians. This measures the relative importance of a particular threat to the overall deterioration in Red List status in each spatial unit.

### Weighted endemism

The weighted endemism, *E_u_*, of a given country *u* was calculated as:
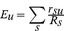
(3)


Corresponding to the sum of the fractions of the species' ranges found within each country (across all mammals, birds and amphibians), this is a measure of the responsibility of each country towards global species conservation.

### Weighted threat

The weighted threat, *R_u_*, of a given country *u* was calculated as:
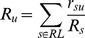
(4)


Where *RL* is the set of species classified as threatened according to the IUCN Red List of Threatened Species (i.e. those classified as Vulnerable, Endangered or Critically Endangered). Corresponding to the sum of the fractions of threatened species' ranges within each country (calculated across all mammals, birds and amphibians), this is a measure of the responsibility of each country towards the conservation of globally threatened vertebrate species.

### Sensitivity analysis to variation in knowledge

We investigated how the results per country would be affected if all Data Deficient (DD) species were threatened, and that their Red List status had changed at the same average rate as the threatened species within each country. We obtained a measure of weigthted endemism in DD species per country *u*, *Du*, using formula 3 but just for the subset of species classified as DD. We assumed all past changes in Red List status to have taken place solely amongst threated species, and calculated a rate of average change per threatened species per country, *C_u_*, as the ratio between *W_u_* and *R_u_*. We then calculated, for each country, the expected rate of change in Red List status (*W_u_*' = *D_u_*.*C_u_*+*W_u_*) and the the expected weighted threat (*R_u_*' = *D_u_*+*R_u_*) if it is assumed that all DD species are threatened.

### Sensitivity analysis to spilage across countries

The calculations of *W_u_*, *T_u_*, and *E_u_*, are based on the assumptions that the species' populations, the intensity of the threats they suffer, and the effectiveness of the conservation actions they benefit from are all uniformly distributed across species' ranges (see Supporting Information S1 for a discussion). Violations of these assumptions may lead to ‘spillage’ (of either blame or merit) between countries, affecting their perceived performance. To investigate if our conclusions on country performance in relation to their share of responsibility are potentially driven by spillage across countries, we repeated the analyses including only single country endemics and single-country near endemics (defined as having ≧70% of their global range in a single country; see Supporting Information S1).

### Dominant threat per country

Countries were coded according to the dominant threat (or combination of threats) driving species' deteriorations in Red List status. This was based on the values of weighted threat impact obtained for each threat in each country, as described above. Agriculture and logging were combined by attributing to each country the maximum of the two. The three dominant threats considered were: agriculture and logging; hunting; and invasive species. A threat was classified as dominant whenever its relative impact was ≧66% of the sum of weighted threat values across the three types of threats. A balanced mix of threats corresponds to a situation where each threat type accounts for ≧25% of the total (none of the countries highlighted is in this situation). For all other cases, a combination of threats was considered based on the two most important threats.

## Results and Discussion

**Figure 1:**
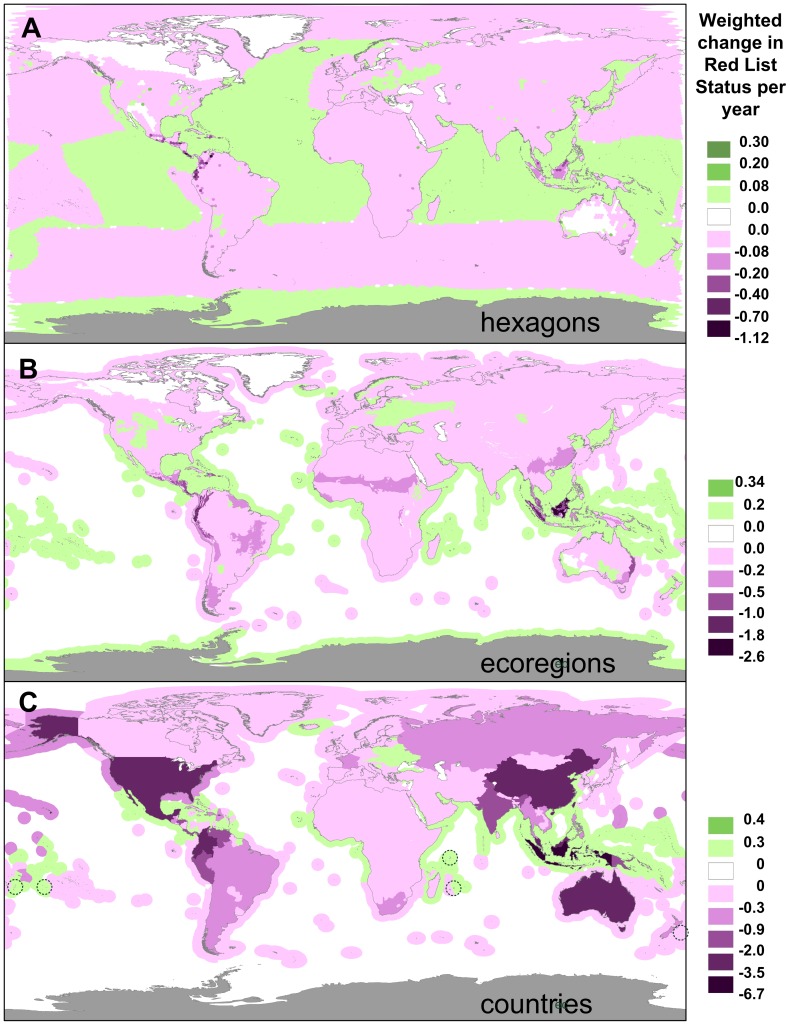
Spatially explicit trends in the global conservation status of vertebrates. Mapped as the variation across space in the weighted change in Red List status per year, across three types of spatial units: hexagons (equal-area, ∼23,322 km^2^), ecoregions (large biogeographic units). and countries (grouped according to sovereignty; e.g. New Caledonia within France). Magenta corresponds to spatial units that have made a net negative contribution to the global vertebrate conservation status as measured through the Red List Index, green to units that made a net positive contribution; grey regions (Antarctica) have no species. In (C), the island nations of Tonga, Cook Islands, Seychelles, Mauritius and Fiji (all with positive weighted change in the Red List status per year) are indicated by a dashed circle.

The results show that although most of the world's regions and countries have contributed negatively to the global trends (Figure 1), in some areas change has been positive. The best examples are a few island nations – Cook Islands, Fiji, Mauritius, Seychelles, and Tonga (Figure 1C) – which have produced some of the most remarkable success stories in the history of the conservation movement [25]. In Mauritius alone, six endemic bird species have undergone substantial improvements in their conservation status between 1988 and 2008 (Table S1 in Supporting Information S1) through a combination of conservation measures including habitat protection and restoration, predator control, captive breeding and supplementary feeding [30].

We found considerable spatial variation in the trends within countries and regions (Figure 1). Most noticeable was the contrast between the land and marine territories within each country or region: compared with land, where changes are mostly negative, net marine changes in Red List status are positive across most countries and regions. However, these results need to be interpreted with caution, because they are explained mainly by recoveries of two very widespread species, Humpback Whale *Megaptera novaeangliae,* and Blue Whale *Balaenoptera musculus*. These are real conservation successes, resulting from regulation of commercial whaling [31], but the fraction of the range of these species within any single Economic Exclusive Area or marine ecoregion is very small, and so they count little to the weighted change in the Red List status. Hence, despite the extensive marine area showing net improvements at all spatial scales (Figure 1), these were modest in intensity when compared with deteriorations in some areas such as the seas around the Galápagos (driven mainly by deterioration in the status of Galápagos Fur Seal *Arctocephalus galapagoensis* and Galápagos Sea Lion *Zalophus wollebaeki*, associated with local impacts compounded by an increased frequency of El Niño events) [32]. The net marine result is actually negative: of the 27 marine birds and mammals which changed Red List status, only five correspond to improvements.

**Figure 2:**
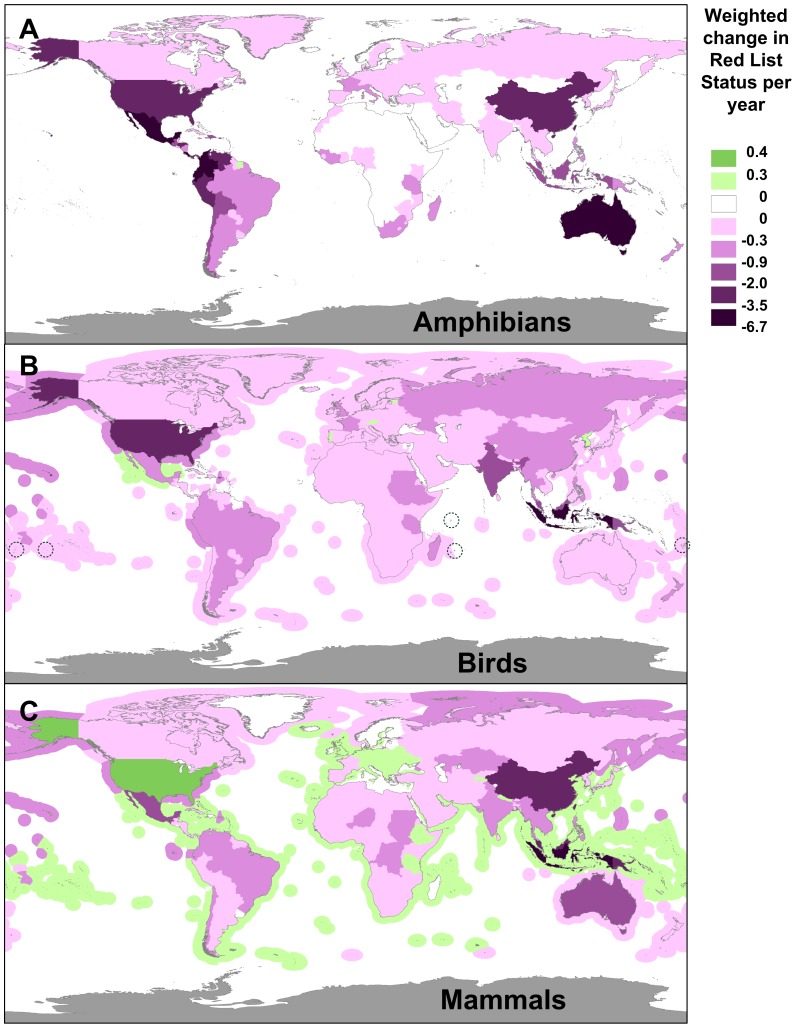
Country contributions towards trends in the global conservation status of amphibians, birds and mammals. Mapped as the variation across countries in the weighted change in Red List status per year, for different taxonomic groups. The same color scale is used across panels (and in Figure 1C) so shades are directly comparable. In (B), the island nations of Tonga, Cook Islands, Seychelles, Mauritius and Fiji are indicated by a dashed circle.

Patterns of change differed between the three taxa (Figure 2 and Figures S1, S2 in Supporting Information S1). In the United States, for example, a net improvement for mammals (three improvements in land species; Figure 2C) was offset by substantial deterioration in the status of amphibians (29 deteriorations; Figure 2A) and birds (26 deteriorations, particularly in Hawaii; Figure 2B). Most deteriorations in Mexico, Colombia and Australia are also amphibians (Figure 2A), whereas deteriorations in Indonesia are associated with changes in the conservation status of birds and mammals (Figs. 2B, 2C).

**Figure 3:**
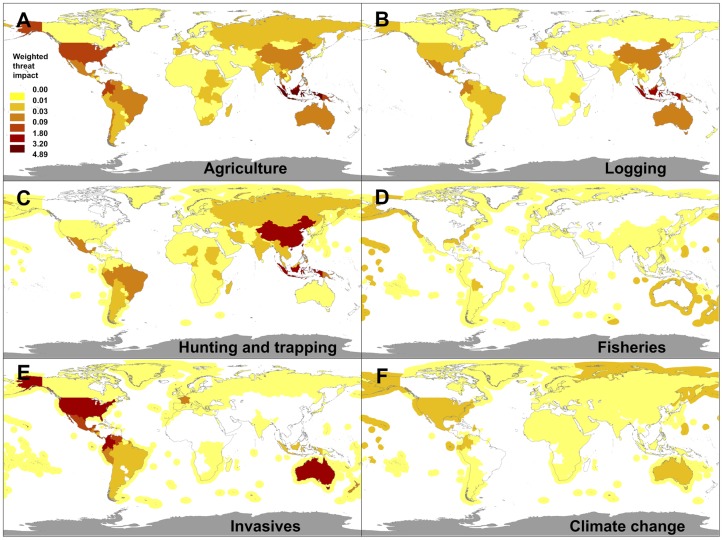
Contribution per country of different threats to the trends in the global conservation status of vertebrates. Mapped as the weighted impact of each threat type to the deterioration in global species conservation status, across countries. The same color scale is used across panels, hence shades are directly comparable.

A diversity of threats contributed to the global deterioration in the status of species, but their relative impact varied spatially (Figure 3 and Figures S3, S4 in Supporting Information S1). The weighted impact of a particular threat in a country measures the relative importance of the threat to deteriorations in combined Red List status in the country (Materials and Methods). Agriculture and logging were the main drivers of biodiversity loss in Southeast Asia [33], particularly Indonesia and Malaysia (170 deteriorations, mostly among birds and mammals). Hunting and trapping – for food, traditional medicine and the pet trade – has been most marked in Asian countries [33], particularly China and Indonesia (109 deteriorations). Invasive species – including predators (e.g. rats, cats, foxes), habitat modifiers (e.g. pigs, rabbits), and pathogens (e.g. avian malaria, amphibian chytrid fungus) are a major threat in oceanic islands (e.g., Hawaii, 11 deteriorations) and Australia (46 deteriorations). The same is true in the tropical Andes (Colombia, Ecuador; 69 deteriorations) and Central America (Costa Rica, Panama; 31 deteriorations) where chytridiomycosys is probably a leading cause for most status deteriorations among amphibians [11].

**Figure 4:**
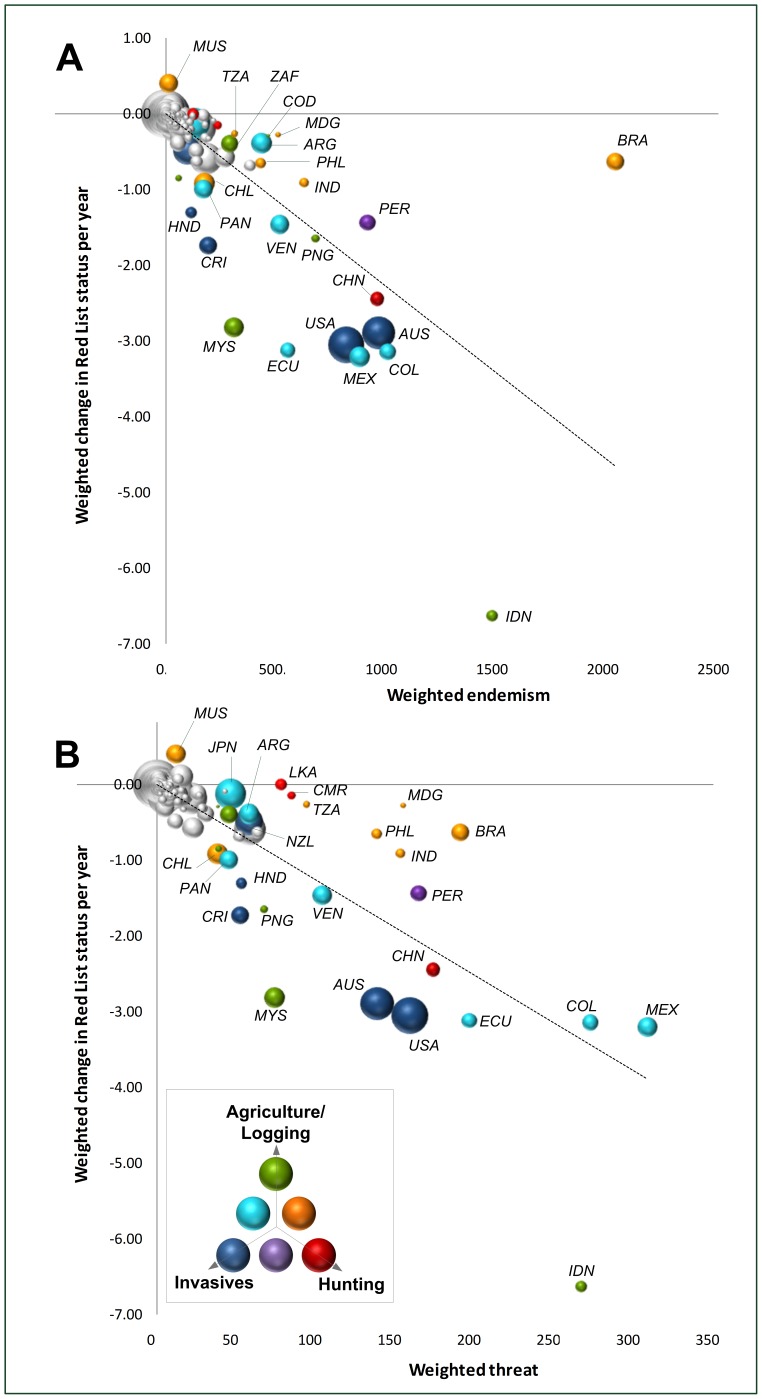
Relationship between each country's responsibility to conservation and its contribution to changes in species' global conservation status. Responsibility measured by: (A) weighted endemism; (B) weighted threat. Dashed lines are the regression lines fitted through the origin: (A) *R^2^* = 0.57; (B) *R^2^* = 0.70 (*n* = 195; *p*<0.001). Circle size is proportional to each country's Gross Domestic Product based on purchasing-power-parity per capita GDP in 2009 [23]. Circle color indicates the main threat (or combination of threats) for selected countries (see Table S2 in Supporting Information S1 for country codes).

The losses highlighted here are concentrated in just a small fraction of the world's area: more than 50% of the total net change in Red List status is driven by changes in less than 1% of the Earth's surface (marine and terrestrial combined; Figure 1A), covering only 39 ecoregions (4%; mainly found in Central America, the Tropical Andes, Southeast Asia and eastern Australia, Figure 1B) and just eight countries – Australia, China, Colombia, Ecuador, Indonesia, Malaysia, Mexico, and the United States (4%; Figure 1C). The distribution of these losses is naturally related to the underlying ecological patterns: regions with more unique biodiversity stand to lose more. Accordingly, there is a negative relationship between countries' weighted change in Red List status and weighted endemism – a measure of their global share of, and responsibility towards, the conservation of birds, mammals and amphibians, calculated as the country's species richness weighted by the proportion of each species' range that falls within the country (Figure 4A; Materials and Methods). However, all eight countries mentioned above perform worse than expected from their responsibility: they hold 33% of the world's diversity of species in these groups yet registered 53% of the losses. In contrast, Brazil, the Democratic Republic of Congo, India, and Peru perform above expectations (together holding 23% of overall diversity, 8% of losses).

There is an even stronger negative relationship between countries' weighted change in Red List status and weighted threat – a measure of their current contribution to the global pool of threatened species (Figure 4B; Materials and Methods). Countries with the highest levels of weighted threat are generally also experiencing the highest rates of biodiversity loss. However, some countries – most noticeably Brazil, Cameroon, India, Madagascar, Sri Lanka, the Philippines, and Tanzania – have contributed less to recent global losses than would be expected based on their current levels of threat (21% of threatened species diversity, 6% of losses). In contrast, Indonesia, Malaysia, Australia, Costa Rica and the United States are increasing their levels of threat at a faster rate than expected (16% of threatened species, 33% of losses).

Perhaps unexpectedly, countries with the highest economic capacity are not performing better than others in conserving their share of global biodiversity (Figure 4 and Figure S5 in Supporting Information S1). The relationship between weighted change in Red List status and the Global Domestic Product (GDP) per capita [23] is non-significant (*R^2^* = −0.08) and remains so when controlling for differences in weighted endemism (*R^2^* = 0.003). This result also holds when considering the total size of the economy (GDP) as the measure of economic power (*R^2^* = 0.04 and *R^2^* = 0.01, respectively) (Figure S5 in Supporting Information S1). Indeed, two of the richest countries – United States and Australia – were amongst the worst performers, while some of the poorest – Madagascar, the Democratic Republic of Congo, and Tanzania – were amongst the best.

Whereas the threats primarily responsible for declines vary widely among the worst performers (Figure 4 and Figure S5 in Supporting Information S1), it is notable that invasive species have been the main culprits in the wealthiest such countries.

Knowledge about species' conservation status is far from perfect, and likely to be more incomplete in poorer than in richer countries. A reflection of this problem are Data Deficient species – those for which when there is inadequate information to assess their conservation status – comprising 15% of mammal, 1% of bird and 35% of amphibian species [7]. As a sensitivity analysis of the effects of knowledge, we investigated a scenario in which all DD species were assumed to be threatened, and all changes in Red List status were assumed to occurr among threatened species (Materials and Methods). In this scenario, the magnitude of change in Red List status would increase substantially, but the relative position of countries would remain generally stable, albeit with an improvement in the relative performance of the United Stated and Australia (Figure S6 in Supporting Information S1). Overall, though, the results above would remain valid, with the relative position of countries economic power still unrelated to performance (relationship between weighted change in Red List status and GDP per capita: *R^2^* = −0.09; when controlling for endemism: *R^2^* = −0.0003).

Information is also much incomplete regarding the variation across space in species' populations, the intensity of the threats they suffer, and the effectiveness of the conservation actions they benefit from. In the absence of such information, we assumed throughout that these are uniform within species' ranges. However, this may lead to ‘spillage’ of either blame or merit between neighbouring countries, when a species' deterioration or improvement in a country is wrongly attributed to another. We found that our results hold even when considering only single-country endemics or near endemics (Figure S7 in Supporting Information S1), for which there is little or no ambiguity as to which country holds the responsibility for their conservation. The two main differences in performance were for Australia, that performs even worse, and for Indonesia, that performes substantially better (likely because the analyses exclude a number of species shared with Malaysia, which also appears as performing less badly). Furthermore, a spillage effect is likely to substantially affect the patterns of change in marine areas, as some species have very wide ranges (shared by many countries) within which a few key areas (e.g. for feeding or reproduction) are disproportionately responsible for species' conservation.

## Conclusions

The weighted change in Red List status per year captures one facet of biodiversity loss, but even countries with a good performance by this metric may be losing biodiversity fast in terms of declines in abundance and local extirpations. Other indicators such as national red list indices or national adaptations of the Living Planet Index [34] are needed to capture these changes. Conversely, though, a good performance under national biodiversity indicators is not necessarily evidence of good performance towards global targets (e.g. the has been an improvement in the national red list index for birds in China [20] but we show that this country has had a net negative impact on the global changes in Red List Index for birds; Figure 2B). We provide a methodology countries can use as an additional indicator to report against target 12, as required under Article 26 of the Convention on Biological Diversity.

Our results show that a very small proportion of the world accounts for a disproportionate share of recent global biodiversity losses as measured through the Red List Index (Figure 1). At fine spatial scales, we found a familiar pattern highlighting mainly biodiversity-rich economically-poor countries (mainly in Central America, Tropical Andes, and Southeast-Asia). At the country level, though, we found no relationship between economic weatlth and conservation performance, with the United States and Australia among the countries that contributed the most to the global decline in the Red List Index. This suggests economic development does not automatically guarantee a country's effectiveness in conserving the biodiversity it is globally responsible for.

Looking forward, meeting the globally agreed targets of preventing extinction and improving species' conservation status will require focused conservation investment in countries with larger shares of responsibility for global biodiversity. The measure of weighted endemism we propose here provides a straightforward method for quantifying such share in responsibility, whreas our measure of weighted threat highlights countries that are already substantially burdened with threatened species. Meeting target 12 will necessarily require strategic conservation investment in these countries.

Although most conservation investment takes place within economically rich countries [16], funding mechanisms such as the Global Environment Facility (GEF) [35] can have a disproportionate impact on biodiversity-rich, economically-poor, countries [36]. Appropriately, GEF allocations to biodiversity closely reflect the share of each country's responsibility (for the period 2010–2014, correlation between GEF allocations [24] and weighted endemism, *R^2^* = 0.87; *n* = 98 countries eligible for GEF funding). Moreover, resource allocation through the GEF also incorporates rewards for high performance, important not only in maintaining the successes of those few countries that are meeting biodiversity targets (e.g., the island nations highlighted here) but also in incentivizing other countries to improve. We proposed that weighted endemism be used to inform future GEF allocations across countries.

Meeting global biodiversity targets also requires ensuring that within each country conservation is implemented strategically [37]. This requires: focusing on those species for which the country is solely or largely accountable, targeting the exact sites where conservation can make most difference (particularly sites holding the last populations of highly threatened species) [38], and addressing the specific threats that are driving biodiversity loss. Threatening processes such as habitat loss (e.g., in Indonesia and Malaysia) and overexploitation (e.g., in China) have well-known solutions (e.g., habitat protection, sustainable harvesting), and these must be implemented urgently and strategically. While some types of invasive species are currently challenging to address even for the wealthiest countries (e.g., avian malaria in the United States; amphibian chytrid fungus in Australia), there is no justification for lack of action, both the implementation of existing approaches (e.g. *ex-situ* conservation, biological control) and through investment in the development of new solutions (e.g. through biomedical research). Hundreds of species worldwide (e.g. many bird species endemic to Mauritius, Seychelles and New Zealand) are living examples of how previously apparently insurmountable challenges can be overcome through focused conservation efforts [7],[25].

## Supporting Information

Supporting Information S1 **Supporting Materials and Methods. Figure S1,** Variation across hexagons in the weighted change in Red List status per year, for different taxonomic groups. **Figure S2,** Variation across ecoregions in the weighted change in Red List status per year, for different taxonomic groups. **Figure S3,** Weighted impact of each threat to the deterioration in global species conservation status, across hexagons. **Figure S4,** Weighted impact of each threat to the deterioration in global species conservation status, across ecoregions. **Figure S5,** Relationship between each country's responsibility to conservation and its contribution to changes in the global conservation status of birds, mammals and amphibians. **Figure S6,** Sensitivity to variation in knowledge of the relationship between each country's responsibility to conservation and its contribution to changes in species global conservation status. **Figure S7,** Sensitivity of the results to possible spilage across countries. **Table S1,** Absolute weighted Red List change per country, and list of the species driving those values. **Table S2,** Main results per country.
(ZIP)
