# Correction: Spatially Explicit Trends in the Global Conservation Status of Vertebrates

**DOI:** 10.1371/journal.pone.0121040

**Published:** 2015-03-20

**Authors:** The PLOS ONE Staff

Supporting Information File Rodrigues_etal_Supporting Information S1 with Track Changes.doc should not appear with the published article. Please view the correct Rodrigues_etal_Supporting Information S1 here.

## Supporting Information

S1 File Rodrigues_etal_Supporting Information. Supporting Materials and Methods.
(DOC)
