# Supplementary material for: Spatially Explicit Trends in the Global Conservation Status of Vertebrates
Source: PLoS One. 2014 Nov 26;9(11):e113934. doi: 10.1371/journal.pone.0113934 (PMC4245261; doi:10.1371/journal.pone.0113934)
Supplement: Supporting Information S1 — Supporting Materials and Methods. Figure S1, Variation across hexagons in the weighted change in Red List status per year, for different taxonomic groups. Figure S2, Variation across ecoregions in the weighted change in Red List status per year, for different taxonomic groups. Figure S3, Weighted impact of each threat to the deterioration in global species conservation status, across hexagons. Figure S4, Weighted impact of each threat to the deterioration in global species conservation status, across ecoregions. Figure S5, Relationship between each country's responsibility to conservation and its contribution to changes in the global conservation status of birds, mammals and amphibians. Figure S6, Sensitivity to variation in knowledge of the relationship between each country's responsibility to conservation and its contribution to changes in species global conservation status. Figure S7, Sensitivity of the results to possible spilage across countries. Table S1, Absolute weighted Red List change per country, and list of the species driving those values. Table S2, Main results per country. (ZIP) [file pone.0113934.s001.zip › Rodrigues_etal_Supporting Figure S1.pdf]

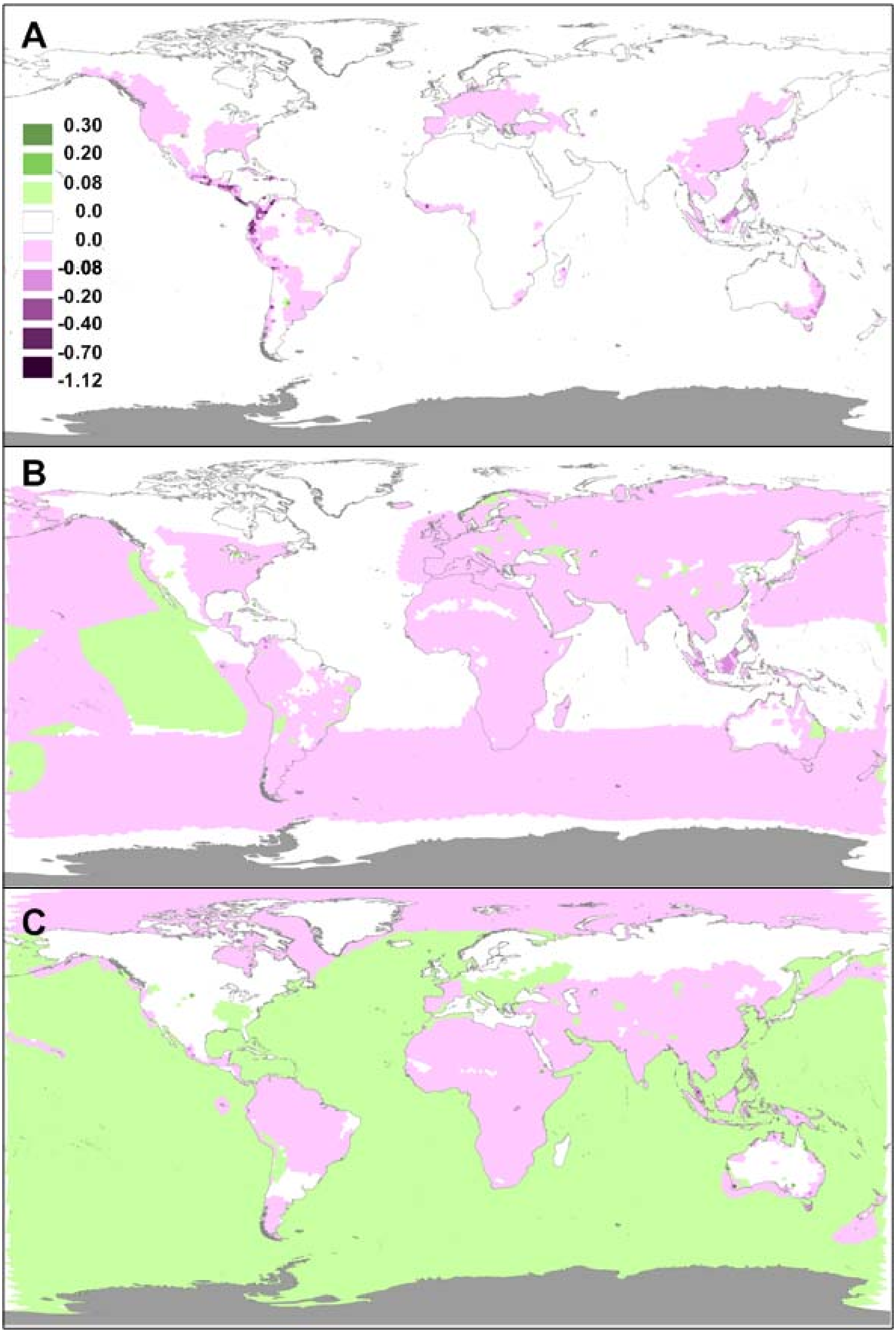

Figure S1. Variation across hexagons in the weighted change in Red List status per year, for different taxonomic groups: (A) amphibians; (B) birds; (C) mammals. The same color scale is used across panels (and in Figure 1A) so shades are directly comparable.
