# Supplementary material for: Spatially Explicit Trends in the Global Conservation Status of Vertebrates
Source: PLoS One. 2014 Nov 26;9(11):e113934. doi: 10.1371/journal.pone.0113934 (PMC4245261; doi:10.1371/journal.pone.0113934)
Supplement: Supporting Information S1 — Supporting Materials and Methods. Figure S1, Variation across hexagons in the weighted change in Red List status per year, for different taxonomic groups. Figure S2, Variation across ecoregions in the weighted change in Red List status per year, for different taxonomic groups. Figure S3, Weighted impact of each threat to the deterioration in global species conservation status, across hexagons. Figure S4, Weighted impact of each threat to the deterioration in global species conservation status, across ecoregions. Figure S5, Relationship between each country's responsibility to conservation and its contribution to changes in the global conservation status of birds, mammals and amphibians. Figure S6, Sensitivity to variation in knowledge of the relationship between each country's responsibility to conservation and its contribution to changes in species global conservation status. Figure S7, Sensitivity of the results to possible spilage across countries. Table S1, Absolute weighted Red List change per country, and list of the species driving those values. Table S2, Main results per country. (ZIP) [file pone.0113934.s001.zip › Rodrigues_etal_Supporting Information S1.pdf]

# Supporting Information S1:
# Supporting Materials and Methods

**Species spatial data.** We obtained data on the spatial distribution of three vertebrate groups – 9800 birds (including 1234 classified as threatened), 5393 mammals (1141 threatened) and 6123 amphibians (1885 threatened) from the IUCN Red List of Threatened Species [S1] – mapped as polygons representing species' extent of occurrence. We focus on these comprehensively assessed taxonomic groups as surrogates for broader vertebrate diversity because we understand trends in conservation status far better than for other taxa.

Spatial data correspond to species' extent of occurrence: polygons that are coarse generalizations of species' distributions, generally obtained as 'envelopes' including original records and through interpolation from original records. They may include relatively extensive areas from which the species is absent (e.g., terrestrial habitats within a freshwater species' range) and are therefore likely to overestimate the species' true area of occupancy. See the Supporting Online Material in reference S2 (section 1) for further details.

Individual polygons are coded according to species' presence (1 – extant; 2 – probably extant; 3 – possibly extant; 4 – possibly extinct; 5 – extinct), origin (1 – native; 2 – reintroduced; 3 – introduced; 4 – vagrant; 5 – origin uncertain) and seasonality (1 – resident; 2 – breeding season; 3 – non-breeding season; 4 – passage; 5 – seasonal occurrence uncertain). These categories are described in the metadata file that accompanies the shapefiles, both downloadable from reference S1.

**Species conservation status.** For each species, we obtained its Red List category at two time points (the earliest and latest dates for which complete assessments are available for the group): 1988 and 2008 for birds; 1996 and 2008 for mammals; and 1980 and 2004 for amphibians [S2]. In each taxon, earlier assessments were corrected in the light of current knowledge, to ensure that changes in Red List status between years correspond to genuine changes in the conservation status of species, rather than being factors such as changes in knowledge, taxonomy, or assessment criteria [S2, S3]. See the Supporting Online Material in reference S2 (sections 1.3 and 2.4) for detail on method followed for distinguishing between

genuine from non-genuine changes in conservation status. Analyses focused on 887 species that underwent a net change in Red List status: 232 birds (203 deteriorations, 29 improvements), 195 mammals (171 deteriorations, 24 improvements), and 460 amphibians (456 deteriorations, 4 improvements; the full list is presented in Table S6 in reference S2). For each of these species we calculated the number of step changes in Red List category, corresponding to the number of changes between adjacent category levels. The following levels were considered: 0, *Least Concern*; 1, *Near Threatened*; 2, *Vulnerable*; 3, *Endangered*; 4, *Critically Endangered*; 5, *Critically Endangered (Possibly Extinct), Critically Endangered (Possibly Extinct in the Wild), Extinct in the Wild*, or *Extinct*. 'Possibly Extinct' and 'Possibly Extinct in the Wild' are tags applied to *Critically Endangered* species that are likely to be (but not confirmed as) already extinct [S4]. *Data Deficient* species were not included [S5, S6]. Negative step changes correspond to deteriorations in Red List status (i.e. increased extinction risk; e.g., changing from *Vulnerable* to *Extinct* corresponds to -3 step changes), positive step changes correspond to improvements (i.e. decreased extinction risk).

**Species' threat data.** For deteriorating species, we obtained a coding of the relative importance of different drivers to the change in species' Red List status, classified as primary (believed to have caused >50% of the decline) and secondary (10-49% of the decline). If known, the most important of the secondary drivers (if there was more than one) was identified. See reference S2 (their Supporting Online Material, section 2.7 and Table S6) for further details and a full list of threats per species. We considered the following threats [S1], responsible for most of the changes in species' conservation status:

- Agriculture: including annual and perennial non-timber crops, wood and pulp plantations, livestock farming and ranching, and marine and freshwater aquaculture.
- Logging: including both large and small scale (subsistence) harvesting, and including incidental or accidental mortality.
- Hunting and trapping: including both large and small scale (subsistence) harvesting, and including incidental or accidental mortality.
- Fisheries: including both large and small scale (subsistence) harvesting, and including incidental or accidental mortality.
- Invasive species: including alien species as well as problematic native species, and including the pathogens responsible for most diseases.

- Climate change: including severe weather events (e.g. droughts and temperature extremes), as well as habitat shifting and alteration presumed to be associated with climate change.

Note that "hunting or trapping" refers to an inland activity while "fisheries" refers to an activity that takes place in marine or freshwater systems. Maps showing an effect of fisheries over land (e.g. Figure 3D) correspond to freshwater fisheries. Maps showing an effect of hunting and trapping over the sea (Figure 3C), on the other hand, correspond to marine species that are hunted inland. For example, the Waved Albatross *Phoebastria irrorata* is threatened by harvesting for meat, feathers and eggs in its nesting grounds, and this is coded as hunting or trapping within Ecuador's Economic Exclusive Area (Figure 3C). The species is also threatened by bycatch in longline fisheries, and this is coded as fisheries within Ecuador's Economic Exclusive Area (Figure 3D). See the Threats Classification Scheme in reference S1 for further details.

**Taxonomic classification.** The taxonomic classification used for each group corresponds to that followed in the respective Red List assessments (2008 for birds and mammals, 2004 for amphibians; see section 1.3 in the Supplementary Online Material of reference S2). Subsequent changes are not taken into account in the results. For example, the Blue Poison Frog *Dendrobates azureus* was one of the few cases of conservation improvement in amphibians, having moved from Critically Endangered in 1980 to Vulnerable in 2004, as captive breeding removed over-collection threat. However, this taxon has been synonymized with *D. tinctorus* in 2006 [S7] and so it will be removed from future iterations of the Red List Index.

**Spatial units.** We considered three types of spatial units: hexagons (8); ecoregions; and countries. The hexagonal grid is composed of individual units that retain their shape and area (~23,322 km$^2$) throughout the globe, corresponding to a fine-scale partition of space into equal-sized units that have no biological or political meaning. Hexagons were obtained from a geodesic discrete global grid system, defined on an icosahedron and projected to the sphere using the inverse Icosahedral Snyder Equal Area (ISEA) Projection [S8]. Hexagons that crossed the coastline where clipped into marine and land fractions. A row of cells near latitude 180°E/W was excluded, as these interfered with the spatial analyses. This creates an

artificial narrow band of no data in the hexagon maps. Ecoregions are large biogeographic units covering both land [S9] and marine coastal and shelf areas [S10]. They correspond to ecological units, containing distinct assemblages of natural communities and species [S9]. Countries correspond to political units, with territories grouped according to sovereignty (e.g. New Caledonia and Martinique within France). The marine area of each country corresponds to the Exclusive Economic Zone [S11].

**Economic data.** Data on the Gross Domestic Product based on purchasing power parity per capita were obtained from the 2010 World Economic Outlook database [S12]. We also obtained allocations for biodiversity under the Global Environment Facility fifth replenishment (2010-2014), under the System for Transparent Allocation of Resources (STAR) [S13].

**Mapping species to spatial units.** A species is considered present in a given spatial unit (hexagon, ecoregion, or country) whenever its mapped range overlaps the unit. Using Geographic Information Systems, we calculated the area of each species' range $s$ within each of the spatial units $u$ considered ($r_{su}$). Species whose ranges are over land (including freshwater species) were mapped to land spatial units (or the land portion of coastal hexagons), while species whose ranges are over the sea were mapped separately to marine units (or the land portion of coastal hexagons). Some species had substantial ranges that crossed both land and marine realms, and consequently were mapped both into marine and into land spatial units.

As mentioned above, the original species distributional data was obtained as polygons mapped according to presence, origin and seasonality. For the calculations of weighted endemism and weighted threat (see below), only the following polygons were included: where the species was both reported as native or reintroduced (origin coded as 1 or 2) and currently extant or probably extant (presence coded as 1 or 2) and where seasonality was coded as resident, breeding season, non-breeding season, and passage migrant (seasonality coded as 1, 2, 3, 4). This thus excludes historical and introduced ranges as well as where origin is uncertain as well as vagrancies and instances in which seasonal occurrence is unknown.

For the calculations of weighted change in Red List status (see below), we considered somewhat different parts of the species' ranges. The main difference relates to the treatment of parts of the range where the species is extinct or probably extinct: while these were excluded from the calculations of weighted endemism and weighted threat (which aim to provide information about current diversity in different spatial units) they were included in the mapping of weighted change in Red List status, for those species that deteriorated in conservation status, if the extinction or possible extinction of the species from those parts of the range took place within the period considered. For example, the Yangtze River Dolphin or Baiji, *Lipotes vexillifer*, is now possibly extinct throughout all of its former range in China and this possible extinction has likely taken place in the 1996-2008 period [S1]. This species has thus not been considered for the purposes of quantifying current endemism and threat, but it contributes negatively to China's value of weighted change in Red List status. More specifically, the following parts of species' range maps were excluded when calculating weighted change in Red List status: where the species is vagrant (polygons coded as origin 4) or of uncertain origin (origin = 5); where the species is introduced, except if such introduction was done for conservation purposes (e.g. translocation of Mauritius Fody *Foudia rubra* to predator-free Ile aux Aigrettes); where the species is possibly extant but there are no known records (presence = 3); where the species is coded as probably extinct (presence = 4) or extinct (presence = 5) if the species is improving in Red List Status, because it is assumed that these are areas of previous decline where recovery has not yet occurred (e.g., most of the historical range of the Black-footed Ferret *Mustela nigripes*, outside the three small areas where it has established new populations subsequent to successful reintroduction programs); where a deteriorating species is coded as extinct (presence = 5) if and only if such range contraction took place before the temporal period considered (e.g., before 1980 for amphibians); where the species' seasonal occurrence is uncertain (seasonality = 5).

When mapping the weighted change in Red List status (Figure 1), values for marine and land spatial units were derived separately. When calculating an overall value per country (y-axis in Figure 4), the land and marine (Exclusive Economic Zone) territories were combined before calculations of the fraction of each species' range that falls within each country.

**Weighted change in Red List status per year.** This is a measure of the net contribution of each spatial unit to the global annual change in vertebrate conservation status. For example, a

value of $W_u$ = -1 may correspond to: an average of a species wholly contained within the spatial unit (hexagon, country or ecoregion) deteriorating one step per year; or an average of two species with 50% of their ranges within the unit deteriorating one step per year; or an average of 100 species with 1% of their ranges within the unit deteriorating one step per year. Widespread species contribute little to the value in each site (e.g., Humpback Whale *Megaptera novaeangliae*, as mentioned in the main text).

For each taxon, calculating the numerator in equation 1 corresponds to distributing across space the total number of step changes (net value: -226 for birds, -156 for mammals and -662 for amphibians), such that this is done proportionately to the fraction of each species' range in each spatial unit. $W_u$ is then obtained by dividing this weighted number of steps by the total number of years in the period considered (20 for birds, 12 for mammals and 24 for amphibians). This creates a standardized value per year than can be summed across taxa. For all three types of spatial units considered, then, the sum of *Wu* values across all units is equal to -11 for birds (-226 net step changes/20 years), -13 for mammals (-156/12) and -28 for amphibians (-662/24).

For any given taxon, maps of *Wu* represent perfectly the relative contribution of each spatial unit to the overall change in the Red List Index for that taxon. Indeed, the exact value of change in the Red List Index can be derived by summing *Wu* across all spatial units, multiplying it by the length of the time period considered (to obtain again the absolute number of step changes), and then dividing this by the number of data-sufficient species in the taxon. Each of the maps of *Wu* (Figs 1, 2, S1, S2) is therefore a spatially-explicit representation of change in the Red List Index.

Calculation of *Wu* assumes the responsibility of each spatial unit for the change in a species' Red List status is proportional to the fraction of each species' range inside the unit. In practice, this corresponds to assuming that changes in Red List status happen uniformly across each species' range. This is not always true in reality, and may result in "spillage" (of either blame or merit) between countries. In Figure 2C, for example, Nepal is represented in green, indicating a net positive contribution to the global change in Red List status for mammals. This result is explained by the improvement in the conservation status of the Greater One-horned Rhino *Rhinoceros unicornis* from Endangered to Vulnerable, and the fact that 50% of this species' mapped range falls within Nepal. However, the credit for the

improvement in the Rhino’s status should mainly be attributed to India, rather than equally to both countries, as India holds 85% of all individuals, including the best protected populations [S1]. Calculation of *Wu* could therefore be refined by integrating more refined information on the actual relative contribution of each country to species’ changes in Red List status; in the absence of such information, the fraction of each species’ range in each country is a reasonable approximation.

**Weighted threat impact.** This measures the relative importance of a particular threat to the overall deterioration in Red List status in each spatial unit. A value of weighted threat impact of $T_u$ = -1 may correspond, for example: to a threat responsible as a primary threat to an average deterioration of one step per year in a species wholly contained within the spatial unit (hexagon, country or ecoregion); or to a threat responsible as a secondary threat to an average deterioration of one step per year in two species wholly contained within the spatial unit (hexagon, country or ecoregion). Widespread species contribute little to the value in each site, as do least important threats.

As with the weighted change in Red List status, there may be spillage between countries due to the assumption that threats affect each species’ uniformly across their ranges. For example, the Blyth's Flying Fox *Pteropus melanotus* occurs in the Andaman and Nicobar Islands (India), in Sumatra (Indonesia), and in Christmas Island (Australia). Hunting and trapping contributed to its deterioration from Near-Threatened in 1996 to Vulnerable in 2008 (1) (and see Table S6 in reference S2). As a result, Australia is mapped in Figure 3C as being affected by hunting and trapping, but this is an example of spillage, as this species is not hunted on Christmas Island [S1].

**Weighted endemism.** This is a measure of the responsibility of each country towards global species conservation. A value of weighted endemism, $E_u$=1 may correspond, for example: to one fully endemic species; or two species with 50% of their range within the country; or 100 species with 1% of their range within the country. The sum of all values of weighted endemism across countries is equal to the total number of species worldwide.

**Weighted threat.** This is a measure of the responsibility of each country towards the conservation of globally threatened vertebrate species. The sum of all values of weighted threat, $R_u$, across countries is equal to the total number of threatened species worldwide.

Weighted endemism and weighted threat measure the responsibility of each country to global conservation, based on the assumption that each country's responsibility to the conservation of each species is proportionately to the fraction of the species' range in the country. If data were available, a more accurate measure of responsibility could be obtained from the fraction of each species' population in each country. For example, as mentioned above, India has 85% of the population of the Greater One-horned Rhino but only 50% of the global range; arguably, India's responsibility for the conservation of this species should therefore be 85% rather than 50%.

**Sensitivity analysis to variation in knowledge.** Countries vary substantially in the state of biological knowledge, including species that are already described but for which conservation status is now known (Data Deficiente, DD) and undescribed species. Given that the number of undescribed species per country is unknown, we have performed a sensitivity analysis of the effects of variation in knowledge on the results by focusing on the DD species. Figure S6 represents the predicted changes per country under a scenario where all DD Species are considered to be threatened and all past changes in Red List status have concentrated on threatened species. This scenario overestimates the effects of lack of knowkedge associated with DD species, because they are not likely to be all threatened [S14] and because not all changes in Red List status were among threatened species (some were between Least Concern and Near-Threatened species, and some where between non-threatened and threatened species). However, this scenario is likely to underestimates the overall effect of lack of knowledge associated with species still to be described, particularly amongst amphibians [S16], but also mammals [S15], for which considerable numbers of new species are expected to still be described.

These two sets of species (DD and undescribed) are somewhat different in how they affect the results. Both DD and undescribed species are likely to affect measures of weighted change in Red List status (i.e., the vertical position of countries in Figs. 4 and S5) and of weighted threat (the horizontal position in Figs. 4B and S5B). However, unlike undescribed species, DD species do not affect the measure of weighted endemism (the horizontal position

in Figs. 4A and S5A) because they were already mapped and so included within the measures of weighted endemism. As a result, in the scenario considered (where DD species were assumed to be threatened) the position of each country moved vertically in the scatterplot of the relationship between weighted change in Red List status and weighted endemism (Figure S6A) but diagonally in the scatterplot of the relationship between weighted change in Red List status and weighted threat (Figure S6B; which explains why the regression line barely changes). For undescribed species, the position of each country would move diagonally in both graphs. It is therefore not easy to predict the effect of undescribed species on the position of countries in relation to the regression line.

**Sensitivity analysis to spilage across countries.** As discussed above, the calculations of weighted change in Red List status, of weighted threat impact, and weighted endemism are based on the assumptions that the species' populations, the intensity of the threats they suffer, and the effectiveness of the conservation actions they benefit from are all uniformly distributed across species' ranges. This is not always the case and may leade to "spillage" (of either blame or merit) between countries, as illustrated with the examples above of the Greater One-horned Rhino (India, Nepal) and Blyth's Flying Fox (India, Indonesia, Australia).

However, for most of the species that have changed Red List status, their populations (and therefore the responsibility for their conservation) is not shared among countries: 55% of these species are single-country endemics and 75% are single-country near-endemics (defined here as having >= 75% of their global range within a single country). We have investigated the extent to which our conclusions on country performance in relation to their share of responsibility (Figure 4A) may be affected by spillage across countries by repeating the analyses for endemic and for near-endemic species (Supplementary Figure S7).

## SI References

S1. IUCN (2010) 2010 IUCN Red List of Threatened Species. Version 2010.3. Available at: http://www.iucnredlist.org.

S2. Hoffmann M et al. (2010) The impact of conservation on the status of the world's vertebrates. Science 330:1503–509.

S3. Butchart SHM et al. (2005) Using Red List Indices to measure progress towards the 2010 target and beyond. Phil Trans Royal Soc Lond B 360:255–268.

S4. Butchart SHM, Stattersfield AJ, Brooks TM (2006) Going or gone: defining "Possibly Extinct" species to give a truer picture of recent extinctions. Bull. B.O.C. 126A:7–24.

S5. Butchart SHM et al. (2004) Measuring global trends in the status of biodiversity: Red List Indices for birds. PLoS Biol 2:e383.

S6. Butchart SHM et al. (2007) Improvements to the Red List Index. PLoS ONE. 2:e140.

S7. Wollenberg KC, Veith M, Noonan BP, Lötters S (2006) Polymorphism versus species richness - systematics of large Dendrobates from the Eastern Guiana Shield (Amphibia: Dendrobatidae). Copeia 4:623–629.

S8. Sahr K, White D, Kimerling AJ (2003) Discrete global grid systems. Geog. Inf. Sci 30:121–134.

S9. Olson DM et al. (2001) Terrestrial ecoregions of the world: A new map of life on Earth. Bioscience 51:933–938.

S10. Spalding M et al. (2007) Marine ecoregions of the world: A bioregionalization of coastal and shelf areas. BioScience 57:573–583.

S11. VLIZ (Vlaams Instituut Voor de Zee) (2010) Maritime Boundaries Geodatabase, Version 5. Available at: http://www.vliz.be/vmdcdata/marbound/download.php [Accessed September 13, 2010].

S12. International Monetary Fund (2010) World Economic Outlook Database (International Monetary Fund, Available at: http://www.imf.org/external/pubs/ft/weo/2010/01/weodata/index.aspx [Accessed September 13, 2010].

S13. GEF (Global Environment Facility) (2009) GEF - 5 Initial Star Allocations (Global Environment Facility, Available at: http://www.thegef.org/gef/sites/thegef.org/files/documents/c38-inf8-rev1-final.pdf [Accessed September 13, 2010].

S14. Butchart SHM, Bird JP (2010) Data Deficient birds on the IUCN Red List: What don't we know and why does it matter? Biological Conservation 143:239–247.

S15. Reeder DM, Helgen KM, Wilson DE (2007) Global trends and biases in new mammal species discoveries. Occasional Papers, Museum of Texas Tech University 269:1–35.

S16. Rodrigues ASL et al. (2010) A Global Assessment of Amphibian Taxonomic Effort and Expertise. BioScience 60:798–806.
