# Supplementary material for: Spatially Explicit Trends in the Global Conservation Status of Vertebrates
Source: PLoS One. 2014 Nov 26;9(11):e113934. doi: 10.1371/journal.pone.0113934 (PMC4245261; doi:10.1371/journal.pone.0113934)
Supplement: Supporting Information S1 — Supporting Materials and Methods. Figure S1, Variation across hexagons in the weighted change in Red List status per year, for different taxonomic groups. Figure S2, Variation across ecoregions in the weighted change in Red List status per year, for different taxonomic groups. Figure S3, Weighted impact of each threat to the deterioration in global species conservation status, across hexagons. Figure S4, Weighted impact of each threat to the deterioration in global species conservation status, across ecoregions. Figure S5, Relationship between each country's responsibility to conservation and its contribution to changes in the global conservation status of birds, mammals and amphibians. Figure S6, Sensitivity to variation in knowledge of the relationship between each country's responsibility to conservation and its contribution to changes in species global conservation status. Figure S7, Sensitivity of the results to possible spilage across countries. Table S1, Absolute weighted Red List change per country, and list of the species driving those values. Table S2, Main results per country. (ZIP) [file pone.0113934.s001.zip › Rodrigues_etal_Supporting Table S1.pdf]

Table S1: Absolute weighted Red List change per country, and list of the species driving those values. Only species that changed Red List status and that have more than 1% of their range overlapping the country are listed. For each country with at least one such species, the following information is presented: absolute (that is, not divided by the number of years; see equation 1) weighted Red List change (between parentheses); each of the species, followed by the corresponding number of step changes (negative values are deteriorations in Red List status, positive values are improvements), and by the fraction of the species' range in the country. The absolute weighted Red List change per country is the sum across species of the product between the number of step changes and the fraction of the species' range in the country. Data are presented separately for amphibians, birds, and mammals, with the corresponding time interval indicated betweem parentheses in each case.

Amphibians (1980 to 2004)

- **Albania** (-1.76): Rana shqiperica, -2, 0.87.
- **Antigua and Barbuda** (-0.07): Eleutherodactylus martinicensis, -1, 0.07.
- **Argentina** (-5.45): Argenteohyla siemersi, -3, 0.94, Atelognathus patagonicus, -1, 1.00, Atelognathus praebasalticus, -2, 1.00, Bufo rubropunctatus, -1, 0.07, Ceratophrys ornata, -1, 0.94, Leptodactylus laticeps, -1, 0.47, Melanophryniscus devincenzii, -1, 0.08, Pleurodema kriegi, 2, 1.00, Rhinoderma darwinii, -2, 0.03.
- **Australia** (-44.00): Adelotus brevis, -1, 1.00, Crinia tinnula, -1, 1.00, Heleioporus australiacus, -2, 1.00, Litoria aurea, -1, 1.00, Litoria booroolongensis, -2, 1.00, Litoria brevipalmata, -1, 1.00, Litoria cooloolensis, -2, 1.00, Litoria freycineti, -1, 1.00, Litoria lorica, -2, 1.00, Litoria nannotis, -1, 1.00, Litoria nyakalensis, -3, 1.00, Litoria pearsoniana, -1, 1.00, Litoria raniformis, -3, 1.00, Litoria rheocola, -1, 1.00, Litoria spenceri, -1, 1.00, Mixophyes balbus, -2, 1.00, Mixophyes fleayi, -1, 1.00, Mixophyes iteratus, -3, 1.00, Nyctimystes dayi, -1, 1.00, Philoria frosti, -1, 1.00, Philoria sphagnicolus, -1, 1.00, Pseudophryne bibronii, -1, 1.00, Pseudophryne corroboree, -1, 1.00, Pseudophryne pengilleyi, -1, 1.00, Rheobatrachus silus, -1, 1.00, Rheobatrachus vitellinus, -1, 1.00, Taudactylus acutirostris, -3, 1.00, Taudactylus diurnus, 0, 1.00, Taudactylus eungellensis, -1, 1.00, Taudactylus pleione, -2, 1.00, Taudactylus rheophilus, -1, 1.00.
- **Austria** (-0.02): Hyla arborea, -1, 0.02.
- **Belarus** (-0.02): Hyla arborea, -1, 0.02.
- **Belize** (-1.86): Agalychnis moreletii, -4, 0.11, Eleutherodactylus sabrinus, -1, 0.74, Eleutherodactylus sandersoni, -1, 0.66.
- **Bhutan** (-0.08): Occidozyga borealis, -1, 0.08.
- **Bolivia** (-9.49): Atelopus tricolor, -2, 0.69, Gastrotheca splendens, -1, 1.00, Hyla albonigra, -1, 1.00, Leptodactylus laticeps, -1, 0.19, Telmatobius culeus, -2, 0.34, Telmatobius gigas, -2, 1.00, Telmatobius huayra, -1, 1.00, Telmatobius marmoratus, -2, 0.62, Telmatobius simonsi, -1, 1.00.
- **Bosnia and Herzegovina** (-0.01): Hyla arborea, -1, 0.01.
- **Brazil** (-3.41): Atelopus spumarius, -2, 0.34, Ceratophrys ornata, -1, 0.02, Colostethus olfersioides, -2, 1.00, Pleurodema bibroni, -1, 0.70.

- **Brunei Darussalam** (-0.37): Ansonia longidigita, -1, 0.01, Ansonia spinulifer, -1, 0.03, Leptolalax gracilis, -1, 0.02, Limnonectes ingeri, -1, 0.07, Limnonectes paramacrodon, -1, 0.02, Microhyla petrigena, -1, 0.03, Occidozyga baluensis, -1, 0.04, Philautus tectus, -1, 0.01, Rhacophorus dulitensis, -1, 0.06, Staurois tuberilinguis, -1, 0.02.
- **Bulgaria** (-0.03): Hyla arborea, -1, 0.03.
- **Cambodia** (-0.17): Glyphoglossus molossus, -1, 0.17.
- **Cameroon** (-1.58): Conraua goliath, -2, 0.79.
- **Canada** (-0.65): Aneides ferreus, -1, 0.20, Bufo boreas, -1, 0.31, Rana aurora, -1, 0.08, Rana boylii, -1, 0.06.
- **Chile** (-15.88): Alsodes barrioi, -1, 1.00, Alsodes montanus, -2, 1.00, Alsodes tumultuosus, -2, 1.00, Alsodes vanzolinii, -2, 1.00, Bufo rubropunctatus, -1, 0.93, Caudiverbera caudiverbera, -2, 1.00, Rhinoderma darwinii, -2, 0.97, Telmatobius pefauri, -2, 1.00, Telmatobius zapahuirensis, -2, 1.00.
- **China** (-32.68): Amolops loloensis, -1, 1.00, Andrias davidianus, -3, 1.00, Batrachuperus pinchonii, -2, 1.00, Chaparana quadranus, -1, 1.00, Chaparana unculuanus, -2, 0.99, Cynops wolterstorffi, 0, 1.00, Nanorana pleskei, -1, 1.00, Oreolalax lichuanensis, -1, 1.00, Oreolalax rugosus, -1, 1.00, Paa boulengeri, -3, 1.00, Paa exilispinosa, -2, 1.00, Paa jiulongensis, -1, 1.00, Paa maculosa, -1, 1.00, Paa shini, -2, 1.00, Paa spinosa, -2, 0.98, Paa yunnanensis, -3, 1.00, Rana grahami, -1, 1.00, Rana jingdongensis, -2, 0.99, Rana minima, -1, 1.00, Rana nigromaculata, -1, 0.87, Tylototriton asperrimus, -1, 0.89, Tylototriton shanjing, -1, 1.00.
- **Colombia** (-61.60): Atelopus angelito, -2, 1.00, Atelopus arsyecue, -1, 1.00, Atelopus carauta, -1, 1.00, Atelopus carrikeri, -1, 1.00, Atelopus chocoensis, -1, 1.00, Atelopus ebenoides, -1, 1.00, Atelopus eusebianus, -1, 1.00, Atelopus famelicus, -1, 1.00, Atelopus farci, -2, 1.00, Atelopus galactogaster, -2, 1.00, Atelopus glyphus, -2, 0.21, Atelopus guitarraensis, -2, 1.00, Atelopus laetissimus, -1, 1.00, Atelopus longibrachius, -1, 1.00, Atelopus lozanoi, -2, 1.00, Atelopus mandingues, -2, 1.00, Atelopus minutulus, -1, 1.00, Atelopus monohernandezi, -2, 1.00, Atelopus muisca, -3, 1.00, Atelopus nahumae, -1, 1.00, Atelopus nicefori, -2, 1.00, Atelopus pedimarmoratus, -2, 1.00, Atelopus petriruizi, -2, 1.00, Atelopus pictiventris, -1, 1.00, Atelopus quimbaya, -2, 1.00, Atelopus sernai, -1, 1.00, Atelopus simulatus, -1, 1.00, Atelopus sonsonensis, -1, 1.00, Atelopus spumarius, -2, 0.08, Atelopus spurrelli, -2, 1.00, Atelopus subornatus, -1, 1.00, Atelopus tamaense, -2, 0.42, Atelopus walkeri, -1, 1.00, Atopophrynus syntomopus, -1, 1.00, Bolitoglossa capitana, -2, 1.00, Bolitoglossa medemi, -1, 0.96, Bolitoglossa silverstonei, -1, 1.00, Bufo blombergi, -1, 0.74, Centrolene ballux, -2, 0.72, Centrolene heloderma, -3, 0.91, Centrolene lynchi, -1, 0.09, Colostethus lehmanni, -1, 0.89, Colostethus pulchellus, -1, 0.70, Dendrobates lehmanni, -1, 1.00, Dendrobates sylvaticus, -1, 0.48, Gastrotheca angustifrons, -1, 0.97, Hemiphractus bubalus, -1, 0.22, Hemiphractus fasciatus, -1, 0.85, Hyla colymba, -1, 0.10, Osornophryne percrassa, -1, 1.00, Phyllomedusa lemur, -1.
- **Costa Rica** (-41.57): Agalychnis annae, -2, 1.00, Agalychnis saltator, -1, 0.63, Atelopus chiriquiensis, -3, 0.61, Atelopus senex, -2, 1.00, Atelopus varius, -3, 0.68, Bolitoglossa compacta, -2, 0.12, Bolitoglossa lignicolor, -1, 0.69, Bolitoglossa pesrubra, -2, 1.00, Bolitoglossa subpalmata, -2, 1.00, Bufo fastidiosus, -2, 0.64, Bufo holdridgei, -3, 1.00, Bufo periglenes, -3, 1.00, Duellmanohyla uranochroa, -2, 0.81, Eleutherodactylus andi, -2, 1.00, Eleutherodactylus angelicus, -2, 1.00, Eleutherodactylus catalinae, -1, 0.19, Eleutherodactylus escoces, -2, 1.00, Eleutherodactylus fleischmanni, -2, 1.00, Eleutherodactylus ranoides, -4, 0.68, Hyla angustilineata, -1, 0.98, Hyla calypsa, -2, 0.08, Hyla colymba, -1, 0.11, Hyla debilis, -2, 0.10, Hyla rivularis, -3, 0.73, Hyla tica, -3, 0.86, Oedipina gracilis, -1, 0.69, Phyllomedusa lemur, -1, 0.42, Rana vibicaria, -1, 0.73, Rana warszewitschii, -1, 0.24.

- **Croatia** (-0.05): Hyla arborea, -1, 0.01, Rana latastei, -1, 0.04.
- **Cuba** (-2.00): Bufo gundlachi, -1, 1.00, Eleutherodactylus symingtoni, -1, 1.00.
- **Czech Republic** (-0.02): Hyla arborea, -1, 0.02.
- **Dominica** (-1.16): Eleutherodactylus martinicensis, -1, 0.19, Leptodactylus fallax, -1, 0.97.
- **Dominican Republic** (-8.86): Bufo guentheri, -1, 0.65, Eleutherodactylus fowleri, -1, 0.50, Eleutherodactylus furcyensis, -1, 0.12, Eleutherodactylus heminota, -1, 0.18, Eleutherodactylus jugans, -1, 0.15, Eleutherodactylus leoncei, -1, 0.54, Eleutherodactylus nortoni, -1, 0.15, Eleutherodactylus parabates, -1, 0.83, Eleutherodactylus rufifemoralis, -1, 1.00, Eleutherodactylus schmidti, -3, 0.90, Hyla heilprini, -1, 0.52, Osteopilus pulchrilineata, -1, 0.69, Osteopilus vasta, -1, 0.85.
- **Ecuador** (-61.06): Agalychnis litodryas, -2, 0.88, Andinophryne colomai, -2, 1.00, Atelopus arthuri, -2, 1.00, Atelopus balios, -2, 1.00, Atelopus bomolochos, -2, 1.00, Atelopus boulengeri, -1, 1.00, Atelopus coynei, -2, 1.00, Atelopus elegans, -2, 1.00, Atelopus exiguus, -1, 1.00, Atelopus guanujo, -3, 1.00, Atelopus halihelos, -2, 1.00, Atelopus ignescens, -3, 1.00, Atelopus longirostris, -2, 1.00, Atelopus lynchi, -2, 1.00, Atelopus mindoensis, -2, 1.00, Atelopus nanay, -3, 1.00, Atelopus nepiozomus, -1, 1.00, Atelopus pachydermus, -2, 1.00, Atelopus planispina, -2, 1.00, Atelopus spumarius, -2, 0.01, Bufo blombergi, -1, 0.26, Bufo caeruleostictus, -1, 1.00, Centrolene ballux, -2, 0.28, Centrolene heloderma, -3, 0.09, Centrolene lynchi, -1, 0.91, Colostethus anthracinus, -1, 1.00, Colostethus delatorreae, -1, 1.00, Colostethus elachyhistus, -1, 0.56, Colostethus infraguttatus, -1, 1.00, Colostethus lehmanni, -1, 0.11, Colostethus machalilla, -1, 1.00, Colostethus pulchellus, -1, 0.30, Colostethus vertebralis, -3, 1.00, Dendrobates sylvaticus, -1, 0.52, Gastrotheca angustifrons, -1, 0.03, Gastrotheca pseustes, -3, 1.00, Gastrotheca riobambae, -3, 1.00, Hemiphractus bubalus, -1, 0.66, Hemiphractus fasciatus, -1, 0.09, Telmatobius niger, -3, 1.00, Telmatobius vellardi, -2, 1.00.
- **El Salvador** (-0.49): Agalychnis moreletii, -4, 0.11, Plectrohyla guatemalensis, -4, 0.01.
- **Equatorial Guinea** (-0.42): Conraua goliath, -2, 0.21.
- **France** (-4.94): Atelopus flavescens, -2, 1.00, Atelopus franciscus, -2, 1.00, Atelopus spumarius, -2, 0.05, Eleutherodactylus martinicensis, -1, 0.72, Hyla arborea, -1, 0.11.
- **Georgia** (-0.02): Hyla arborea, -1, 0.02.
- **Germany** (-0.09): Hyla arborea, -1, 0.09.
- **Ghana** (-1.14): Afrixalus nigeriensis, -1, 0.25, Amnirana occidentalis, -1, 0.18, Bufo togoensis, -1, 0.21, Leptopelis occidentalis, -1, 0.24, Phrynobatrachus alleni, -1, 0.18, Phrynobatrachus liberiensis, -1, 0.07, Ptychadena superciliaris, -1, 0.02.
- **Greece** (-0.03): Hyla arborea, -1, 0.03.
- **Guatemala** (-13.52): Agalychnis moreletii, -4, 0.62, Dendrotriton cuchumatanus, -2, 1.00, Duellmanohyla soralia, -1, 0.33, Eleutherodactylus greggi, -1, 0.65, Eleutherodactylus lineatus, -2, 0.27, Eleutherodactylus sabrinus, -1, 0.26, Eleutherodactylus sandersoni, -1, 0.34, Hyla bocourti, -2, 1.00, Hyla bromeliacia, -1, 0.91, Plectrohyla glandulosa, -1, 1.00, Plectrohyla guatemalensis, -4, 0.31, Plectrohyla hartwegi, -2, 0.65, Ptychohyla hypomykter, -2, 0.23.
- **Guinea** (-3.36): Afrixalus nigeriensis, -1, 0.02, Amnirana occidentalis, -1, 0.31, Arthroleptis crusculum, -1, 1.00, Bufo togoensis, -1, 0.04, Hyperolius chlorosteus, -1, 0.06, Nimbaphrynoides occidentalis, -2, 0.80, Phrynobatrachus alleni, -1, 0.04, Phrynobatrachus guineensis, -1, 0.12, Phrynobatrachus liberiensis, -1, 0.07, Phrynobatrachus phyllophilus, -1, 0.05, Ptychadena superciliaris, -1, 0.07.
- **Guyana** (-0.33): Atelopus spumarius, -2, 0.16.
- **Haiti** (-18.14): Bufo guentheri, -1, 0.35, Eleutherodactylus amadeus, -1, 1.00, Eleutherodactylus apostates, -1, 1.00, Eleutherodactylus bakeri, -1, 1.00, Eleutherodactylus

brevirostris, -1, 1.00, Eleutherodactylus chlorophenax, -1, 1.00, Eleutherodactylus eunaster, -1, 1.00, Eleutherodactylus fowleri, -1, 0.50, Eleutherodactylus furcyensis, -1, 0.88, Eleutherodactylus glandulifer, -1, 1.00, Eleutherodactylus heminota, -1, 0.82, Eleutherodactylus jugans, -1, 0.85, Eleutherodactylus lamprotes, -1, 1.00, Eleutherodactylus leoncei, -1, 0.46, Eleutherodactylus nortoni, -1, 0.85, Eleutherodactylus oxyrhyncus, -1, 1.00, Eleutherodactylus parabates, -1, 0.17, Eleutherodactylus parapelates, -1, 1.00, Eleutherodactylus paulsoni, -1, 1.00, Eleutherodactylus schmidti, -3, 0.10, Eleutherodactylus warreni, -1, 1.00, Hyla heilprini, -1, 0.48, Osteopilus pulchrilineata, -1, 0.31, Osteopilus vasta, -1, 0.15.

- **Honduras** (-27.29): Agalychnis moreletii, -4, 0.04, Agalychnis saltator, -1, 0.21, Duellmanohyla salvavida, -1, 1.00, Duellmanohyla soralia, -1, 0.67, Eleutherodactylus chrysozetetes, -1, 1.00, Eleutherodactylus emleni, -2, 1.00, Eleutherodactylus epochthidius, -1, 1.00, Eleutherodactylus fecundus, -2, 1.00, Eleutherodactylus laevissimus, -3, 0.77, Eleutherodactylus merendonensis, -3, 1.00, Eleutherodactylus milesi, -2, 1.00, Eleutherodactylus olanchano, -3, 1.00, Eleutherodactylus saltuarius, -2, 1.00, Eleutherodactylus stadelmani, -2, 1.00, Hyla bromeliacia, -1, 0.06, Leptodactylus silvanimbus, -1, 1.00, Plectrohyla dasypus, -1, 1.00, Plectrohyla guatemalensis, -4, 0.63, Ptychohyla hypomykter, -2, 0.16, Rana warszewitschii, -1, 0.04.
- **Hungary** (-0.02): Hyla arborea, -1, 0.02.
- **India** (-0.92): Occidozyga borealis, -1, 0.92.
- **Indonesia** (-14.44): Ansonia albomaculata, -1, 0.43, Ansonia hanitschi, -1, 0.37, Ansonia leptopus, -1, 0.41, Ansonia longidigita, -1, 0.29, Ansonia spinulifer, -1, 0.32, Kalophrynus subterrestris, -1, 0.51, Leptolalax dringi, -1, 0.30, Leptolalax gracilis, -1, 0.49, Leptophryne cruentata, -2, 1.00, Limnonectes blythii, -1, 0.24, Limnonectes ibanorum, -1, 0.43, Limnonectes ingeri, -1, 0.21, Limnonectes magnus, -1, 0.73, Limnonectes malesianus, -1, 0.34, Limnonectes paramacrodon, -1, 0.63, Limnonectes rhacoda, -1, 1.00, Meristogenys phaeomerus, -1, 0.55, Meristogenys poecilus, -1, 0.32, Meristogenys whiteheadi, -1, 0.40, Microhyla perparva, -1, 0.42, Microhyla petrigena, -1, 0.50, Nyctixalus pictus, -1, 0.22, Occidozyga baluensis, -1, 0.04, Pedostibes rugosus, -1, 0.31, Pelophryne signata, -1, 0.28, Philautus hosii, -1, 0.15, Philautus tectus, -1, 0.03, Rhacophorus bifasciatus, -1, 1.00, Rhacophorus dulitensis, -1, 0.07, Rhacophorus kajau, -1, 0.58, Rhacophorus rufipes, -1, 0.59, Staurois tuberilinguis, -1, 0.27.
- **Iran** (-2.00): Batrachuperus gorganensis, -2, 1.00.
- **Italy** (-2.90): Rana latastei, -1, 0.90, Speleomantes supramontis, -2, 1.00.
- **Ivory Coast** (-3.98): Afrixalus nigeriensis, -1, 0.41, Amnirana occidentalis, -1, 0.35, Bufo togoensis, -1, 0.37, Hyperolius chlorosteus, -1, 0.27, Leptopelis occidentalis, -1, 0.64, Nimbaphrynoides occidentalis, -2, 0.20, Phrynobatrachus alleni, -1, 0.39, Phrynobatrachus guineensis, -1, 0.19, Phrynobatrachus liberiensis, -1, 0.42, Phrynobatrachus phyllophilus, -1, 0.15, Ptychadena superciliaris, -1, 0.40.
- **Jamaica** (-3.00): Eleutherodactylus fuscus, -1, 1.00, Eleutherodactylus orcutti, -2, 1.00.
- **Japan** (-0.05): Rana nigromaculata, -1, 0.05.
- **Kenya** (-1.00): Hyperolius cystocandicans, -1, 1.00.
- **Laos** (-0.15): Glyphoglossus molossus, -1, 0.05, Kaloula mediolineata, -1, 0.06, Limnonectes blythii, -1, 0.05.
- **Liberia** (-3.24): Afrixalus nigeriensis, -1, 0.08, Amnirana occidentalis, -1, 0.16, Bufo togoensis, -1, 0.31, Hyperolius chlorosteus, -1, 0.47, Leptopelis occidentalis, -1, 0.12, Phrynobatrachus alleni, -1, 0.28, Phrynobatrachus guineensis, -1, 0.48, Phrynobatrachus liberiensis, -1, 0.35, Phrynobatrachus phyllophilus, -1, 0.65, Ptychadena superciliaris, -1, 0.32.

- **Madagascar** (-4.00): Heterixalus rutenbergi, -1, 1.00, Mantella cowanii, -1, 1.00, Scaphiophryne gottlebei, -1, 1.00, Stumpffia helenae, -1, 1.00.
- **Malaysia** (-21.34): Ansonia albomaculata, -1, 0.56, Ansonia hanitschi, -1, 0.63, Ansonia leptopus, -1, 0.58, Ansonia longidigita, -1, 0.70, Ansonia spinulifer, -1, 0.65, Gastrophrynoides borneensis, -1, 1.00, Kalophrynus subterrestris, -1, 0.49, Leptolalax dringi, -1, 0.70, Leptolalax gracilis, -1, 0.48, Limnonectes blythii, -1, 0.29, Limnonectes ibanorum, -1, 0.56, Limnonectes ingeri, -1, 0.72, Limnonectes malesianus, -1, 0.65, Limnonectes paramacrodon, -1, 0.34, Limnonectes tweediei, -1, 0.99, Meristogenys phaeomerus, -1, 0.45, Meristogenys poecilus, -1, 0.68, Meristogenys whiteheadi, -1, 0.60, Microhyla perparva, -1, 0.58, Microhyla petrigena, -1, 0.43, Nyctixalus pictus, -1, 0.73, Occidozyga baluensis, -1, 0.92, Pedostibes rugosus, -1, 0.68, Pelophryne signata, -1, 0.72, Philautus aurantium, -1, 1.00, Philautus hosii, -1, 0.85, Philautus kerangae, -1, 1.00, Philautus tectus, -1, 0.96, Rhacophorus dulitensis, -1, 0.87, Rhacophorus kajau, -1, 0.42, Rhacophorus rufipes, -1, 0.41, Staurois tuberilinguis, -1, 0.71.
- **Mexico** (-50.23): Agalychnis moreletii, -4, 0.12, Ambystoma andersoni, -2, 1.00, Ambystoma bombypellum, -2, 1.00, Ambystoma dumerilii, -2, 1.00, Ambystoma leorae, -2, 1.00, Ambystoma taylori, -2, 1.00, Bolitoglossa platydactyla, -1, 1.00, Bufo californicus, -2, 0.43, Bufo mexicanus, -1, 1.00, Chiropterotriton cracens, -1, 1.00, Eleutherodactylus alfredi, -1, 0.99, Eleutherodactylus berkenbuschii, -1, 1.00, Eleutherodactylus greggi, -1, 0.35, Eleutherodactylus guerreroensis, -2, 1.00, Eleutherodactylus lineatus, -2, 0.73, Eleutherodactylus polymniae, -2, 1.00, Eleutherodactylus rufescens, -2, 1.00, Hyla altipotens, -2, 1.00, Hyla bromeliacia, -1, 0.02, Hyla dendroscarta, -2, 1.00, Hyla echinata, -2, 1.00, Hyla hazelae, -2, 1.00, Hyla pellita, -2, 1.00, Hyla thorectes, -2, 1.00, Plectrohyla guatemalensis, -4, 0.05, Plectrohyla hartwegi, -2, 0.35, Pseudoeurycea bellii, -2, 1.00, Pseudoeurycea cephalica, -1, 1.00, Pseudoeurycea smithi, -1, 1.00, Rana aurora, -1, 0.04, Rana megapoda, -2, 1.00, Rana omiltemana, -2, 1.00, Rana tarahumarae, -2, 0.98, Spea hammondii, -1, 0.15, Thorius narisovalis, -2, 1.00, Thorius pennatulus, -1, 1.00.
- **Montenegro** (-0.26): Rana shqiperica, -2, 0.13.
- **Morocco** (-1.00): Pelobates varaldii, -1, 1.00.
- **Mozambique** (-0.03): Arthroleptis troglodytes, -2, 0.02.
- **Myanmar** (-0.34): Glyphoglossus molossus, -1, 0.21, Limnonectes blythii, -1, 0.11.
- **Netherlands** (-0.02): Eleutherodactylus martinicensis, -1, 0.01.
- **New Zealand** (-3.00): Leiopelma archeyi, -3, 1.00.
- **Nicaragua** (-3.20): Agalychnis saltator, -1, 0.16, Eleutherodactylus laevissimus, -3, 0.23, Eleutherodactylus ranoides, -4, 0.17, Ptychohyla hypomykter, -2, 0.61, Rana warszewitschii, -1, 0.45.
- **Nigeria** (-0.25): Afrixalus nigeriensis, -1, 0.24.
- **North Korea** (-0.68): Rana chosenica, -1, 0.65, Rana nigromaculata, -1, 0.03.
- **Panama** (-23.69): Agalychnis litodryas, -2, 0.12, Atelopus certus, -1, 1.00, Atelopus chiriquiensis, -3, 0.39, Atelopus glyphus, -2, 0.79, Atelopus limosus, -2, 1.00, Atelopus varius, -3, 0.32, Atelopus zeteki, -1, 1.00, Bolitoglossa compacta, -2, 0.88, Bolitoglossa lignicolor, -1, 0.31, Bolitoglossa medemi, -1, 0.04, Bufo fastidiosus, -2, 0.36, Bufo peripatetes, -1, 1.00, Duellmanohyla uranochroa, -2, 0.19, Eleutherodactylus catalinae, -1, 0.81, Eleutherodactylus emcelae, -1, 1.00, Eleutherodactylus ranoides, -4, 0.15, Hemiphractus fasciatus, -1, 0.05, Hyla angustilineata, -1, 0.02, Hyla calypsa, -2, 0.92, Hyla colymba, -1, 0.80, Hyla debilis, -2, 0.90, Hyla graceae, -2, 1.00, Hyla rivularis, -3, 0.27, Hyla tica, -3, 0.14, Oedipina gracilis, -1, 0.31, Phyllomedusa lemur, -1, 0.57, Rana vibicaria, -1, 0.27, Rana warszewitschii, -1, 0.26.

- **Papua New Guinea** (-4.00): Albericus siegfriedi, -2, 1.00, Austrochaperina novaebritanniae, -1, 1.00, Platymantis akarithyma, -1, 1.00.
- **Paraguay** (-0.34): Leptodactylus laticeps, -1, 0.34.
- **Peru** (-26.67): Atelopus andinus, -3, 1.00, Atelopus erythropus, -2, 1.00, Atelopus peruensis, -3, 1.00, Atelopus pulcher, -1, 1.00, Atelopus reticulatus, -2, 1.00, Atelopus seminiferus, -1, 1.00, Atelopus spumarius, -2, 0.21, Atelopus tricolor, -2, 0.31, Batrachophrynus brachydactylus, -2, 1.00, Batrachophrynus macrostomus, -1, 1.00, Bufo chavin, -1, 1.00, Colostethus elachyhistus, -1, 0.44, Hemiphractus bubalus, -1, 0.12, Phrynopus dagmarae, -1, 1.00, Phrynopus kauneorum, -1, 1.00, Telmatobius arequipensis, -1, 1.00, Telmatobius brevipes, -1, 1.00, Telmatobius carillae, -1, 1.00, Telmatobius colanensis, -1, 1.00, Telmatobius culeus, -2, 0.66, Telmatobius jelskii, -1, 1.00, Telmatobius marmoratus, -2, 0.38.
- **Philippines** (-3.32): Limnonectes macrocephalus, -1, 1.00, Limnonectes magnus, -1, 0.27, Limnonectes visayanus, -1, 1.00, Microhyla petrigena, -1, 0.03, Platymantis hazelae, -1, 1.00.
- **Poland** (-0.08): Hyla arborea, -1, 0.08.
- **Portugal** (-0.91): Alytes cisternasii, -1, 0.27, Hyla arborea, -1, 0.02, Rana iberica, -1, 0.34, Triturus pygmaeus, -1, 0.28.
- **Romania** (-0.03): Hyla arborea, -1, 0.03.
- **Russia** (-0.06): Hyla arborea, -1, 0.04, Rana nigromaculata, -1, 0.03.
- **Sao Tome and Principe** (-2.00): Ptychadena newtoni, -2, 1.00.
- **Serbia** (-0.02): Hyla arborea, -1, 0.02.
- **Sierra Leone** (-1.02): Bufo togoensis, -1, 0.06, Hyperolius chlorosteus, -1, 0.20, Phrynobatrachus alleni, -1, 0.10, Phrynobatrachus guineensis, -1, 0.21, Phrynobatrachus liberiensis, -1, 0.10, Phrynobatrachus phyllophilus, -1, 0.15, Ptychadena superciliaris, -1, 0.19.
- **Slovenia** (-0.06): Rana latastei, -1, 0.05.
- **South Africa** (-6.00): Afrixalus spinifrons, -1, 1.00, Arthroleptella ngongoniensis, -1, 1.00, Hemisus guttatus, -1, 1.00, Microbatrachella capensis, -1, 1.00, Natalobatrachus bonebergi, -1, 1.00, Strongylopus wageri, -1, 1.00.
- **South Korea** (-0.37): Rana chosenica, -1, 0.35, Rana nigromaculata, -1, 0.02.
- **Spain** (-1.19): Alytes cisternasii, -1, 0.73, Alytes dickhilleni, -1, 1.00, Alytes muletensis, 2, 1.00, Hyla arborea, -1, 0.08, Rana iberica, -1, 0.66, Triturus pygmaeus, -1, 0.72.
- **Suriname** (1.71): Atelopus spumarius, -2, 0.14, Dendrobates azureus, 2, 1.00.
- **Tanzania** (-4.00): Bufo brauni, -1, 1.00, Nectophrynoides asperginis, -3, 1.00.
- **Thailand** (-1.67): Glyphoglossus molossus, -1, 0.52, Kaloula mediolineata, -1, 0.86, Leptolalax gracilis, -1, 0.01, Limnonectes blythii, -1, 0.23, Nyctixalus pictus, -1, 0.03.
- **Togo** (-0.01): Bufo togoensis, -1, 0.01.
- **Trinidad and Tobago** (-3.00): Eleutherodactylus urichi, -1, 1.00, Mannophryne olmonae, -2, 1.00.
- **Turkey** (-2.15): Hyla arborea, -1, 0.15, Rana holtzi, -2, 1.00.
- **Ukraine** (-0.11): Hyla arborea, -1, 0.11.
- **United Kingdom** (-0.05): Bufo lemur, -1, 0.02, Leptodactylus fallax, -1, 0.03.
- **United States** (-35.29): Ambystoma barbouri, -1, 1.00, Aneides aeneus, -1, 1.00, Aneides ferreus, -1, 0.80, Bufo baxteri, -1, 1.00, Bufo boreas, -1, 0.68, Bufo californicus, -2, 0.57, Bufo canorus, -1, 1.00, Bufo lemur, -1, 0.98, Eleutherodactylus coqui, -1, 1.00, Eleutherodactylus eneidae, -2, 1.00, Eleutherodactylus gryllus, -2, 1.00, Eleutherodactylus hedricki, -2, 1.00, Eleutherodactylus jasperi, -2, 1.00, Eleutherodactylus locustus, -3, 1.00, Eleutherodactylus portoricensis, -2, 1.00, Eleutherodactylus richmondi, -3, 1.00,

Eleutherodactylus unicolor, -2, 1.00, Eleutherodactylus wightmanae, -2, 1.00, Necturus alabamensis, -1, 1.00, Notophthalmus perstriatus, -1, 1.00, Plethodon neomexicanus, 2, 1.00, Rana areolata, -1, 1.00, Rana aurora, -1, 0.88, Rana boylii, -1, 0.94, Rana capito, -1, 1.00, Rana cascadae, -1, 1.00, Rana muscosa, -1, 1.00, Rana tarahumarae, -2, 0.02, Spea hammondii, -1, 0.85.
- **Uruguay** (-1.42): Argenteohyla siemersi, -3, 0.06, Ceratophrys ornata, -1, 0.04, Melanophryniscus devincenzii, -1, 0.92, Pleurodema bibroni, -1, 0.30.
- **Venezuela** (-30.16): Aromobates nocturnus, -2, 1.00, Atelopus carbonerensis, -3, 1.00, Atelopus chrysocorallus, -3, 1.00, Atelopus cruciger, -2, 1.00, Atelopus mucubajiensis, -3, 1.00, Atelopus oxyrhynchus, -2, 1.00, Atelopus pinangoi, -2, 1.00, Atelopus sorianoi, -2, 1.00, Atelopus tamaense, -2, 0.58, Bolitoglossa spongai, -1, 1.00, Colostethus leopardalis, -2, 1.00, Dendrobates steyermarki, -2, 1.00, Eleutherodactylus boconoensis, -2, 1.00, Eleutherodactylus ginesi, -1, 1.00, Eleutherodactylus lancinii, -1, 1.00, Nephelobates meridensis, -1, 1.00.
- **Viet Nam** (-0.39): Chaparana unculuanus, -2, 0.01, Glyphoglossus molossus, -1, 0.05, Kaloula mediolineata, -1, 0.09, Limnonectes blythii, -1, 0.08, Paa spinosa, -2, 0.02, Tylototriton asperrimus, -1, 0.11.
- **Zimbabwe** (-1.97): Arthroleptis troglodytes, -2, 0.98.

Birds (1988 to 2008)

- **Afghanistan** (-0.23): Chlamydotis undulata, -2, 0.03, Columba eversmanni, -1, 0.02, Coracias garrulus, -1, 0.03, Falco cherrug, -2, 0.02, Neophron percnopterus, -3, 0.03.
- **Algeria** (-0.54): Chlamydotis undulata, -2, 0.08, Neophron percnopterus, -3, 0.04, Puffinus mauretanicus, -2, 0.03, Sylvia undata, -1, 0.19.
- **Angola** (-0.25): Coracias garrulus, -1, 0.05, Neotis denhami, -1, 0.04, Rynchops flavirostris, -1, 0.12.
- **Argentina** (-2.42): Diomedea dabbenena, -1, 0.01, Phalacrocorax bougainvillii, -1, 0.08, Phoebetria fusca, -1, 0.02, Phoenicoparrus jamesi, 1, 0.28, Phoenicopterus chilensis, -1, 0.70, Pipile jacutinga, -1, 0.03, Podiceps gallardoi, -2, 0.87, Procnias nudicollis, -1, 0.02, Thalassarche melanophrys, -2, 0.05.
- **Armenia** (-0.10): Tetrao mlokosiewiczi, -1, 0.10.
- **Australia** (-3.12): Atrichornis rufescens, -1, 1.00, Cyanoramphus cookii, 1, 1.00, Dasyornis longirostris, 1, 1.00, Grantiella picta, -1, 1.00, Lathamus discolor, -1, 1.00, Limosa limosa, -1, 0.04, Menura alberti, 1, 1.00, Neophema chrysogaster, -1, 1.00, Phoebetria fusca, -1, 0.33, Pterodroma cookii, 1, 0.05, Sterna nereis, -1, 1.00, Stipiturus mallee, -1, 1.00, Sypheotides indicus, 1, 0.66, Thalassarche melanophrys, -2, 0.23.
- **Azerbaijan** (-0.09): Haliaeetus albicilla, 1, 0.01, Tetrao mlokosiewiczi, -1, 0.07.
- **Bangladesh** (-0.48): Aythya baeri, -1, 0.02, Eurynorhynchus pygmeus, -2, 0.05, Gyps bengalensis, -3, 0.03, Gyps tenuirostris, -3, 0.03, Houbaropsis bengalensis, -1, 0.17.
- **Belarus** (-0.04): Haliaeetus albicilla, 1, 0.04, Milvus milvus, -1, 0.05, Numenius arquata, -1, 0.01.
- **Benin** (-0.04): Balearica pavonina, -2, 0.01, Neotis denhami, -1, 0.01.
- **Bhutan** (-0.09): Argusianus argus, -1, 0.02, Prinia cinereocapilla, -1, 0.07.
- **Bolivia** (-3.90): Anodorhynchus hyacinthinus, -1, 0.05, Ara glaucogularis, -1, 1.00, Phoenicoparrus jamesi, 1, 0.41, Phoenicopterus chilensis, -1, 0.09, Picumnus fuscus, -1, 0.59, Rollandia microptera, -3, 0.85.
- **Botswana** (-0.13): Coracias garrulus, -1, 0.02, Limosa limosa, -1, 0.01, Rynchops flavirostris, -1, 0.02, Torgos tracheliotos, -1, 0.06.

- **Brazil** (-5.52): Amazona brasiliensis, 1, 1.00, Anodorhynchus hyacinthinus, -1, 0.92, Anodorhynchus leari, 1, 1.00, Cercomacra ferdinandi, -2, 1.00, Chaetura pelagica, -1, 0.14, Crax blumenbachii, 1, 1.00, Cyanopsitta spixii, -1, 1.00, Diomedea dabbenena, -1, 0.16, Penelope jacucaca, -1, 1.00, Phoebetria fusca, -1, 0.04, Picumnus fuscus, -1, 0.41, Pipile jacutinga, -1, 0.90, Procnias nudicollis, -1, 0.91, Suiriri islerorum, -1, 0.99, Thalassarche melanophrys, -2, 0.02.
- **Brunei Darussalam** (-0.39): Alcippe brunneicauda, -1, 0.01, Anthreptes rhodolaemus, -1, 0.01, Harpactes orrhophaeus, -1, 0.01, Kenopia striata, -1, 0.02, Stachyris leucotis, -1, 0.01, Stachyris nigricollis, -1, 0.02, Turdinus atrigularis, -1, 0.02.
- **Bulgaria** (-0.03): Branta ruficollis, -1, 0.01.
- **Burkina Faso** (-0.17): Balearica pavonina, -2, 0.02, Neophron percnopterus, -3, 0.01, Neotis denhami, -1, 0.04, Rynchops flavirostris, -1, 0.03.
- **Burundi** (-0.12): Bradypterus graueri, -1, 0.11.
- **Cambodia** (-1.25): Cuculus vagans, -1, 0.03, Gyps bengalensis, -3, 0.04, Gyps tenuirostris, -3, 0.16, Houbaropsis bengalensis, -1, 0.08, Pseudibis davisoni, -1, 0.39, Sarcogyps calvus, -3, 0.04.
- **Cameroon** (-1.06): Francolinus camerunensis, -1, 1.00, Neotis denhami, -1, 0.02, Rynchops flavirostris, -1, 0.01.
- **Canada** (-0.35): Chaetura pelagica, -1, 0.10, Phoebastria nigripes, -3, 0.03, Tympanuchus cupido, -1, 0.17.
- **Central African Republic** (-0.16): Balearica pavonina, -2, 0.02, Neotis denhami, -1, 0.08, Rynchops flavirostris, -1, 0.01.
- **Chad** (-0.55): Balearica pavonina, -2, 0.11, Falco cherrug, -2, 0.02, Limosa limosa, -1, 0.03, Neophron percnopterus, -3, 0.05, Neotis denhami, -1, 0.06, Rynchops flavirostris, -1, 0.01, Torgos tracheliotos, -1, 0.04.
- **Chile** (-1.36): Phalacrocorax bougainvillii, -1, 0.52, Phoenicoparrus jamesi, 1, 0.21, Phoenicopterus chilensis, -1, 0.05, Podiceps gallardoi, -2, 0.13, Pterodroma cookii, 1, 0.02, Spheniscus humboldti, -1, 0.47, Thalassarche melanophrys, -2, 0.14.
- **China** (-2.62): Argusianus argus, -1, 0.10, Aythya baeri, -1, 0.65, Chlamydotis undulata, -2, 0.07, Columba eversmanni, -1, 0.23, Coracias garrulus, -1, 0.02, Emberiza aureola, -1, 0.07, Emberiza jankowskii, -1, 0.87, Falco cherrug, -2, 0.23, Gorsachius goisagi, -1, 0.32, Grus monacha, -1, 0.07, Haliaeetus albicilla, 1, 0.09, Larus relictus, -1, 0.13, Limosa limosa, -1, 0.02, Nipponia nippon, 1, 1.00, Numenius arquata, -1, 0.05, Platalea minor, 1, 0.54, Pycnonotus taivanus, -1, 1.00, Sarcogyps calvus, -3, 0.03.
- **Colombia** (-3.86): Chaetura pelagica, -1, 0.10, Lepidopyga lilliae, -1, 1.00, Metallura iracunda, -1, 0.51, Mitu mitu, -1, 1.00, Ognorhynchus icterotis, 1, 0.96, Pauxi pauxi, -1, 0.12, Penelope ortoni, -1, 0.53, Schizoeaca perijana, -1, 0.56, Synallaxis fuscorufa, -1, 1.00.
- **Cook Islands** (0.96): Pomarea dimidiata, 1, 1.00, Pterodroma alba, -1, 0.14, Pterodroma cookii, 1, 0.09.
- **Croatia** (-0.03): Puffinus mauretanicus, -2, 0.01.
- **Cuba** (0.06): Patagioenas inornata, 1, 0.19, Vermivora bachmanii, -1, 0.12.
- **Czech Republic** (-0.01): Milvus milvus, -1, 0.01.
- **Democratic Republic of the Congo** (-1.21): Ardeola idae, -1, 0.17, Bradypterus graueri, -1, 0.64, Coracias garrulus, -1, 0.02, Limosa limosa, -1, 0.03, Neotis denhami, -1, 0.10, Rynchops flavirostris, -1, 0.23.
- **Denmark** (-0.04): Milvus milvus, -1, 0.01, Puffinus mauretanicus, -2, 0.01.
- **Djibouti** (-1.01): Francolinus ochropectus, -1, 1.00.
- **Dominica** (-0.32): Cichlherminia lherminieri, -1, 0.32.
- **Dominican Republic** (-0.41): Buteo ridgwayi, -1, 0.73, Patagioenas inornata, 1, 0.33.

- **East Timor** (-1.08): Cacatua sulphurea, -1, 0.06, Gallicolumba hoedtii, -1, 0.44, Treron psittaceus, -1, 0.52, Zoothera dohertyi, -1, 0.06.
- **Ecuador** (-4.06): Brotogeris pyrrhoptera, -1, 0.82, Chaetura pelagica, -1, 0.02, Hapalopsittaca pyrrhops, -1, 0.99, Mimus trifasciatus, -1, 1.00, Ognorhynchus icterotis, 1, 0.04, Penelope ortoni, -1, 0.47, Phoebastria irrorata, -2, 0.39.
- **Egypt** (-0.67): Chlamydotis undulata, -2, 0.05, Coracias garrulus, -1, 0.03, Falco cherrug, -2, 0.03, Puffinus mauretanicus, -2, 0.02, Serinus syriacus, -1, 0.41.
- **Eritrea** (-0.12): Balearica pavonina, -2, 0.01, Torgos tracheliotos, -1, 0.01, Vanellus gregarius, -1, 0.03.
- **Ethiopia** (-3.24): Acrocephalus griseldis, -2, 0.20, Balearica pavonina, -2, 0.19, Coracias garrulus, -1, 0.03, Falco cherrug, -2, 0.03, Heteromirafra sidamoensis, -1, 1.00, Limosa limosa, -1, 0.04, Neophron percnopterus, -3, 0.06, Neotis denhami, -1, 0.01, Rynchops flavirostris, -1, 0.02, Torgos tracheliotos, -1, 0.12, Zavattariornis stresemanni, -1, 1.00.
- **Fiji** (-0.07): Pterodroma alba, -1, 0.07.
- **Finland** (-0.02): Numenius arquata, -1, 0.02.
- **France** (-7.47): Anas eatoni, -1, 1.00, Cichlherminia lherminieri, -1, 0.47, Coracina newtoni, -1, 1.00, Ducula galeata, 1, 1.00, Haliaeetus albicilla, 1, 0.02, Milvus milvus, -1, 0.16, Numenius arquata, -1, 0.01, Phoebetria fusca, -1, 0.28, Pomarea whitneyi, -2, 1.00, Pterodroma alba, -1, 0.32, Pterodroma cookii, 1, 0.22, Puffinus mauretanicus, -2, 0.08, Sitta whiteheadi, -2, 1.00, Sylvia undata, -1, 0.13, Thalassarche melanophrys, -2, 0.08, Todiramphus godeffroyi, -1, 1.00.
- **Georgia** (-0.38): Haliaeetus albicilla, 1, 0.01, Sitta krueperi, -1, 0.04, Tetrao mlokosiewiczi, -1, 0.33.
- **Germany** (-0.17): Haliaeetus albicilla, 1, 0.02, Milvus milvus, -1, 0.16.
- **Ghana** (-0.04): Neotis denhami, -1, 0.02, Rynchops flavirostris, -1, 0.01.
- **Greece** (-0.10): Puffinus mauretanicus, -2, 0.04.
- **Guinea** (-0.06): Neotis denhami, -1, 0.03.
- **Haiti** (-0.20): Buteo ridgwayi, -1, 0.27, Patagioenas inornata, 1, 0.07.
- **India** (-10.66): Argusianus argus, -1, 0.08, Aythya baeri, -1, 0.04, Chlamydotis undulata, -2, 0.03, Columba eversmanni, -1, 0.19, Coracias garrulus, -1, 0.03, Emberiza aureola, -1, 0.02, Eurynorhynchus pygmeus, -2, 0.07, Gyps bengalensis, -3, 0.57, Gyps indicus, -3, 0.96, Gyps tenuirostris, -3, 0.46, Haliaeetus albicilla, 1, 0.05, Houbaropsis bengalensis, -1, 0.62, Limosa limosa, -1, 0.07, Neophron percnopterus, -3, 0.15, Numenius arquata, -1, 0.02, Oxyura leucocephala, -1, 0.01, Prinia cinereocapilla, -1, 0.12, Pycnonotus xantholaemus, -1, 1.00, Sarcogyps calvus, -3, 0.68, Sypheotides indicus, 1, 0.33, Vanellus gregarius, -1, 0.17.
- **Indonesia** (-52.86): Aceros comatus, -1, 0.64, Actenoides concretus, -1, 0.70, Aegithina viridissima, -1, 0.70, Alcedo euryzona, -1, 0.73, Alcippe brunneicauda, -1, 0.56, Alophoixus finschii, -1, 0.75, Anthreptes rhodolaemus, -1, 0.43, Argusianus argus, -1, 0.51, Batrachostomus stellatus, -1, 0.74, Buceros rhinoceros, -1, 0.75, Cacatua sulphurea, -1, 0.94, Caloperdix oculeus, -1, 0.59, Calyptomena hosii, -1, 0.63, Calyptomena viridis, -1, 0.68, Caprimulgus concretus, -1, 0.85, Centropus rectunguis, -1, 0.76, Chloropsis cyanopogon, -1, 0.71, Cuculus vagans, -1, 0.60, Cyornis caerulatus, -1, 0.85, Dinopium rafflesii, -1, 0.66, Enicurus ruficapillus, -1, 0.67, Eupetes macrocerus, -1, 0.68, Eurylaimus ochromalus, -1, 0.65, Eurystomus azureus, -1, 1.00, Ficedula dumetoria, -1, 0.67, Gallicolumba hoedtii, -1, 0.56, Harpactes diardii, -1, 0.68, Harpactes duvaucelii, -1, 0.67, Harpactes kasumba, -1, 0.67, Harpactes orrhophaeus, -1, 0.61, Iole olivacea, -1, 0.69, Ixos malaccensis, -1, 0.68, Kenopia striata, -1, 0.48, Limosa limosa, -1, 0.06, Macronous ptilosus, -1, 0.70, Malacocincla malaccensis, -1, 0.72, Malacopteron affine, -1, 0.65, Malacopteron magnum, -1, 0.77, Megalaima henricii, -1, 0.74, Megalaima mystacophanos,

-1, 0.68, Meiglyptes tukki, -1, 0.70, Melanoperdix niger, -1, 0.77, Numenius arquata, -1, 0.03, Oriolus xanthonotus, -1, 0.68, Otus rufescens, -1, 0.75, Padda oryzivora, -1, 1.00, Pericrocotus igneus, -1, 0.69, Phaenicophaeus diardi, -1, 0.69, Phaenicophaeus sumatranus, -1, 0.70, Philentoma velata, -1, 0.68, Pitta baudii, -1, 0.76, Pitta granatina, -1, 0.76, Platylophus galericulatus, -1, 0.74, Prionochilus thoracicus, -1, 0.72, Pseudibis davisoni, -1, 0.42, Psittacula longicauda, -1, 0.77, Ptilinopus jambu, -1, 0.36, Ptilocichla leucogrammica, -1, 0.76, Pycnonotus cyaniventris, -1, 0.69, Pycnonotus eutilotus, -1, 0.70, Pycnonotus melanoleucos, -1, 0.72, Pycnonotus squamatus, -1, 0.70, Rhinomyias umbratilis, -1, 0.72, Rhizothera longirostris, -1, 0.65, Rollulus rouloul, -1, 0.70, Setornis criniger, -1, 0.81, Stachyris leucotis, -1, 0.25, Stachyris maculata, -1, 0.70, Stachyris nigricollis, -1, 0.34, Sturnus melanopterus, -2, 1.00, Todiramphus funebris, -1, 1.00, Treron capellei, -1, 0.74, Treron floris, -1, 1.00, Treron psittaceus, -1, 0.48, Trichixos pyrropygus, -1, 0.71, Turdinus atrigularis, -1, 0.19, Zoothera dohertyi, -1, 0.94

- **Iran** (-0.72): Chlamydotis undulata, -2, 0.11, Columba eversmanni, -1, 0.04, Coracias garrulus, -1, 0.05, Falco cherrug, -2, 0.05, Haliaeetus albicilla, 1, 0.02, Neophron percnopterus, -3, 0.08, Oxyura leucocephala, -1, 0.05, Tetrao mlokosiewiczi, -1, 0.02.
- **Iraq** (-0.49): Acrocephalus griseldis, -2, 0.16, Chlamydotis undulata, -2, 0.03, Falco cherrug, -2, 0.01, Haliaeetus albicilla, 1, 0.01, Serinus syriacus, -1, 0.06, Vanellus gregarius, -1, 0.02.
- **Ireland** (-0.18): Puffinus mauretanicus, -2, 0.09.
- **Israel** (-0.21): Serinus syriacus, -1, 0.19.
- **Italy** (-0.38): Milvus milvus, -1, 0.07, Puffinus mauretanicus, -2, 0.13, Sylvia undata, -1, 0.04.
- **Ivory Coast** (-0.04): Neotis denhami, -1, 0.02.
- **Jamaica** (0.22): Patagioenas inornata, 1, 0.22.
- **Japan** (-0.99): Aythya baeri, -1, 0.05, Gorsachius goisagi, -1, 0.32, Haliaeetus albicilla, 1, 0.04, Phoebastria nigripes, -3, 0.29, Platalea minor, 1, 0.24, Polysticta stelleri, -1, 0.02.
- **Jordan** (-0.14): Serinus syriacus, -1, 0.12.
- **Kazakhstan** (-1.64): Chlamydotis undulata, -2, 0.06, Columba eversmanni, -1, 0.17, Coracias garrulus, -1, 0.09, Emberiza aureola, -1, 0.03, Falco cherrug, -2, 0.08, Haliaeetus albicilla, 1, 0.03, Larus relictus, -1, 0.11, Limosa limosa, -1, 0.06, Neophron percnopterus, -3, 0.03, Numenius arquata, -1, 0.08, Oxyura leucocephala, -1, 0.34, Vanellus gregarius, -1, 0.41.
- **Kenya** (-1.35): Acrocephalus griseldis, -2, 0.28, Anthus sokokensis, -1, 0.47, Ardeola idae, -1, 0.07, Coracias garrulus, -1, 0.02, Limosa limosa, -1, 0.02, Neophron percnopterus, -3, 0.03, Neotis denhami, -1, 0.01, Rynchops flavirostris, -1, 0.02, Torgos tracheliotos, -1, 0.06.
- **Kiribati** (-0.06): Pterodroma alba, -1, 0.18, Pterodroma cookii, 1, 0.12.
- **Kyrgyzstan** (-0.12): Columba eversmanni, -1, 0.04, Falco cherrug, -2, 0.01, Neophron percnopterus, -3, 0.01.
- **Laos** (-0.52): Cuculus vagans, -1, 0.02, Emberiza aureola, -1, 0.01, Gyps bengalensis, -3, 0.03, Gyps tenuirostris, -3, 0.07, Pseudibis davisoni, -1, 0.06, Sarcogyps calvus, -3, 0.04.
- **Lebanon** (-0.21): Serinus syriacus, -1, 0.20.
- **Libya** (-0.37): Chlamydotis undulata, -2, 0.07, Falco cherrug, -2, 0.02, Puffinus mauretanicus, -2, 0.08.
- **Lithuania** (-0.01): Haliaeetus albicilla, 1, 0.01.
- **Madagascar** (-2.17): Anas melleri, -1, 1.00, Ardeola idae, -1, 0.14, Numenius arquata, -1, 0.02, Tachybaptus rufolavatus, -1, 1.00.
- **Malawi** (-0.70): Acrocephalus griseldis, -2, 0.02, Alethe choloensis, -1, 0.61, Ardeola idae, -1, 0.03.

- **Malaysia** (-17.91): Aceros comatus, -1, 0.24, Actenoides concretus, -1, 0.24, Aegithina viridissima, -1, 0.26, Alcedo euryzona, -1, 0.21, Alcippe brunneicauda, -1, 0.42, Alophoixus finschii, -1, 0.24, Anthreptes rhodolaemus, -1, 0.42, Argusianus argus, -1, 0.16, Batrachostomus stellatus, -1, 0.25, Buceros rhinoceros, -1, 0.22, Caloperdix oculeus, -1, 0.15, Calyptomena hosii, -1, 0.37, Calyptomena viridis, -1, 0.23, Caprimulgus concretus, -1, 0.15, Centropus rectunguis, -1, 0.24, Chloropsis cyanopogon, -1, 0.23, Cuculus vagans, -1, 0.24, Cyornis caerulatus, -1, 0.15, Dinopium rafflesii, -1, 0.25, Enicurus ruficapillus, -1, 0.26, Eupetes macrocerus, -1, 0.26, Eurylaimus ochromalus, -1, 0.22, Ficedula dumetoria, -1, 0.30, Gyps bengalensis, -3, 0.02, Harpactes diardii, -1, 0.26, Harpactes duvaucelii, -1, 0.24, Harpactes kasumba, -1, 0.29, Harpactes orrhophaeus, -1, 0.35, Iole olivacea, -1, 0.26, Ixos malaccensis, -1, 0.26, Kenopia striata, -1, 0.45, Limosa limosa, -1, 0.01, Macronous ptilosus, -1, 0.27, Malacocincla malaccensis, -1, 0.26, Malacopteron affine, -1, 0.31, Malacopteron magnum, -1, 0.20, Megalaima henricii, -1, 0.23, Megalaima mystacophanos, -1, 0.23, Meiglyptes tukki, -1, 0.22, Melanoperdix niger, -1, 0.23, Numenius arquata, -1, 0.01, Oriolus xanthonotus, -1, 0.25, Otus rufescens, -1, 0.23, Pericrocotus igneus, -1, 0.22, Phaenicophaeus diardi, -1, 0.24, Phaenicophaeus sumatranus, -1, 0.23, Philentoma velata, -1, 0.25, Pitta baudii, -1, 0.24, Pitta granatina, -1, 0.24, Platylophus galericulatus, -1, 0.21, Prionochilus thoracicus, -1, 0.27, Psittacula longicauda, -1, 0.21, Ptilinopus jambu, -1, 0.63, Ptilocichla leucogrammica, -1, 0.24, Pycnonotus cyaniventris, -1, 0.23, Pycnonotus eutilotus, -1, 0.21, Pycnonotus melanoleucos, -1, 0.26, Pycnonotus squamatus, -1, 0.26, Rhinomyias umbratilis, -1, 0.27, Rhizothera longirostris, -1, 0.24, Rollulus rouloul, -1, 0.23, Setornis criniger, -1, 0.18, Stachyris leucotis, -1, 0.64, Stachyris maculata, -1, 0.27, Stachyris nigricollis, -1, 0.49, Treron capellei, -1, 0.22, Trichixos pyrropygus, -1, 0.27, Turdinus atrigularis, -1, 0.79.
- **Mali** (-0.39): Balearica pavonina, -2, 0.05, Limosa limosa, -1, 0.01, Neophron percnopterus, -3, 0.06, Neotis denhami, -1, 0.06, Rynchops flavirostris, -1, 0.03, Torgos tracheliotos, -1, 0.01.
- **Malta** (-0.03): Puffinus mauretanicus, -2, 0.01.
- **Mauritania** (-0.12): Chlamydotis undulata, -2, 0.02, Neophron percnopterus, -3, 0.02.
- **Mauritius** (8.00): Acrocephalus rodericanus, 1, 1.00, Falco punctatus, 2, 1.00, Foudia flavicans, 1, 1.00, Foudia rubra, 2, 1.00, Nesoenas mayeri, 1, 1.00, Psittacula eques, 1, 1.00.
- **Mexico** (-1.41): Chaetura pelagica, -1, 0.03, Phoebastria nigripes, -3, 0.03, Pterodroma cookii, 1, 0.03, Puffinus opisthomelas, 1, 0.68, Toxostoma guttatum, -2, 1.00.
- **Moldova** (-0.02): Milvus milvus, -1, 0.02.
- **Mongolia** (-1.15): Chlamydotis undulata, -2, 0.08, Emberiza aureola, -1, 0.03, Falco cherrug, -2, 0.09, Larus relictus, -1, 0.72, Numenius arquata, -1, 0.02, Oxyura leucocephala, -1, 0.02.
- **Morocco** (-0.44): Chlamydotis undulata, -2, 0.03, Milvus milvus, -1, 0.02, Neophron percnopterus, -3, 0.02, Puffinus mauretanicus, -2, 0.03, Sylvia undata, -1, 0.21, Torgos tracheliotos, -1, 0.01.
- **Mozambique** (-1.03): Acrocephalus griseldis, -2, 0.02, Alethe choloensis, -1, 0.39, Ardeola idae, -1, 0.15, Coracias garrulus, -1, 0.03, Limosa limosa, -1, 0.03, Rynchops flavirostris, -1, 0.08, Spheniscus demersus, -1, 0.28, Torgos tracheliotos, -1, 0.01.
- **Myanmar** (-2.29): Aceros comatus, -1, 0.03, Actenoides concretus, -1, 0.02, Aegithina viridissima, -1, 0.01, Alcedo euryzona, -1, 0.02, Anthreptes rhodolaemus, -1, 0.06, Argusianus argus, -1, 0.04, Aythya baeri, -1, 0.03, Caloperdix oculeus, -1, 0.10, Calyptomena viridis, -1, 0.03, Cuculus vagans, -1, 0.02, Dinopium rafflesii, -1, 0.05, Emberiza aureola, -1, 0.04, Enicurus ruficapillus, -1, 0.02, Eurylaimus ochromalus, -1, 0.04, Gyps bengalensis, -3, 0.10, Gyps tenuirostris, -3, 0.25, Harpactes duvaucelii, -1, 0.02, Iole

olivacea, -1, 0.02, Ixos malaccensis, -1, 0.03, Macronous ptilosus, -1, 0.01, Malacopteron magnum, -1, 0.01, Megalaima mystacophanos, -1, 0.02, Meiglyptes tukki, -1, 0.03, Oriolus xanthonotus, -1, 0.03, Philentoma velata, -1, 0.03, Pycnonotus cyaniventris, -1, 0.04, Pycnonotus eutilotus, -1, 0.04, Pycnonotus squamatus, -1, 0.01, Rhizothera longirostris, -1, 0.02, Rollulus rouloul, -1, 0.01, Sarcogyps calvus, -3, 0.12.

- **Namibia** (-1.10): Coracias garrulus, -1, 0.01, Diomedea dabbenena, -1, 0.18, Phalacrocorax neglectus, -1, 0.50, Spheniscus demersus, -1, 0.25, Thalassarche melanophrys, -2, 0.02, Torgos tracheliotos, -1, 0.08.
- **Nepal** (-1.10): Argusianus argus, -1, 0.05, Gyps tenuirostris, -3, 0.03, Haliaeetus albicilla, 1, 0.02, Houbaropsis bengalensis, -1, 0.08, Prinia cinereocapilla, -1, 0.81, Sarcogyps calvus, -3, 0.01.
- **Netherlands** (-0.03): Puffinus mauretanicus, -2, 0.02.
- **New Zealand** (-3.24): Apteryx owenii, 1, 1.00, Cyanoramphus malherbi, -1, 1.00, Larus bulleri, -1, 1.00, Mohoua ochrocephala, -1, 1.00, Phalacrocorax featherstoni, -1, 1.00, Pterodroma alba, -1, 0.04, Pterodroma axillaris, 1, 1.00, Pterodroma cookii, 1, 0.19, Sterna albostriata, -1, 1.00, Thalassarche melanophrys, -2, 0.19.
- **Niger** (-0.36): Balearica pavonina, -2, 0.05, Limosa limosa, -1, 0.02, Neophron percnopterus, -3, 0.05, Neotis denhami, -1, 0.02, Torgos tracheliotos, -1, 0.04.
- **Nigeria** (-0.20): Balearica pavonina, -2, 0.02, Limosa limosa, -1, 0.02, Neotis denhami, -1, 0.08, Rynchops flavirostris, -1, 0.04, Torgos tracheliotos, -1, 0.01.
- **Niue** (-0.01): Pterodroma alba, -1, 0.02, Pterodroma cookii, 1, 0.02.
- **North Korea** (0.09): Emberiza jankowskii, -1, 0.02, Platalea minor, 1, 0.11.
- **Norway** (-0.11): Haliaeetus albicilla, 1, 0.02, Numenius arquata, -1, 0.01, Polysticta stelleri, -1, 0.02, Puffinus mauretanicus, -2, 0.03, Thalassarche melanophrys, -2, 0.02.
- **Oman** (-0.08): Chlamydotis undulata, -2, 0.02.
- **Pakistan** (-0.71): Chlamydotis undulata, -2, 0.04, Columba eversmanni, -1, 0.12, Coracias garrulus, -1, 0.03, Gyps bengalensis, -3, 0.07, Gyps indicus, -3, 0.04, Haliaeetus albicilla, 1, 0.02, Neophron percnopterus, -3, 0.04, Oxyura leucocephala, -1, 0.01.
- **Papua New Guinea** (-9.57): Accipiter princeps, -1, 1.00, Cacatua ophthalmica, -1, 1.00, Centropus violaceus, -1, 1.00, Ducula finschii, -1, 1.00, Ducula rubricera, -1, 0.64, Megalurulus grosvenori, -1, 1.00, Melidectes whitemanensis, -1, 1.00, Nesoclopeus woodfordi, 1, 0.37, Ninox odiosa, -1, 1.00, Reinwardtoena browni, -1, 1.00, Reinwardtoena crassirostris, -1, 0.30, Tyto aurantia, -1, 1.00.
- **Paraguay** (-0.20): Anodorhynchus hyacinthinus, -1, 0.03, Phoenicopterus chilensis, -1, 0.03, Pipile jacutinga, -1, 0.07, Procnias nudicollis, -1, 0.07.
- **Peru** (-3.93): Brotogeris pyrrhoptera, -1, 0.18, Chaetura pelagica, -1, 0.11, Forpus xanthops, -1, 1.00, Phalacrocorax bougainvillii, -1, 0.39, Phoebastria irrorata, -2, 0.61, Phoenicoparrus jamesi, 1, 0.10, Phoenicopterus chilensis, -1, 0.11, Rollandia microptera, -3, 0.15, Spheniscus humboldti, -1, 0.53, Thalassarche melanophrys, -2, 0.03.
- **Philippines** (-3.43): Anas luzonica, -1, 1.00, Anthracoceros montani, -1, 1.00, Cacatua haematuropygia, -1, 1.00, Gorsachius goisagi, -1, 0.36, Numenius arquata, -1, 0.01, Oriolus xanthonotus, -1, 0.01, Pericrocotus igneus, -1, 0.01.
- **Poland** (-0.07): Haliaeetus albicilla, 1, 0.03, Limosa limosa, -1, 0.01, Milvus milvus, -1, 0.09.
- **Portugal** (0.70): Columba trocaz, 1, 1.00, Milvus milvus, -1, 0.04, Puffinus mauretanicus, -2, 0.10, Sylvia undata, -1, 0.06.
- **Romania** (-0.04): Branta ruficollis, -1, 0.02.
- **Russia** (-8.00): Aythya baeri, -1, 0.19, Brachyramphus brevirostris, -4, 0.24, Branta ruficollis, -1, 0.95, Coracias garrulus, -1, 0.09, Emberiza aureola, -1, 0.72, Emberiza

jankowskii, -1, 0.11, Eurynorhynchus pygmeus, -2, 0.85, Falco cherrug, -2, 0.18, Grus monacha, -1, 0.93, Haliaeetus albicilla, 1, 0.36, Larus relictus, -1, 0.01, Limosa limosa, -1, 0.17, Numenius arquata, -1, 0.41, Oxyura leucocephala, -1, 0.22, Phoebastria nigripes, -3, 0.08, Polysticta stelleri, -1, 0.71, Sitta krueperi, -1, 0.09, Tetrao mlokosiewiczi, -1, 0.37, Vanellus gregarius, -1, 0.09.

- **Rwanda** (-0.15): Bradypterus graueri, -1, 0.14.
- **Saint Lucia** (-0.16): Cichlherminia lherminieri, -1, 0.16.
- **Samoa** (-2.00): Myiagra albiventris, -2, 1.00.
- **Saudi Arabia** (-0.44): Chlamydotis undulata, -2, 0.14, Neophron percnopterus, -3, 0.03, Numenius arquata, -1, 0.01, Torgos tracheliotos, -1, 0.05.
- **Senegal** (-0.11): Balearica pavonina, -2, 0.02, Neophron percnopterus, -3, 0.01, Rynchops flavirostris, -1, 0.01, Torgos tracheliotos, -1, 0.01.
- **Seychelles** (2.00): Copsychus sechellarum, 1, 1.00, Foudia sechellarum, 1, 1.00.
- **Solomon Islands** (-0.44): Ducula rubricera, -1, 0.36, Nesoclopeus woodfordi, 1, 0.63, Reinwardtoena crassirostris, -1, 0.70.
- **Somalia** (-0.12): Acrocephalus griseldis, -2, 0.03, Limosa limosa, -1, 0.01, Numenius arquata, -1, 0.01, Torgos tracheliotos, -1, 0.01.
- **South Africa** (-2.85): Acrocephalus griseldis, -2, 0.02, Coracias garrulus, -1, 0.04, Diomedea dabbenena, -1, 0.34, Limosa limosa, -1, 0.01, Neotis denhami, -1, 0.05, Phalacrocorax neglectus, -1, 0.50, Phoebetria fusca, -1, 0.19, Spheniscus demersus, -1, 0.47, Spizocorys fringillaris, -1, 1.00, Thalassarche melanophrys, -2, 0.07, Torgos tracheliotos, -1, 0.05.
- **South Korea** (-0.01): Larus relictus, -1, 0.02, Platalea minor, 1, 0.03.
- Spain (-0.85): Milvus milvus, -1, 0.18, Neophron percnopterus, -3, 0.01, Oxyura leucocephala, -1, 0.05, Puffinus mauretanicus, -2, 0.13, Sylvia undata, -1, 0.29.
- **Sudan** (-2.12): Acrocephalus griseldis, -2, 0.04, Balearica pavonina, -2, 0.44, Coracias garrulus, -1, 0.08, Falco cherrug, -2, 0.06, Limosa limosa, -1, 0.07, Neophron percnopterus, -3, 0.07, Neotis denhami, -1, 0.17, Rynchops flavirostris, -1, 0.07, Torgos tracheliotos, -1, 0.17, Vanellus gregarius, -1, 0.27.
- **Sweden** (-0.01): Haliaeetus albicilla, 1, 0.02, Numenius arquata, -1, 0.02.
- **Syria** (-0.07): Serinus syriacus, -1, 0.02, Sitta krueperi, -1, 0.01.
- **Tajikistan** (-0.09): Columba eversmanni, -1, 0.03.
- **Tanzania** (-2.45): Acrocephalus griseldis, -2, 0.22, Anthus sokokensis, -1, 0.53, Ardeola idae, -1, 0.24, Coracias garrulus, -1, 0.03, Limosa limosa, -1, 0.04, Neotis denhami, -1, 0.03, Rynchops flavirostris, -1, 0.09, Torgos tracheliotos, -1, 0.03, Xenoperdix udzungwensis, -1, 1.00.
- **Thailand** (-2.58): Aceros comatus, -1, 0.08, Actenoides concretus, -1, 0.03, Aegithina viridissima, -1, 0.02, Alcedo euryzona, -1, 0.04, Anthreptes rhodolaemus, -1, 0.07, Argusianus argus, -1, 0.04, Aythya baeri, -1, 0.02, Buceros rhinoceros, -1, 0.02, Caloperdix oculeus, -1, 0.16, Calyptomena viridis, -1, 0.06, Chloropsis cyanopogon, -1, 0.05, Cuculus vagans, -1, 0.07, Dinopium rafflesii, -1, 0.03, Emberiza aureola, -1, 0.02, Enicurus ruficapillus, -1, 0.04, Eupetes macrocerus, -1, 0.05, Eurylaimus ochromalus, -1, 0.08, Ficedula dumetoria, -1, 0.03, Gyps bengalensis, -3, 0.10, Harpactes diardii, -1, 0.06, Harpactes duvaucelii, -1, 0.06, Harpactes kasumba, -1, 0.02, Harpactes orrhophaeus, -1, 0.02, Iole olivacea, -1, 0.03, Ixos malaccensis, -1, 0.03, Kenopia striata, -1, 0.05, Macronous ptilosus, -1, 0.02, Malacocincla malaccensis, -1, 0.02, Malacopteron affine, -1, 0.03, Malacopteron magnum, -1, 0.02, Megalaima henricii, -1, 0.02, Megalaima mystacophanos, -1, 0.07, Meiglyptes tukki, -1, 0.03, Oriolus xanthonotus, -1, 0.02, Pericrocotus igneus, -1, 0.06, Phaenicophaeus diardi, -1, 0.06, Phaenicophaeus sumatranus,

-1, 0.06, Philentoma velata, -1, 0.03, Platylophus galericulatus, -1, 0.04, Pycnonotus cyaniventris, -1, 0.03, Pycnonotus eutilotus, -1, 0.04, Pycnonotus melanoleucos, -1, 0.02, Pycnonotus squamatus, -1, 0.02, Rhizothera longirostris, -1, 0.08, Rollulus rouloul, -1, 0.05, Stachyris leucotis, -1, 0.09, Stachyris maculata, -1, 0.02, Stachyris nigricollis, -1, 0.15, Treron capellei, -1, 0.03.

- **The Bahamas** (0.47): Dendroica kirtlandii, 1, 0.47.
- **Tonga** (0.98): Megapodius pritchardii, 1, 1.00, Pterodroma alba, -1, 0.05, Pterodroma cookii, 1, 0.03.
- **Tunisia** (-0.23): Oxyura leucocephala, -1, 0.05, Puffinus mauretanicus, -2, 0.02, Sylvia undata, -1, 0.09.
- **Turkey** (-1.21): Coracias garrulus, -1, 0.03, Falco cherrug, -2, 0.01, Neophron percnopterus, -3, 0.04, Numenius arquata, -1, 0.01, Oxyura leucocephala, -1, 0.06, Sitta krueperi, -1, 0.85, Tetrao mlokosiewiczi, -1, 0.11.
- **Turkmenistan** (-0.26): Chlamydotis undulata, -2, 0.03, Columba eversmanni, -1, 0.07, Falco cherrug, -2, 0.01, Haliaeetus albicilla, 1, 0.01, Neophron percnopterus, -3, 0.03, Oxyura leucocephala, -1, 0.04.
- **Tuvalu** (-0.03): Pterodroma alba, -1, 0.03.
- **Uganda** (-0.28): Acrocephalus griseldis, -2, 0.02, Ardeola idae, -1, 0.04, Bradypterus graueri, -1, 0.11, Neotis denhami, -1, 0.01, Rynchops flavirostris, -1, 0.02, Torgos tracheliotos, -1, 0.02.
- **Ukraine** (-0.16): Coracias garrulus, -1, 0.02, Falco cherrug, -2, 0.03, Haliaeetus albicilla, 1, 0.11, Limosa limosa, -1, 0.02, Milvus milvus, -1, 0.13, Numenius arquata, -1, 0.03.
- **United Kingdom** (-3.99): Charadrius sanctaehelenae, -1, 1.00, Cichlherminia lherminieri, -1, 0.05, Dendroica kirtlandii, 1, 0.02, Diomedea dabbenena, -1, 0.26, Icterus oberi, -2, 1.00, Numenius arquata, -1, 0.01, Phoebetria fusca, -1, 0.13, Pterodroma alba, -1, 0.03, Pterodroma cookii, 1, 0.02, Puffinus mauretanicus, -2, 0.15, Thalassarche melanophrys, -2, 0.13.
- **United States** (-30.92): Acrocephalus luscinius, -1, 1.00, Agelaius tricolor, -3, 0.99, Brachyramphus brevirostris, -4, 0.76, Chaetura pelagica, -1, 0.43, Cleptornis marchei, -2, 1.00, Corvus hawaiiensis, -1, 1.00, Corvus kubaryi, -1, 1.00, Dendroica kirtlandii, 1, 0.51, Gymnogyps californianus, 1, 0.99, Hemignathus lucidus, -1, 1.00, Loxioides bailleui, -1, 1.00, Loxops caeruleirostris, -1, 1.00, Melamprosops phaeosoma, -1, 1.00, Myadestes lanaiensis, -1, 1.00, Myadestes myadestinus, -1, 1.00, Palmeria dolei, -2, 1.00, Paroreomyza maculata, -1, 1.00, Paroreomyza montana, -1, 1.00, Patagioenas inornata, 1, 0.19, Phoebastria nigripes, -3, 0.55, Polysticta stelleri, -1, 0.24, Pseudonestor xanthophrys, -2, 1.00, Psittirostra psittacea, -1, 1.00, Pterodroma alba, -1, 0.11, Pterodroma cookii, 1, 0.22, Ptilinopus roseicapilla, -2, 1.00, Puffinus opisthomelas, 1, 0.32, Tympanuchus cupido, -1, 0.83, Vermivora bachmanii, -1, 0.88, Zosterops conspicillatus, -3, 1.00.
- **Uruguay** (-0.08): Diomedea dabbenena, -1, 0.05, Phoebetria fusca, -1, 0.02.
- **Uzbekistan** (-0.35): Chlamydotis undulata, -2, 0.03, Columba eversmanni, -1, 0.08, Neophron percnopterus, -3, 0.02, Oxyura leucocephala, -1, 0.10.
- **Venezuela** (-1.84): Chaetura pelagica, -1, 0.03, Metallura iracunda, -1, 0.49, Pauxi pauxi, -1, 0.88, Schizoeaca perijana, -1, 0.44.
- **Viet Nam** (-0.44): Emberiza aureola, -1, 0.02, Eurynorhynchus pygmeus, -2, 0.03, Gyps bengalensis, -3, 0.03, Houbaropsis bengalensis, -1, 0.05, Platalea minor, 1, 0.09, Pseudibis davisoni, -1, 0.13, Sarcogyps calvus, -3, 0.06.
- **Yemen** (-0.10): Chlamydotis undulata, -2, 0.03.
- **Zambia** (-0.41): Ardeola idae, -1, 0.12, Coracias garrulus, -1, 0.03, Limosa limosa, -1, 0.03, Neotis denhami, -1, 0.10, Rynchops flavirostris, -1, 0.08, Torgos tracheliotos, -1, 0.06.

- **Zimbabwe** (-0.14): Ardeola idae, -1, 0.02, Coracias garrulus, -1, 0.02, Limosa limosa, -1, 0.02, Neotis denhami, -1, 0.01, Rynchops flavirostris, -1, 0.02, Torgos tracheliotos, -1, 0.04.

Mammals (1996 to 2008)

- **Afghanistan** (-0.16): Capra aegagrus, -1, 0.03, Gazella subgutturosa, -1, 0.02, Otocolobus manul, -1, 0.06, Panthera pardus, -1, 0.02, Vormela peregusna, -1, 0.03.
- **Algeria** (-0.48): Felis margarita, -1, 0.23, Gazella dorcas, -1, 0.21, Myotis emarginatus, 1, 0.03, Nanger dama, -1, 0.07.
- **Andorra** (-0.01): Lynx pardinus, -1, 0.01.
- **Angola** (-0.15): Caracal aurata, -1, 0.03, Eidolon helvum, -1, 0.07, Panthera pardus, -1, 0.06.
- **Argentina** (-0.56): Abrothrix sanborni, -1, 0.03, Lontra provocax, -1, 0.75, Tapirus terrestris, -1, 0.02, Tayassu pecari, -1, 0.02, Vicugna vicugna, 1, 0.24.
- **Australia** (-11.03): Balaenoptera musculus, 1, 0.07, Bettongia gaimardi, -1, 1.00, Bettongia lesueur, 2, 1.00, Bettongia penicillata, -3, 1.00, Burramys parvus, -1, 1.00, Dasyurus geoffroii, 1, 1.00, Dasyurus hallucatus, -2, 1.00, Dasyurus viverrinus, -1, 1.00, Dendrolagus lumholtzi, 1, 1.00, Falsistrellus mackenziei, -1, 1.00, Leporillus conditor, 1, 1.00, Macropus irma, 1, 1.00, Megaptera novaeangliae, 2, 0.07, Myrmecobius fasciatus, -1, 1.00, Neophoca cinerea, -1, 1.00, Phocarctos hookeri, -1, 0.21, Pipistrellus murrayi, -1, 1.00, Pseudantechinus bilarni, -1, 1.00, Pseudochirulus cinereus, 1, 1.00, Pseudochirulus herbertensis, 1, 1.00, Pseudomys fieldi, 2, 1.00, Pseudomys fumeus, -1, 1.00, Pseudomys novaehollandiae, -1, 1.00, Pteropus melanotus, -1, 0.02, Sarcophilus harrisii, -3, 1.00, Sminthopsis aitkeni, -1, 1.00, Zyzomys maini, -1, 1.00, Zyzomys pedunculatus, -1, 1.00.
- **Austria** (0.02): Myotis emarginatus, 1, 0.02.
- **Azerbaijan** (0.00): Myotis emarginatus, 1, 0.01.
- **Bangladesh** (-0.12): Aonyx cinerea, -1, 0.01, Lutrogale perspicillata, -1, 0.03, Manis crassicaudata, -1, 0.03, Nycticebus bengalensis, -1, 0.03, Viverra zibetha, -1, 0.01.
- **Belarus** (0.42): Bison bonasus, 1, 0.41, Spermophilus suslicus, 1, 0.02.
- **Belize** (-0.04): Balantiopteryx io, -1, 0.04.
- **Bhutan** (-0.03): Arctictis binturong, -1, 0.01, Ratufa bicolor, -1, 0.01, Rhinoceros unicornis, 1, 0.01.
- **Bolivia** (-0.11): Anoura cultrata, -1, 0.08, Dinomys branickii, -1, 0.05, Tapirus terrestris, -1, 0.07, Tayassu pecari, -1, 0.06, Vicugna vicugna, 1, 0.16.
- **Bosnia and Herzegovina** (0.01): Myotis emarginatus, 1, 0.01.
- **Botswana** (-0.03): Panthera pardus, -1, 0.03.
- **Brazil** (-1.63): Balaenoptera musculus, 1, 0.03, Dinomys branickii, -1, 0.39, Leontopithecus rosalia, 1, 1.00, Lonchorhina orinocensis, -1, 0.03, Megaptera novaeangliae, 2, 0.03, Saimiri ustus, -1, 0.99, Tapirus terrestris, -1, 0.65, Tayassu pecari, -1, 0.65.
- **Brunei Darussalam** (-0.12): Hylobates muelleri, -1, 0.01, Nasalis larvatus, -1, 0.01, Prionailurus planiceps, -1, 0.02.
- **Bulgaria** (0.01): Myotis emarginatus, 1, 0.02, Vormela peregusna, -1, 0.01.
- **Burkina Faso** (-0.03): Eidolon helvum, -1, 0.02.
- **Cambodia** (-1.28): Aonyx cinerea, -1, 0.04, Arctictis binturong, -1, 0.04, Arctonyx collaris, -1, 0.03, Lutra sumatrana, -1, 0.05, Lutrogale perspicillata, -1, 0.04, Manis javanica, -1, 0.08, Nomascus gabriellae, -1, 0.25, Nycticebus bengalensis, -1, 0.06, Prionailurus viverrinus, -1, 0.12, Pteropus lylei, -1, 0.34, Pteropus vampyrus, -1, 0.02, Ratufa bicolor, -1, 0.07, Viverra megaspila, -1, 0.12, Viverra zibetha, -1, 0.04.

- **Cameroon** (-0.35): Caracal aurata, -1, 0.07, Eidolon helvum, -1, 0.04, Gorilla gorilla, -1, 0.23.
- **Canada** (-1.67): Balaenoptera musculus, 1, 0.02, Enhydra lutris, -2, 0.01, Marmota vancouverensis, -1, 1.00, Megaptera novaeangliae, 2, 0.02, Ursus maritimus, -2, 0.35.
- **Central African Republic** (-0.09): Caracal aurata, -1, 0.01, Eidolon helvum, -1, 0.02, Gorilla gorilla, -1, 0.03, Panthera pardus, -1, 0.03.
- **Chad** (-0.47): Addax nasomaculatus, -1, 0.09, Gazella dorcas, -1, 0.08, Nanger dama, -1, 0.29.
- **Chile** (-2.19): Abrothrix sanborni, -1, 0.97, Amorphochilus schnablii, -1, 0.18, Balaenoptera musculus, 1, 0.03, Chinchilla lanigera, -1, 1.00, Lontra provocax, -1, 0.25, Megaptera novaeangliae, 2, 0.03, Vicugna vicugna, 1, 0.12.
- **China** (-10.94): Aonyx cinerea, -1, 0.18, Arctictis binturong, -1, 0.03, Arctonyx collaris, -1, 0.66, Camelus ferus, -1, 0.75, Equus hemionus, -2, 0.23, Gazella subgutturosa, -1, 0.25, Hydropotes inermis, -1, 0.80, Hylobates lar, -1, 0.01, Lipotes vexillifer, -1, 1.00, Lutrogale perspicillata, -1, 0.03, Manis pentadactyla, -1, 0.68, Mustela altaica, -1, 0.52, Nomascus concolor, -1, 0.37, Nomascus leucogenys, -1, 0.47, Nycticebus bengalensis, -1, 0.09, Ochotona iliensis, -1, 1.00, Otocolobus manul, -1, 0.53, Panthera pardus, -1, 0.12, Pantholops hodgsonii, -1, 1.00, Procapra picticaudata, -1, 0.99, Pteropus lylei, -1, 0.03, Ratufa bicolor, -1, 0.07, Viverra megaspila, -1, 0.15, Viverra zibetha, -1, 0.51, Vormela peregusna, -1, 0.24.
- **Colombia** (-3.14): Anoura cultrata, -1, 0.40, Dinomys branickii, -1, 0.16, Lonchorhina orinocensis, -1, 0.55, Platyrrhinus chocoensis, -1, 0.70, Rhogeessa minutilla, -1, 0.24, Saguinus leucopus, -1, 1.00, Tapirus terrestris, -1, 0.05, Tayassu pecari, -1, 0.05.
- **Cook Islands** (0.04): Balaenoptera musculus, 1, 0.01, Megaptera novaeangliae, 2, 0.01.
- **Costa Rica** (-0.01): Anoura cultrata, -1, 0.02.
- **Croatia** (0.01): Myotis emarginatus, 1, 0.01.
- **Cuba** (-0.99): Mysateles meridionalis, -1, 1.00.
- **Czech Republic** (0.01): Myotis emarginatus, 1, 0.01.
- **Democratic Republic of the Congo** (-2.74): Caracal aurata, -1, 0.43, Dendromus kahuziensis, -2, 1.00, Eidolon helvum, -1, 0.20, Panthera pardus, -1, 0.11.
- **Denmark** (-0.27): Balaenoptera musculus, 1, 0.01, Megaptera novaeangliae, 2, 0.01, Ursus maritimus, -2, 0.16.
- **Ecuador** (-3.46): Amorphochilus schnablii, -1, 0.15, Anoura cultrata, -1, 0.08, Arctocephalus galapagoensis, -1, 1.00, Caenolestes caniventer, -1, 0.92, Dinomys branickii, -1, 0.02, Platyrrhinus chocoensis, -1, 0.30, Zalophus wollebaeki, -1, 1.00.
- **Egypt** (-0.12): Felis margarita, -1, 0.03, Gazella dorcas, -1, 0.09.
- **Equatorial Guinea** (-0.04): Gorilla gorilla, -1, 0.04.
- **Eritrea** (-0.02): Gazella dorcas, -1, 0.01.
- **Ethiopia** (0.85): Capra walie, 1, 1.00, Eidolon helvum, -1, 0.02, Kobus megaceros, -1, 0.06, Panthera pardus, -1, 0.05.
- **Fiji** (0.88): Pteropus samoensis, 1, 0.85.
- **France** (-0.24): Arvicola sapidus, -1, 0.52, Balaenoptera musculus, 1, 0.08, Lynx pardinus, -1, 0.05, Megaptera novaeangliae, 2, 0.08, Myotis emarginatus, 1, 0.12.
- **Gabon** (-0.46): Caracal aurata, -1, 0.06, Eidolon helvum, -1, 0.02, Gorilla gorilla, -1, 0.36, Panthera pardus, -1, 0.01.
- **Georgia** (-0.42): Capra caucasica, -1, 0.43, Myotis emarginatus, 1, 0.02.
- **Germany** (0.02): Myotis emarginatus, 1, 0.02.
- **Ghana** (-0.07): Caracal aurata, -1, 0.04, Eidolon helvum, -1, 0.02, Panthera pardus, -1, 0.01.

- **Greece** (0.02): Myotis emarginatus, 1, 0.03.
- **Guatemala** (-0.36): Balantiopteryx io, -1, 0.36.
- **Guinea** (-0.06): Caracal aurata, -1, 0.03, Eidolon helvum, -1, 0.02, Panthera pardus, -1, 0.01.
- **Guyana** (-0.03): Tapirus terrestris, -1, 0.02, Tayassu pecari, -1, 0.02.
- **Hungary** (0.02): Myotis emarginatus, 1, 0.02.
- **India** (-4.00): Aonyx cinerea, -1, 0.15, Arctictis binturong, -1, 0.07, Arctonyx collaris, -1, 0.02, Balaenoptera musculus, 1, 0.02, Crocidura nicobarica, -1, 1.00, Equus hemionus, -2, 0.09, Lutrogale perspicillata, -1, 0.46, Manis crassicaudata, -1, 0.90, Manis pentadactyla, -1, 0.05, Megaptera novaeangliae, 2, 0.02, Nycticebus bengalensis, -1, 0.10, Panthera pardus, -1, 0.03, Prionailurus viverrinus, -1, 0.04, Pteropus melanotus, -1, 0.37, Ratufa bicolor, -1, 0.06, Rhinoceros unicornis, 1, 0.49, Semnopithecus hypoleucos, -1, 1.00, Viverra megaspila, -1, 0.05, Viverra zibetha, -1, 0.05.
- **Indonesia** (-40.63): Aonyx cinerea, -1, 0.21, Arctictis binturong, -1, 0.33, Arctonyx collaris, -1, 0.03, Axis kuhlii, -1, 1.00, Balaenoptera musculus, 1, 0.05, Coelops robinsoni, -1, 0.85, Dendrolagus inustus, -1, 0.87, Dendrolagus stellarum, -1, 0.83, Dorcopsis luctuosa, -1, 0.27, Harpyionycteris celebensis, -1, 1.00, Hemigalus derbyanus, -1, 0.71, Hipposideros doriae, -1, 0.77, Hylobates agilis, -1, 0.93, Hylobates albibarbis, -1, 1.00, Hylobates klossii, -1, 1.00, Hylobates lar, -1, 0.11, Hylobates muelleri, -1, 0.62, Kerivoula pellucida, -1, 0.72, Lutra sumatrana, -1, 0.32, Lutrogale perspicillata, -1, 0.10, Macaca hecki, -1, 1.00, Macaca nigra, -1, 1.00, Macaca ochreata, -1, 1.00, Macaca pagensis, -1, 1.00, Macaca tonkeana, -1, 1.00, Manis javanica, -1, 0.48, Megaptera novaeangliae, 2, 0.04, Nasalis larvatus, -1, 0.85, Nycteris tragata, -1, 0.69, Nycticebus coucang, -1, 0.69, Nycticebus javanicus, -2, 1.00, Nycticebus menagensis, -1, 0.73, Pardofelis badia, -1, 0.70, Phoniscus atrox, -1, 0.06, Presbytis melalophos, -1, 1.00, Presbytis potenziani, -1, 1.00, Presbytis siamensis, -1, 0.35, Presbytis thomasi, -1, 1.00, Prionailurus planiceps, -1, 0.65, Prionailurus viverrinus, -1, 0.01, Pseudochirops coronatus, -1, 1.00, Pteropus melanopogon, -1, 1.00, Pteropus melanotus, -1, 0.61, Pteropus vampyrus, -1, 0.55, Ratufa bicolor, -1, 0.19, Rhinolophus sedulus, -1, 0.66, Rousettus bidens, -1, 1.00, Rusa timorensis, -1, 1.00, Simias concolor, -1, 1.00, Spilocuscus rufoniger, -1, 0.42, Spilocuscus wilsoni, -1, 1.00, Sus barbatus, -1, 0.77, Sus celebensis, -1, 1.00, Symphalangus syndactylus, -1, 0.87, Tadarida mops, -1, 0.72, Tapirus indicus, -1, 0.53, Tarsius bancanus, -1, 0.76, Trachypithecus cristatus, -1, 0.81
- **Iran** (-0.91): Capra aegagrus, -1, 0.69, Felis margarita, -1, 0.04, Gazella subgutturosa, -1, 0.16, Myotis emarginatus, 1, 0.12, Otocolobus manul, -1, 0.01, Panthera pardus, -1, 0.04, Vormela peregusna, -1, 0.07.
- **Iraq** (-0.06): Gazella subgutturosa, -1, 0.04, Vormela peregusna, -1, 0.01.
- **Italy** (0.03): Myotis emarginatus, 1, 0.03.
- **Ivory Coast** (-0.09): Caracal aurata, -1, 0.04, Eidolon helvum, -1, 0.03, Panthera pardus, -1, 0.01.
- **Japan** (-0.91): Balaenoptera musculus, 1, 0.03, Megaptera novaeangliae, 2, 0.03, Tokudaia muenninki, -1, 1.00.
- **Jordan** (-0.02): Felis margarita, -1, 0.01.
- **Kazakhstan** (-2.77): Allactaga vinogradovi, -1, 0.62, Equus hemionus, -2, 0.02, Felis margarita, -1, 0.06, Gazella subgutturosa, -1, 0.12, Saiga tatarica, -2, 0.85, Vormela peregusna, -1, 0.23.
- **Kenya** (-0.49): Caracal aurata, -1, 0.04, Cephalophus adersi, -1, 0.41, Eidolon helvum, -1, 0.01, Panthera pardus, -1, 0.03.
- **Kiribati** (0.08): Balaenoptera musculus, 1, 0.03, Megaptera novaeangliae, 2, 0.03.
- **Kyrgyzstan** (-0.20): Allactaga vinogradovi, -1, 0.17, Mustela altaica, -1, 0.02.

- **Laos** (-2.42): Aonyx cinerea, -1, 0.05, Arctictis binturong, -1, 0.07, Arctonyx collaris, -1, 0.03, Hylobates lar, -1, 0.03, Lutrogale perspicillata, -1, 0.05, Manis javanica, -1, 0.06, Manis pentadactyla, -1, 0.05, Nomascus concolor, -1, 0.34, Nomascus gabriellae, -1, 0.33, Nomascus leucogenys, -1, 0.47, Nycticebus bengalensis, -1, 0.11, Prionailurus viverrinus, -1, 0.09, Pseudoryx nghetinhensis, -1, 0.51, Ratufa bicolor, -1, 0.09, Viverra megaspila, -1, 0.09, Viverra zibetha, -1, 0.05.
- **Liberia** (-0.03): Caracal aurata, -1, 0.02.
- **Libya** (-0.16): Gazella dorcas, -1, 0.16.
- **Malaysia** (-12.39): Aonyx cinerea, -1, 0.07, Arctictis binturong, -1, 0.10, Coelops robinsoni, -1, 0.15, Hemigalus derbyanus, -1, 0.24, Hesperoptenus tomesi, -2, 1.00, Hipposideros doriae, -1, 0.23, Hylobates agilis, -1, 0.04, Hylobates lar, -1, 0.18, Hylobates muelleri, -1, 0.37, Kerivoula pellucida, -1, 0.26, Lutra sumatrana, -1, 0.54, Lutrogale perspicillata, -1, 0.03, Manis javanica, -1, 0.14, Murina aenea, -1, 0.90, Murina rozendaali, -1, 1.00, Nasalis larvatus, -1, 0.14, Nycteris tragata, -1, 0.23, Nycticebus coucang, -1, 0.21, Nycticebus menagensis, -1, 0.26, Pardofelis badia, -1, 0.30, Phoniscus atrox, -1, 0.71, Presbytis siamensis, -1, 0.64, Prionailurus planiceps, -1, 0.33, Prionailurus viverrinus, -1, 0.02, Pteropus vampyrus, -1, 0.17, Ratufa bicolor, -1, 0.05, Rhinolophus sedulus, -1, 0.33, Sus barbatus, -1, 0.22, Symphalangus syndactylus, -1, 0.12, Tadarida johorensis, -1, 1.00, Tadarida mops, -1, 0.27, Tapirus indicus, -1, 0.09, Tarsius bancanus, -1, 0.23, Trachypithecus cristatus, -1, 0.19, Trachypithecus obscurus, -1, 0.51, Viverra megaspila, -1, 0.09, Viverra zibetha, -1, 0.03.
- **Mali** (-0.30): Eidolon helvum, -1, 0.01, Gazella dorcas, -1, 0.09, Nanger dama, -1, 0.20.
- **Marshall Islands** (0.04): Balaenoptera musculus, 1, 0.02, Megaptera novaeangliae, 2, 0.01.
- **Mauritania** (-0.29): Addax nasomaculatus, -1, 0.21, Gazella dorcas, -1, 0.08.
- **Mexico** (-5.52): Balaenoptera musculus, 1, 0.03, Balantiopteryx io, -1, 0.59, Megaptera novaeangliae, 2, 0.02, Neotoma palatina, -1, 1.00, Pappogeomys alcorni, -1, 1.00, Procyon pygmaeus, -1, 1.00, Reithrodontomys spectabilis, -1, 1.00, Spilogale pygmaea, -1, 1.00.
- **Micronesia** (0.07): Balaenoptera musculus, 1, 0.02, Megaptera novaeangliae, 2, 0.02.
- **Moldova** (0.02): Spermophilus suslicus, 1, 0.01.
- **Mongolia** (-1.13): Camelus ferus, -1, 0.25, Equus ferus, 1, 1.00, Equus hemionus, -2, 0.60, Gazella subgutturosa, -1, 0.06, Mustela altaica, -1, 0.17, Otocolobus manul, -1, 0.29, Saiga tatarica, -2, 0.03, Vormela peregusna, -1, 0.08.
- **Morocco** (-1.10): Addax nasomaculatus, -1, 0.02, Felis margarita, -1, 0.06, Gazella dorcas, -1, 0.06, Gerbillus hesperinus, -1, 1.00, Myotis emarginatus, 1, 0.04, Nanger dama, -1, 0.01.
- **Mozambique** (-0.06): Eidolon helvum, -1, 0.04, Panthera pardus, -1, 0.03.
- **Myanmar** (-2.41): Aonyx cinerea, -1, 0.09, Arctictis binturong, -1, 0.11, Arctonyx collaris, -1, 0.11, Hylobates lar, -1, 0.14, Lutra sumatrana, -1, 0.05, Lutrogale perspicillata, -1, 0.11, Manis javanica, -1, 0.08, Manis pentadactyla, -1, 0.15, Nycteris tragata, -1, 0.02, Nycticebus bengalensis, -1, 0.27, Panthera pardus, -1, 0.03, Prionailurus viverrinus, -1, 0.39, Pteropus vampyrus, -1, 0.02, Ratufa bicolor, -1, 0.19, Tapirus indicus, -1, 0.19, Trachypithecus obscurus, -1, 0.15, Viverra megaspila, -1, 0.19, Viverra zibetha, -1, 0.14.
- **Namibia** (-0.02): Panthera pardus, -1, 0.03.
- **Nepal** (0.41): Aonyx cinerea, -1, 0.02, Manis pentadactyla, -1, 0.02, Mustela altaica, -1, 0.01, Prionailurus viverrinus, -1, 0.02, Rhinoceros unicornis, 1, 0.50.
- **New Zealand** (-1.69): Balaenoptera musculus, 1, 0.03, Chalinolobus tuberculatus, -1, 1.00, Megaptera novaeangliae, 2, 0.03, Phocarctos hookeri, -1, 0.79.
- **Niger** (-1.33): Addax nasomaculatus, -1, 0.66, Felis margarita, -1, 0.14, Gazella dorcas, -1, 0.10, Nanger dama, -1, 0.43.
- **Nigeria** (-1.04): Crocidura buettikoferi, -1, 0.97, Eidolon helvum, -1, 0.07.

- **North Korea** (-0.11): Hydropotes inermis, -1, 0.09, Mustela altaica, -1, 0.02.
- **Norway** (-0.18): Balaenoptera musculus, 1, 0.02, Megaptera novaeangliae, 2, 0.02, Ursus maritimus, -2, 0.11.
- **Oman** (-0.05): Felis margarita, -1, 0.04, Gazella subgutturosa, -1, 0.03.
- **Pakistan** (-0.14): Capra aegagrus, -1, 0.04, Felis margarita, -1, 0.01, Manis crassicaudata, -1, 0.05, Vormela peregusna, -1, 0.01.
- **Panama** (-0.05): Anoura cultrata, -1, 0.05.
- **Papua New Guinea** (-12.03): Balaenoptera musculus, 1, 0.02, Dendrolagus inustus, -1, 0.13, Dendrolagus scottae, -1, 1.00, Dendrolagus stellarum, -1, 0.17, Dorcopsis luctuosa, -1, 0.73, Megaptera novaeangliae, 2, 0.02, Peroryctes broadbenti, -1, 1.00, Phalanger lullulae, -3, 1.00, Phalanger matanim, -1, 1.00, Pteropus capistratus, -1, 1.00, Solomys ponceleti, -1, 0.74, Solomys salebrosus, -1, 0.74, Spilocuscus kraemeri, -1, 1.00, Spilocuscus rufoniger, -1, 0.58, Uromys neobritannicus, -1, 1.00.
- **Paraguay** (-0.06): Tapirus terrestris, -1, 0.03, Tayassu pecari, -1, 0.03.
- **Peru** (-1.03): Amorphochilus schnablii, -1, 0.67, Anoura cultrata, -1, 0.28, Caenolestes caniventer, -1, 0.08, Dinomys branickii, -1, 0.37, Tapirus terrestris, -1, 0.07, Tayassu pecari, -1, 0.07, Vicugna vicugna, 1, 0.48.
- **Philippines** (-4.13): Acerodon leucotis, -1, 1.00, Balaenoptera musculus, 1, 0.01, Bubalus mindorensis, -1, 1.00, Kerivoula pellucida, -1, 0.01, Manis culionensis, -1, 1.00, Megaptera novaeangliae, 2, 0.01, Pteropus vampyrus, -1, 0.15, Tragulus nigricans, -1, 1.00.
- **Poland** (0.59): Bison bonasus, 1, 0.59.
- **Portugal** (-0.07): Arvicola sapidus, -1, 0.09, Balaenoptera musculus, 1, 0.01, Lynx pardinus, -1, 0.05, Megaptera novaeangliae, 2, 0.01, Myotis emarginatus, 1, 0.02.
- **Republique du Congo** (-0.45): Caracal aurata, -1, 0.08, Eidolon helvum, -1, 0.03, Gorilla gorilla, -1, 0.33, Panthera pardus, -1, 0.02.
- **Romania** (0.05): Myotis emarginatus, 1, 0.05.
- **Russia** (-2.00): Balaenoptera musculus, 1, 0.01, Capra caucasica, -1, 0.57, Enhydra lutris, -2, 0.46, Megaptera novaeangliae, 2, 0.03, Mustela altaica, -1, 0.21, Otocolobus manul, -1, 0.10, Saiga tatarica, -2, 0.06, Spermophilus suslicus, 1, 0.61, Ursus maritimus, -2, 0.34, Vormela peregusna, -1, 0.07.
- **Samoa** (0.15): Pteropus samoensis, 1, 0.14.
- **Saudi Arabia** (-0.31): Equus hemionus, -2, 0.01, Felis margarita, -1, 0.12, Gazella subgutturosa, -1, 0.16.
- **Senegal** (-0.95): Oryx dammah, -1, 0.95.
- **Serbia** (0.01): Myotis emarginatus, 1, 0.02.
- **Seychelles** (0.03): Balaenoptera musculus, 1, 0.01.
- **Sierra Leone** (-0.02): Caracal aurata, -1, 0.02.
- **Slovakia** (0.01): Myotis emarginatus, 1, 0.01.
- **Solomon Islands** (-3.49): Balaenoptera musculus, 1, 0.01, Megaptera novaeangliae, 2, 0.01, Pteropus woodfordi, -1, 1.00, Solomys ponceleti, -1, 0.26, Solomys salebrosus, -1, 0.26, Solomys sapientis, -1, 1.00, Uromys rex, -1, 1.00.
- **Somalia** (-1.02): Gazella spekei, -1, 1.00, Panthera pardus, -1, 0.03.
- **South Africa** (-0.05): Balaenoptera musculus, 1, 0.01, Eidolon helvum, -1, 0.06, Megaptera novaeangliae, 2, 0.01, Panthera pardus, -1, 0.02.
- **South Korea** (-0.12): Hydropotes inermis, -1, 0.11, Mustela altaica, -1, 0.02.
- **Spain** (-0.15): Arvicola sapidus, -1, 0.39, Capra pyrenaica, 1, 0.99, Lynx pardinus, -1, 0.89, Myotis emarginatus, 1, 0.11.
- **Sri Lanka** (-0.06): Manis crassicaudata, -1, 0.02, Prionailurus viverrinus, -1, 0.05.

- **Sudan** (-1.22): Caracal aurata, -1, 0.03, Eidolon helvum, -1, 0.08, Gazella dorcas, -1, 0.11, Kobus megaceros, -1, 0.94, Panthera pardus, -1, 0.06.
- **Suriname** (-0.02): Tapirus terrestris, -1, 0.01, Tayassu pecari, -1, 0.01.
- **Syria** (-0.02): Gazella subgutturosa, -1, 0.02, Vormela peregusna, -1, 0.01.
- **Tajikistan** (0.00): Mustela altaica, -1, 0.01, Myotis emarginatus, 1, 0.02.
- **Tanzania** (0.29): Cephalophus adersi, -1, 0.59, Cephalophus spadix, -1, 1.00, Eidolon helvum, -1, 0.08, Panthera pardus, -1, 0.04, Pteropus voeltzkowi, 2, 1.00.
- **Thailand** (-3.50): Aonyx cinerea, -1, 0.11, Arctictis binturong, -1, 0.13, Arctonyx collaris, -1, 0.06, Hemigalus derbyanus, -1, 0.04, Hylobates agilis, -1, 0.03, Hylobates lar, -1, 0.52, Lutra sumatrana, -1, 0.01, Lutrogale perspicillata, -1, 0.10, Manis javanica, -1, 0.11, Murina aenea, -1, 0.10, Nycteris tragata, -1, 0.06, Nycticebus bengalensis, -1, 0.24, Nycticebus coucang, -1, 0.10, Phoniscus atrox, -1, 0.23, Prionailurus viverrinus, -1, 0.14, Pteropus lylei, -1, 0.45, Pteropus vampyrus, -1, 0.06, Ratufa bicolor, -1, 0.16, Tapirus indicus, -1, 0.19, Trachypithecus obscurus, -1, 0.34, Viverra megaspila, -1, 0.17, Viverra zibetha, -1, 0.11.
- **Tunisia** (-0.08): Addax nasomaculatus, -1, 0.03, Oryx dammah, -1, 0.05.
- **Turkey** (-0.23): Capra aegagrus, -1, 0.21, Myotis emarginatus, 1, 0.04, Vormela peregusna, -1, 0.06.
- **Turkmenistan** (-0.23): Capra aegagrus, -1, 0.01, Equus hemionus, -2, 0.03, Felis margarita, -1, 0.11, Gazella subgutturosa, -1, 0.05, Myotis emarginatus, 1, 0.07, Vormela peregusna, -1, 0.06.
- **Uganda** (-0.08): Caracal aurata, -1, 0.05, Eidolon helvum, -1, 0.02, Panthera pardus, -1, 0.01.
- **Ukraine** (0.37): Myotis emarginatus, 1, 0.01, Spermophilus suslicus, 1, 0.36.
- **United Arab Emirates** (-0.02): Felis margarita, -1, 0.01.
- **United Kingdom** (0.15): Balaenoptera musculus, 1, 0.05, Megaptera novaeangliae, 2, 0.05.
- **United States** (0.10): Arborimus longicaudus, -1, 1.00, Balaenoptera musculus, 1, 0.07, Dipodomys nitratoides, -1, 1.00, Enhydra lutris, -2, 0.52, Megaptera novaeangliae, 2, 0.07, Monachus schauinslandi, -1, 1.00, Mustela nigripes, 2, 1.00, Myotis grisescens, 1, 1.00, Spermophilus washingtoni, 1, 1.00, Ursus maritimus, -2, 0.04.
- **Uzbekistan** (-0.46): Allactaga vinogradovi, -1, 0.21, Felis margarita, -1, 0.09, Gazella subgutturosa, -1, 0.04, Myotis emarginatus, 1, 0.04, Saiga tatarica, -2, 0.06, Vormela peregusna, -1, 0.05.
- **Venezuela** (-1.38): Anoura cultrata, -1, 0.09, Lonchorhina orinocensis, -1, 0.42, Rhogeessa minutilla, -1, 0.76, Tapirus terrestris, -1, 0.07, Tayassu pecari, -1, 0.05.
- **Viet Nam** (-3.33): Aonyx cinerea, -1, 0.07, Arctictis binturong, -1, 0.09, Arctonyx collaris, -1, 0.05, Lutra sumatrana, -1, 0.02, Lutrogale perspicillata, -1, 0.04, Manis javanica, -1, 0.05, Manis pentadactyla, -1, 0.04, Nomascus concolor, -1, 0.29, Nomascus gabriellae, -1, 0.43, Nomascus leucogenys, -1, 0.06, Nycticebus bengalensis, -1, 0.09, Prionailurus viverrinus, -1, 0.12, Pseudoryx nghetinhensis, -1, 0.49, Pteropus lylei, -1, 0.18, Pteropus vampyrus, -1, 0.02, Pygathrix cinerea, -1, 1.00, Ratufa bicolor, -1, 0.10, Viverra megaspila, -1, 0.15, Viverra zibetha, -1, 0.06.
- **Yemen** (-0.02): Felis margarita, -1, 0.01, Gazella subgutturosa, -1, 0.02.
- **Zambia** (-0.08): Eidolon helvum, -1, 0.05, Panthera pardus, -1, 0.03.
- **Zimbabwe** (-0.05): Eidolon helvum, -1, 0.03, Panthera pardus, -1, 0.02.
