# Supplementary material for: Spatially Explicit Trends in the Global Conservation Status of Vertebrates
Source: PLoS One. 2014 Nov 26;9(11):e113934. doi: 10.1371/journal.pone.0113934 (PMC4245261; doi:10.1371/journal.pone.0113934)
Supplement: Supporting Information S1 — Supporting Materials and Methods. Figure S1, Variation across hexagons in the weighted change in Red List status per year, for different taxonomic groups. Figure S2, Variation across ecoregions in the weighted change in Red List status per year, for different taxonomic groups. Figure S3, Weighted impact of each threat to the deterioration in global species conservation status, across hexagons. Figure S4, Weighted impact of each threat to the deterioration in global species conservation status, across ecoregions. Figure S5, Relationship between each country's responsibility to conservation and its contribution to changes in the global conservation status of birds, mammals and amphibians. Figure S6, Sensitivity to variation in knowledge of the relationship between each country's responsibility to conservation and its contribution to changes in species global conservation status. Figure S7, Sensitivity of the results to possible spilage across countries. Table S1, Absolute weighted Red List change per country, and list of the species driving those values. Table S2, Main results per country. (ZIP) [file pone.0113934.s001.zip › Rodrigues_etal_Supporting Table S2.pdf]

Spatially explicit trends in the global conservation status of vertebrates

Ana S. L. Rodrigues, Thomas M. Brooks, Stuart H.M. Butchart, Janice Chanson, Neil Cox, Michael Hoffmann, Simon N. Stuart

Suplementary Table S2. Key results per country. Countries include dependent territories. Values are calculated across the marine and land territory of each country combined (as in Figures 4 and S5, but unlike Figures 1-3 and S1-S4). Code: ISO 3166-1 country code. *E:* weighted endemism, a measure of each country's responsibility towards global species conservation (Equation 3; the sum across countries equals the total number of species in the group – amphibians, birds, mammals and all three taxa combined). *T*: weighted threat, a measure of the responsibility of each country towards the conservation of threatened species (Equation 4; the sum across countries equals the total number of threatened amphibians in the group). *W*: weighted Red List change per year, the net contribution of each country to the global change in species conservation status per year (Equation 1l the sum across countries is the net number of step changes per year in the group). GDP per capita: Gross Domestic Product based on purchasing-power-parity per capita, in 2009 (unit: current US dollars). GDP: Gross domestic product in 2009 (unit: current 1,000,000,000 US dollars). GEF5: allocations for biodiversity under the Global Environment Facility fifth replenishment (2010-2014), per country, under the System for Transparent Allocation of Resources (STAR) (unit: 1,000,000 US dollars). *H, I* and *AL*: relative importance of, respectively, hunting and trapping, invasive species, and agriculture and logging, to the deterioration in Red List status per country (see section 2.6). Threat code: code of the dominant threat (or combination of threats) responsible for the deterioration in Red List status in each country (used in Figures 4 and S5).

COUNTRY		Amphibians			Birds			Mammals			Combined			Economic data			Threats			
**Name**	**Code**	***E***	***T***	***W***	***E***	***T***	***W***	***E***	***T***	***W***	***E***	***T***	***W***	**GDP per capita**	**GDP**	**GEF5**	**H**	**I**	**AL**	**Threat code**
**Afghanistan**	**AFG**	1.78	1.00	0.00	14.05	0.15	-0.01	10.48	0.52	-0.01	26.32	1.67	-0.02	935.0	10.6	3.35	66	0	34	H
**Albania**	**ALB**	1.46	0.96	-0.07	0.47	0.02	0.00	1.34	0.18	0.00	3.27	1.16	-0.07	7163.8	12.3	1.5	50	50	0	H-I
**Algeria**	**DZA**	3.59	1.50	0.00	15.74	1.22	-0.03	15.85	2.03	-0.04	35.18	4.75	-0.07	6869.1	166.5	3.87	48	0	52	H-AL
**Andorra**	**AND**	0.02	0.00	0.00	0.01	0.00	0.00	0.06	0.02	0.00	0.08	0.02	0.00	-	3.2	-	4	94	2	I
**Angola**	**AGO**	34.97	0.00	0.00	92.11	6.71	-0.01	41.89	0.51	-0.01	168.97	7.22	-0.02	6116.5	84.9	6.99	70	2	28	H
**Antigua and Barbuda**	**ATG**	0.15	0.00	0.00	1.61	0.03	0.00	0.25	0.00	0.00	2.01	0.03	0.00	17892.9	1.2	1.5	0	50	50	I-AL
**Argentina**	**ARG**	75.42	25.78	-0.23	212.52	13.71	-0.12	148.10	18.44	-0.05	436.04	57.93	-0.39	14560.9	328.5	14.61	23	32	45	I-AL
**Armenia**	**ARM**	0.05	0.00	0.00	0.57	0.01	-0.01	3.98	2.31	0.00	4.60	2.32	-0.01	4966.0	11.9	1.5	45	0	55	H-AL
**Australia**	**AUS**	215.51	47.00	-1.82	480.94	38.83	-0.16	266.57	52.58	-0.92	963.02	138.42	-2.89	38910.8	1015.2	-	0	78	22	I
**Austria**	**AUT**	0.89	0.00	0.00	0.90	0.00	0.00	2.97	1.02	0.00	4.75	1.02	0.00	38838.9	413.5	-	13	40	47	I-AL
**Azerbaijan**	**AZE**	0.29	0.00	0.00	1.58	0.07	0.00	5.62	0.27	0.00	7.50	0.34	-0.01	9563.9	46.1	1.5	48	2	50	H-AL
**Bahrain**	**BHR**	0.00	0.00	0.00	0.02	0.01	0.00	0.01	0.00	0.00	0.03	0.01	0.00	27068.0	21.9	-	0	0	100	AL
**Bangladesh**	**BGD**	2.69	0.00	0.00	8.58	1.09	-0.02	2.69	0.96	-0.01	13.96	2.04	-0.03	1465.0	79.6	1.88	36	0	64	H-AL
**Barbados**	**BRB**	0.08	0.00	0.00	1.44	0.05	0.00	0.56	0.28	0.00	2.08	0.33	0.00	18130.6	3.7	1.5	-	-	-	-
**Belarus**	**BRL**	0.51	0.00	0.00	2.25	0.05	0.00	1.73	0.41	0.04	4.50	0.46	0.03	12737.1	60.3	1.5	48	7	44	H-AL
**Belgium**	**BEL**	0.14	0.00	0.00	0.26	0.00	0.00	0.25	0.00	0.00	0.66	0.00	0.00	35421.6	504.2	-	34	25	41	H-AL
**Belize**	**BLZ**	5.84	2.00	-0.08	7.83	0.23	0.00	2.19	0.29	0.00	15.86	2.52	-0.08	7718.5	1.4	2.44	0	68	32	I
**Benin**	**BEN**	1.01	0.00	0.00	5.30	0.05	0.00	3.89	0.29	0.00	10.20	0.34	0.00	1444.6	6.7	1.5	57	0	43	H-AL
**Bhutan**	**BTN**	1.09	0.08	0.00	12.86	1.05	0.00	4.13	1.87	0.00	18.08	3.00	-0.01	5212.2	1.3	1.96	24	0	76	AL
**Bolivia**	**BOL**	101.37	31.07	-0.48	193.03	13.33	-0.19	88.14	7.33	-0.01	382.53	51.73	-0.68	4455.0	16.7	11.44	45	21	35	H-AL
**Bosnia and Herzegovina**	**BIH**	0.53	0.29	0.00	0.57	0.00	0.00	1.35	0.36	0.00	2.45	0.65	0.00	7361.1	18.5	1.5	11	38	52	I-AL
**Botswana**	**BWA**	2.78	0.00	0.00	29.33	0.52	-0.01	13.04	0.91	0.00	45.15	1.42	-0.01	13992.4	13.4	2.11	69	0	31	H
**Brazil**	**BRA**	677.22	27.36	-0.23	938.85	96.79	-0.28	416.67	66.42	-0.14	2032.75	190.57	-0.64	10513.8	1575.2	68.22	42	13	45	H-AL
**Brunei Darussalam**	**BRN**	0.91	0.09	-0.02	1.30	0.09	-0.02	1.22	0.38	-0.01	3.43	0.56	-0.05	49109.9	11.5	-	12	0	88	AL

**Bulgaria**	**BGR**	0.64	0.00	0.00	1.36	0.03	0.00	3.02	0.57	0.00	5.02	0.60	0.00	11900.3	49.9	-	34	13	53	H-AL
**Burkina Faso**	**BFA**	1.75	0.00	0.00	9.82	0.16	-0.01	5.83	0.11	0.00	17.40	0.27	-0.01	1304.5	7.9	1.5	59	0	41	H-AL
**Burundi**	**BDI**	3.53	0.56	0.00	8.46	0.97	-0.01	3.66	1.01	0.00	15.66	2.54	-0.01	400.2	1.2	1.5	11	2	88	AL
**Cambodia**	**KHM**	12.60	1.02	-0.01	23.26	3.16	-0.06	12.15	4.73	-0.11	48.01	8.91	-0.18	2015.0	10.4	3.85	56	0	44	H-AL
**Cameroon**	**CMR**	101.24	50.44	-0.07	74.51	9.07	-0.05	58.68	25.20	-0.03	234.42	84.71	-0.15	2146.9	23.4	10.31	68	13	20	H
**Canada**	**CAN**	11.21	0.03	-0.03	87.74	3.05	-0.02	54.30	1.72	-0.14	153.25	4.80	-0.18	38025.3	1501.3	-	0	49	51	I-AL
**Cape Verde**	**CPV**	0.00	0.00	0.00	6.19	2.00	0.00	0.38	0.03	0.00	6.57	2.03	0.00	3587.8	1.6	3.52	78	0	22	H
**Central African Republic**	**CAF**	7.75	0.00	0.00	29.29	0.10	-0.01	19.09	0.23	-0.01	56.12	0.33	-0.02	745.0	2.0	1.68	61	12	28	H-AL
**Chad**	**TCD**	4.33	0.00	0.00	22.56	0.58	-0.03	7.85	1.00	-0.04	34.74	1.58	-0.07	1611.7	8.4	1.91	60	0	40	H-AL
**Chile**	**CHL**	50.47	18.96	-0.66	76.30	9.26	-0.07	48.57	9.94	-0.18	175.34	38.15	-0.91	14340.9	169.5	18.09	30	20	50	H-AL
**China**	**CHN**	282.31	95.39	-1.40	402.18	41.15	-0.13	271.39	37.28	-0.91	955.88	173.82	-2.45	6567.0	4542.4	52.67	67	3	30	H
**Colombia**	**COL**	470.13	186.56	-2.69	412.44	62.33	-0.19	122.56	23.61	-0.26	1005.14	272.51	-3.15	8936.4	243.8	37.49	2	60	38	I-AL
**Comoros**	**COM**	0.00	0.00	0.00	15.54	6.07	0.00	4.68	2.01	0.00	20.22	8.08	0.00	1159.6	0.5	2.08	27	16	57	H-AL
**Cook Islands**	**COK**	0.00	0.00	0.00	9.33	6.77	0.05	0.48	0.06	0.00	9.81	6.84	0.05	-		2.14	-	-	-	-
**Costa Rica**	**CRI**	92.69	41.52	-1.73	76.20	9.18	0.00	21.74	1.82	0.00	190.63	52.53	-1.73	10579.3	29.7	11.27	1	83	16	I
**Croatia**	**HRV**	0.92	0.58	0.00	0.82	0.01	0.00	1.08	0.18	0.00	2.82	0.77	0.00	17703.3	69.3	1.5	6	32	62	I-AL
**Cuba**	**CUB**	60.66	48.79	-0.08	39.21	11.88	0.00	23.21	12.12	-0.08	123.08	72.79	-0.16	-		11.52	33	42	25	H-I
**Cyprus**	**CYP**	0.05	0.00	0.00	0.25	0.00	0.00	2.01	0.01	0.00	2.31	0.01	0.00	28544.1	24.9	-	31	0	69	AL
**Czech Republic**	**CZE**	0.45	0.00	0.00	0.71	0.00	0.00	0.81	0.07	0.00	1.96	0.07	0.00	24093.3	215.5	-	36	24	40	H-AL
**Democratic Republic of the Congo**	**COD**	100.97	8.36	0.00	223.88	16.83	-0.06	137.99	13.34	-0.23	462.85	38.53	-0.29	332.0	11.7	13.81	24	1	75	AL
**Denmark**	**DNK**	0.08	0.00	0.00	5.30	0.01	0.00	3.61	0.69	-0.02	8.99	0.71	-0.02	35757.3	341.3	-	64	5	31	H-AL
**Djibouti**	**DJI**	0.10	0.00	0.00	1.92	1.01	-0.05	0.86	0.09	0.00	2.87	1.10	-0.05	2484.0	0.9	1.5	1	0	99	AL
**Dominica**	**DMA**	2.18	1.97	-0.05	4.76	2.34	-0.02	0.87	0.34	0.00	7.81	4.65	-0.06	10176.9	0.4	1.5	19	49	32	I-AL
**Dominican Republic**	**DOM**	24.53	20.50	-0.37	26.12	7.43	-0.02	5.66	2.48	0.00	56.31	30.41	-0.39	8896.2	45.5	5.36	4	43	52	I-AL
**East Timor**	**TMP**	1.00	0.00	0.00	17.75	2.17	-0.05	4.20	0.51	0.00	22.95	2.69	-0.05	2521.7	0.5	1.5	30	0	70	AL

**Ecuador**	**ECU**	255.59	133.81	-2.63	229.75	42.99	-0.20	66.48	19.66	-0.29	551.81	196.47	-3.12	7880.7	54.7	24.37	4	58	38	I-AL
**Egypt**	**EGY**	1.25	0.00	0.00	6.51	0.63	-0.03	12.39	1.33	-0.01	20.14	1.97	-0.04	6123.1	162.3	4.58	49	0	51	H-AL
**El Salvador**	**SLV**	4.13	1.61	-0.02	4.97	0.05	0.00	3.12	0.03	0.00	12.22	1.69	-0.02	7365.8	22.1	1.5	0	77	23	I
**Equatorial Guinea**	**GNQ**	5.42	1.20	-0.02	8.43	2.11	0.00	5.67	3.03	0.00	19.52	6.33	-0.02	18599.6	18.5	1.5	83	13	5	H
**Eritrea**	**ERI**	0.33	0.00	0.00	7.07	0.11	-0.01	2.59	0.50	0.00	10.00	0.61	-0.01	679.9	1.7	1.5	64	0	36	H-AL
**Estonia**	**EST**	0.05	0.00	0.00	0.51	0.01	0.00	0.24	0.01	0.00	0.80	0.01	0.00	17908.2	23.4	-	28	1	71	AL
**Ethiopia**	**ETH**	32.26	9.00	0.00	114.49	12.98	-0.16	70.35	20.58	0.07	217.11	42.55	-0.09	954.4	25.6	8.13	19	0	81	AL
**Fiji**	**FJI**	2.00	1.00	0.00	35.77	9.80	0.00	4.02	2.59	0.07	41.79	13.39	0.07	4358.6	3.6	4.56	-	-	-	-
**Finland**	**FIN**	0.14	0.00	0.00	2.49	0.02	0.00	1.18	0.00	0.00	3.81	0.02	0.00	33555.9	272.7	-	38	4	59	H-AL
**France**	**FRA**	28.15	5.38	-0.20	120.66	41.29	-0.37	34.16	12.00	-0.02	182.97	58.66	-0.60	33678.5	2856.6	-	12	66	22	I-AL
**Gabon**	**GAB**	24.54	1.38	0.00	34.28	1.37	0.00	20.95	3.42	-0.04	79.77	6.18	-0.04	14317.6	14.5	3.4	36	46	18	H-I
**Gambia**	**GMB**	0.13	0.00	0.00	0.70	0.01	0.00	0.37	0.03	0.00	1.21	0.04	0.00	1438.5	0.8	1.5	41	0	59	H-AL
**Georgia**	**GEO**	1.61	0.23	0.00	1.64	0.02	-0.02	6.01	1.03	-0.04	9.27	1.27	-0.05	4757.2	12.8	1.5	61	1	39	H-AL
**Germany**	**DEU**	1.70	0.00	0.00	3.31	0.01	-0.01	3.27	0.01	0.00	8.28	0.02	-0.01	34212.3	3649.5	-	54	12	33	H-AL
**Ghana**	**GHA**	13.71	5.29	-0.05	19.20	1.09	0.00	18.66	1.54	-0.01	51.57	7.92	-0.06	1550.8	16.7	2.62	20	0	80	AL
**Greece**	**GRC**	5.83	3.91	0.00	2.75	0.08	-0.01	6.34	2.04	0.00	14.92	6.03	0.00	29881.5	355.9	-	27	27	46	Mixed
**Grenada**	**GRD**	1.09	1.00	0.00	3.41	1.00	0.00	0.06	0.00	0.00	4.56	2.00	0.00	10712.3	0.6	1.5	-	-	-	-
**Guatemala**	**GTM**	76.71	55.27	-0.60	43.25	3.11	0.00	20.60	4.82	-0.03	140.56	63.20	-0.64	4839.6	39.0	7.99	0	65	35	I-AL
**Guinea**	**GIN**	14.20	2.19	-0.14	13.63	0.57	0.00	14.17	5.36	-0.01	42.00	8.12	-0.15	991.0	3.8	2.43	16	0	84	AL
**Guinea-Bissau**	**GNB**	0.84	0.00	0.00	1.79	0.03	0.00	1.34	0.35	0.00	3.97	0.38	0.00	1068.3	0.4	1.5	59	0	41	H-AL
**Guyana**	**GUY**	36.63	1.82	-0.01	34.75	0.25	0.00	11.46	0.40	0.00	82.84	2.48	-0.02	6688.0	1.2	3.26	12	55	33	I-AL
**Haiti**	**HTI**	36.47	35.50	-0.84	18.39	2.53	-0.01	2.23	0.50	0.00	57.09	38.53	-0.85	1339.0	7.2	4.56	1	4	95	AL
**Honduras**	**HND**	60.59	49.23	-1.30	42.34	2.67	0.00	13.32	1.31	0.00	116.24	53.22	-1.30	4150.8	13.3	7.27	0	76	24	I
**Hungary**	**HUN**	0.61	0.00	0.00	0.89	0.03	0.00	1.18	0.17	0.00	2.69	0.20	0.00	18566.9	154.7	-	28	25	46	Mixed
**Iceland**	**ISL**	0.00	0.00	0.00	1.17	0.00	0.00	0.89	0.22	0.00	2.06	0.22	0.00	38022.5	16.7	-	25	0	75	AL
**India**	**IND**	191.70	61.83	-0.04	300.56	41.96	-0.53	134.44	49.04	-0.33	626.69	152.83	-0.91	2940.7	1159.2	30.58	47	2	51	H-AL
**Indonesia**	**IDN**	238.08	24.18	-0.60	811.97	94.98	-2.64	427.47	147.49	-3.39	1477.52	266.64	-6.63	4156.7	510.7	54.17	32	1	67	AL

**Iran**	**IRN**	10.17	3.22	-0.08	23.08	0.71	-0.04	34.55	3.13	-0.08	67.80	7.06	-0.20	11172.2	286.1	6.33	65	0	35	H-AL
**Iraq**	**IRQ**	0.53	0.32	0.00	4.32	0.51	-0.02	4.20	1.08	-0.01	9.06	1.91	-0.03	3569.9		1.5	65	0	35	H-AL
**Ireland**	**IRL**	0.02	0.00	0.00	0.99	0.09	-0.01	0.62	0.07	0.00	1.63	0.15	-0.01	39468.4	267.6	-	25	0	75	AL
**Israel**	**ISR**	1.09	1.00	0.00	0.82	0.21	-0.01	2.86	0.93	0.00	4.78	2.14	-0.01	28392.9	202.1	-	24	0	76	AL
**Italy**	**ITA**	23.19	7.86	-0.12	4.72	0.14	-0.02	14.61	2.16	0.00	42.52	10.16	-0.14	29109.5	2303.1	-	3	13	84	AL
**Ivory Coast**	**CIV**	24.13	7.82	-0.17	34.99	4.03	0.00	30.48	5.92	-0.01	89.60	17.77	-0.18	1674.2	23.4	3.25	10	0	90	AL
**Jamaica**	**JAM**	21.00	17.00	-0.13	10.57	5.37	0.01	5.61	3.03	0.00	37.19	25.40	-0.11	8777.0	14.6	4.8	0	67	33	I
**Japan**	**JPN**	45.82	19.00	0.00	35.47	10.62	-0.05	51.66	16.63	-0.08	132.95	46.25	-0.13	32607.9	4910.8	-	8	31	62	I-AL
**Jordan**	**JOR**	0.08	0.00	0.00	1.32	0.14	-0.01	0.80	0.04	0.00	2.21	0.18	-0.01	5620.0	21.2	1.5	26	0	74	AL
**Kazakhstan**	**KAZ**	1.56	0.69	0.00	29.00	2.01	-0.08	32.74	2.10	-0.23	63.30	4.80	-0.31	11693.4	133.4	4.76	54	0	46	H-AL
**Kenya**	**KEN**	27.04	5.90	-0.04	96.32	9.17	-0.07	56.66	11.16	-0.04	180.02	26.23	-0.15	1729.6	30.4	8.95	34	1	65	H-AL
**Kiribati**	**KIR**	0.00	0.00	0.00	6.04	2.86	0.00	0.84	0.11	0.01	6.89	2.97	0.00	6048.6	0.1	1.69	25	0	75	AL
**Kuwait**	**KWT**	0.00	0.00	0.00	0.24	0.06	0.00	0.07	0.01	0.00	0.31	0.07	0.00	38304.0	148.0	-	55	0	45	H-AL
**Kyrgyzstan**	**KGZ**	1.23	0.00	0.00	4.90	0.09	-0.01	6.02	0.16	-0.02	12.16	0.24	-0.02	2253.5	5.1	1.5	27	0	73	AL
**Laos**	**LAO**	20.18	1.50	-0.01	45.55	1.55	-0.03	32.52	9.73	-0.20	98.25	12.79	-0.23	2266.0	5.5	6.11	67	0	33	H
**Latvia**	**LVA**	0.14	0.00	0.00	0.64	0.00	0.00	0.37	0.00	0.00	1.14	0.00	0.00	14254.6	33.8	-	37	2	60	H-AL
**Lebanon**	**LBN**	0.29	0.00	0.00	0.36	0.21	-0.01	0.25	0.01	0.00	0.90	0.22	-0.01	14225.9	29.3	1.5	3	0	97	AL
**Lesotho**	**LSO**	3.09	0.00	0.00	3.23	0.19	0.00	2.42	0.06	0.00	8.75	0.25	0.00	1218.3	1.6	1.5	67	4	29	H
**Liberia**	**LBR**	14.70	1.70	-0.13	16.31	3.94	0.00	15.94	5.08	0.00	46.95	10.72	-0.14	424.3	0.8	2.42	4	0	96	AL
**Libya**	**LBY**	0.41	0.00	0.00	5.15	0.25	-0.02	10.13	0.87	-0.01	15.69	1.12	-0.03	14328.1	93.2	-	74	0	26	H
**Liechtenstein**	**LIE**	0.00	0.00	0.00	0.00	0.00	0.00	0.00	0.00	0.00	0.01	0.00	0.00	-	4.2	1.5	11	28	61	I-AL
**Lithuania**	**LTU**	0.17	0.00	0.00	0.71	0.01	0.00	0.38	0.00	0.00	1.26	0.01	0.00	16542.3	47.3	-	49	3	49	H-AL
**Luxembourg**	**LUX**	0.02	0.00	0.00	0.02	0.00	0.00	0.03	0.00	0.00	0.06	0.00	0.00	78395.2	53.7	-	56	12	32	H-AL
**Madagascar**	**MDG**	238.07	67.00	-0.17	84.63	29.51	-0.11	184.88	58.06	0.00	507.58	154.57	-0.27	932.4	9.5	26.06	40	20	39	H-AL
**Malawi**	**MWI**	10.52	4.84	0.00	15.80	2.04	-0.04	8.86	0.07	0.00	35.18	6.95	-0.04	884.7	4.3	4.39	8	1	91	AL
**Malaysia**	**MYS**	131.60	41.73	-0.89	88.98	8.28	-0.90	90.43	23.91	-1.03	311.01	73.92	-2.82	13769.3	221.8	14.66	13	0	87	AL
**Maldives**	**MDV**	0.00	0.00	0.00	0.72	0.00	0.00	0.23	0.02	0.00	0.94	0.02	0.00	4894.0	1.3	2.54	25	0	75	AL
**Mali**	**MLI**	3.21	0.00	0.00	22.94	0.34	-0.02	12.86	0.54	-0.03	39.01	0.88	-0.04	1173.0	8.7	1.96	63	0	37	H-AL

**Malta**	**MLT**	0.00	0.00	0.00	0.08	0.01	0.00	0.02	0.00	0.00	0.10	0.01	0.00	23584.0	7.4	-	-	-	-	-
**Marshall Islands**	**MHL**	0.00	0.00	0.00	1.96	0.65	0.00	0.49	0.06	0.00	2.45	0.72	0.00	-	0.2	2.02	25	0	75	AL
**Mauritania**	**MRT**	0.23	0.00	0.00	7.31	0.07	-0.01	6.90	0.51	-0.02	14.45	0.58	-0.03	2037.4	2.9	2.05	66	0	34	H
**Mauritius**	**MUS**	0.00	0.00	0.00	10.29	9.51	0.40	2.70	2.42	0.00	12.98	11.92	0.40	12526.7	9.3	5.19	44	11	45	H-AL
**Mexico**	**MEX**	292.93	188.40	-2.68	333.68	31.55	-0.07	251.07	88.40	-0.46	877.68	308.35	-3.21	13628.1	1088.1	52.75	11	57	32	I-AL
**Micronesia**	**FSM**	0.00	0.00	0.00	18.66	4.14	0.00	5.06	3.28	0.01	23.72	7.42	0.01	-	0.3	3.49	25	0	75	AL
**Moldova**	**MDA**	0.09	0.00	0.00	0.37	0.01	0.00	0.40	0.02	0.00	0.86	0.03	0.00	2842.6	6.0	1.5	55	9	36	H-AL
**Monaco**	**MCO**	0.00	0.00	0.00	0.00	0.00	0.00	0.00	0.00	0.00	0.00	0.00	0.00	-		-	75	0	25	H
**Mongolia**	**MNG**	0.26	0.00	0.00	18.69	2.31	-0.06	23.02	3.78	-0.09	41.97	6.09	-0.15	3481.1	5.3	4.33	64	2	33	H-AL
**Montenegro**	**MNE**	0.27	0.13	-0.01	0.19	0.00	0.00	1.97	0.30	0.00	2.44	0.43	-0.01	10393.2	4.9	1.5	49	49	1	H-I
**Morocco**	**MAR**	6.00	1.50	-0.04	10.50	0.49	-0.02	15.05	4.18	-0.09	31.55	6.17	-0.15	4604.2	88.9	4.9	14	23	63	I-AL
**Mozambique**	**MOZ**	17.83	0.95	0.00	51.17	2.63	-0.05	24.89	1.62	0.00	93.89	5.20	-0.06	933.8	9.8	7	27	5	68	AL
**Myanmar**	**MMR**	28.09	0.06	-0.01	118.73	7.23	-0.11	47.35	9.60	-0.20	194.17	16.89	-0.33	1197.3		6.72	47	0	53	H-AL
**Namibia**	**NAM**	8.99	0.72	0.00	55.41	2.85	-0.06	28.36	1.63	0.00	92.75	5.21	-0.06	6614.0	8.8	6.28	83	0	17	H
**Nauru**	**NRU**	0.00	0.00	0.00	1.24	1.05	0.00	0.08	0.01	0.00	1.32	1.06	0.00	-		1.5	-	-	-	-
**Nepal**	**NPL**	18.65	2.49	0.00	32.08	2.03	-0.06	13.65	3.87	0.03	64.38	8.38	-0.02	1205.3	12.6	2.67	11	0	89	AL
**Netherlands**	**NLD**	0.12	0.00	0.00	0.80	0.05	0.00	0.39	0.03	0.00	1.32	0.09	0.00	39937.9	871.0	-	20	30	50	I-AL
**New Zealand**	**NZL**	4.00	4.00	-0.18	83.63	49.08	-0.16	10.00	4.93	-0.14	97.63	58.01	-0.49	26707.8	129.9	-	0	92	8	I
**Nicaragua**	**NIC**	19.91	4.99	-0.13	41.35	1.66	0.00	14.87	0.30	0.00	76.13	6.95	-0.13	2626.9	6.6	3.94	0	67	33	I
**Niger**	**NER**	0.61	0.00	0.00	13.59	0.22	-0.02	8.18	1.64	-0.11	22.39	1.86	-0.13	719.1	5.4	1.5	61	0	39	H-AL
**Nigeria**	**NGA**	11.05	2.15	-0.01	60.57	2.81	-0.01	36.04	5.84	-0.09	107.66	10.79	-0.11	2249.0	207.1	5.64	12	0	88	AL
**Niue**	**NIU**	0.00	0.00	0.00	0.51	0.14	0.00	0.08	0.01	0.00	0.59	0.15	0.00	-		1.5	25	0	75	AL
**North Korea**	**PRK**	2.46	0.65	-0.03	3.30	0.31	0.00	2.87	0.41	-0.01	8.64	1.37	-0.03	-		1.5	25	1	75	AL
**Norway**	**NOR**	0.09	0.00	0.00	5.59	0.32	-0.01	4.36	0.54	-0.01	10.04	0.86	-0.02	52561.3	451.8	-	33	17	50	H-AL
**Oman**	**OMN**	0.59	0.00	0.00	3.82	0.24	0.00	5.22	1.86	0.00	9.63	2.10	-0.01	25109.8	41.6	-	52	0	48	H-AL
**Pakistan**	**PAK**	3.53	0.00	0.00	25.77	1.57	-0.04	13.06	2.18	-0.01	42.36	3.75	-0.05	2661.0	164.5	4.92	68	0	32	H
**Palau**	**PLW**	1.00	0.00	0.00	12.20	0.32	0.00	1.41	0.24	0.00	14.61	0.56	0.00	-	0.2	1.92	25	0	75	AL
**Panama**	**PAN**	79.53	31.77	-0.99	67.73	8.14	0.00	26.68	5.27	0.00	173.93	45.18	-0.99	11788.1	23.1	11.29	0	66	34	I-AL

**Papua New Guinea**	**PNG**	227.68	9.76	-0.17	297.72	25.17	-0.48	153.98	32.46	-1.00	679.37	67.39	-1.65	2166.7	8.2	13.32	32	2	67	AL
**Paraguay**	**PRY**	9.63	0.00	-0.01	38.69	1.92	-0.01	17.07	1.66	-0.01	65.39	3.58	-0.03	4533.1	16.0	2.95	81	0	19	H
**Peru**	**PER**	302.12	80.86	-1.15	471.71	54.96	-0.20	140.23	28.85	-0.09	914.05	164.68	-1.43	8638.4	129.1	26.25	28	52	19	H-I
**Philippines**	**PHL**	81.11	48.00	-0.14	225.46	62.55	-0.17	123.22	27.64	-0.34	429.78	138.19	-0.65	3520.6	166.9	25.96	54	1	46	H-AL
**Poland**	**POL**	1.14	0.00	0.00	3.06	0.04	0.00	2.99	0.59	0.05	7.19	0.63	0.04	18072.4	527.9	-	45	16	38	H-AL
**Portugal**	**PRT**	2.79	0.34	-0.04	8.17	2.96	0.04	4.95	1.83	-0.01	15.91	5.13	-0.01	21858.7	243.5	-	15	37	48	I-AL
**Qatar**	**QAT**	0.00	0.00	0.00	0.19	0.05	0.00	0.05	0.01	0.00	0.24	0.06	0.00	83840.7	71.0	-	70	0	30	H
**Republique du Congo**	**COG**	11.51	0.00	0.00	31.82	0.20	0.00	25.86	1.50	-0.04	69.19	1.70	-0.04	4145.5	10.7	3.28	39	43	18	H-I
**Romania**	**ROU**	1.41	0.00	0.00	2.18	0.04	0.00	4.51	0.27	0.00	8.09	0.30	0.00	11917.3	200.1	-	45	15	40	H-AL
**Russia**	**RUS**	8.84	0.00	0.00	155.38	13.99	-0.40	103.66	8.68	-0.17	267.88	22.68	-0.57	14920.0	1679.5	24.37	54	6	40	H-AL
**Rwanda**	**RWA**	7.79	3.20	0.00	11.05	1.13	-0.01	8.42	3.77	0.00	27.26	8.10	-0.01	1149.9	4.5	1.5	13	1	85	AL
**Saint Kitts and Nevis**	**KNA**	0.05	0.00	0.00	0.48	0.00	0.00	0.11	0.00	0.00	0.64	0.01	0.00	13429.2	0.5	1.5	25	0	75	AL
**Saint Lucia**	**LCA**	0.11	0.00	-0.01	6.69	3.34	-0.01	0.29	0.00	0.00	7.10	3.34	-0.01	10177.7	1.0	1.87	18	36	46	I-AL
**Saint Vincent and the Grenadines**	**VCT**	1.08	1.00	0.00	2.69	2.01	0.00	0.18	0.00	0.00	3.95	3.01	0.00	9976.5	0.6	1.5	-	-	-	-
**Samoa**	**WSM**	0.00	0.00	0.00	13.04	5.07	-0.10	0.22	0.00	0.01	13.26	5.08	-0.09	5782.1	0.5	2.43	0	40	60	I-AL
**San Marino**	**SMR**	0.00	0.00	0.00	0.00	0.00	0.00	0.00	0.00	0.00	0.00	0.00	0.00	-	1.7	-	-	-	-	-
**Sao Tome and Principe**	**STP**	7.00	3.00	-0.08	24.07	9.00	0.00	5.07	3.01	0.00	36.14	15.01	-0.08	1814.1	0.2	2.77	0	0	100	AL
**Saudi Arabia**	**SAU**	1.36	0.00	0.00	18.12	2.22	-0.02	10.60	1.49	-0.03	30.08	3.71	-0.05	23221.4	468.8	-	64	0	36	H-AL
**Senegal**	**SEN**	1.15	0.00	0.00	8.09	0.19	-0.01	6.49	0.31	-0.08	15.73	0.50	-0.08	1743.1	13.3	1.8	66	0	34	H-AL
**Serbia**	**SRB**	0.73	0.00	0.00	1.03	0.00	0.00	1.46	0.14	0.00	3.22	0.15	0.00	10635.4	50.1	1.5	24	22	55	H-AL
**Seychelles**	**SYC**	11.00	6.00	0.00	12.88	7.01	0.10	2.45	2.07	0.00	26.33	15.07	0.10	20411.0	0.8	4.9	27	13	61	H-AL
**Sierra Leone**	**SLE**	7.89	1.18	-0.04	8.54	0.51	0.00	6.33	1.45	0.00	22.77	3.14	-0.05	759.2	2.0	1.5	11	0	89	AL
**Singapore**	**SGP**	1.06	0.00	0.00	0.08	0.01	0.00	1.03	0.01	0.00	2.18	0.02	0.00	50522.7	181.9	-	20	0	80	AL
**Slovakia**	**SVK**	0.45	0.00	0.00	0.46	0.00	0.00	1.02	0.08	0.00	1.93	0.08	0.00	21244.9	98.5	-	38	21	41	H-AL
**Slovenia**	**SVN**	0.38	0.19	0.00	0.22	0.00	0.00	0.72	0.01	0.00	1.32	0.20	0.00	27654.3	54.6	-	0	34	66	I-AL

**Solomon Islands**	**SLB**	9.53	1.24	0.00	68.67	12.21	-0.02	25.93	14.66	-0.29	104.13	28.10	-0.31	2818.7	0.6	3.6	14	0	86	AL
**Somalia**	**SOM**	7.98	0.00	0.00	48.60	3.25	-0.01	27.03	2.60	-0.08	83.61	5.84	-0.09	-		-	67	0	33	H
**South Africa**	**ZAF**	68.56	19.28	-0.25	137.44	13.13	-0.14	83.58	12.74	0.00	289.58	45.15	-0.40	10243.6	276.4	21.68	3	19	78	AL
**South Korea**	**KOR**	7.05	1.35	-0.02	4.86	1.13	0.00	2.49	0.30	-0.01	14.39	2.78	-0.03	27977.9	929.1	-	37	0	63	H-AL
**Spain**	**ESP**	14.85	4.65	-0.05	17.36	3.40	-0.04	17.18	6.03	-0.01	49.39	14.08	-0.10	29689.3	1604.2	-	19	33	47	I-AL
**Sri Lanka**	**LKA**	69.42	52.61	0.00	32.48	8.23	0.00	22.90	17.22	-0.01	124.80	78.06	-0.01	4768.5	40.6	7.84	73	0	27	H
**Sudan**	**SDN**	3.37	0.00	0.00	70.81	1.76	-0.11	36.60	1.77	-0.10	110.77	3.53	-0.21	2380.3	55.9	3.68	60	0	40	H-AL
**Suriname**	**SUR**	15.05	0.54	0.07	21.56	0.15	0.00	8.24	0.19	0.00	44.84	0.88	0.07	8642.0	3.0	3	10	57	33	I-AL
**Swaziland**	**SWZ**	1.13	0.00	0.00	2.41	0.08	0.00	0.94	0.09	0.00	4.48	0.17	0.00	5708.6	2.8	1.5	83	0	16	H
**Sweden**	**SWE**	0.21	0.00	0.00	3.14	0.03	0.00	1.58	0.00	0.00	4.93	0.03	0.00	35964.8	479.0	-	37	2	60	H-AL
**Switzerland**	**CHE**	0.32	0.00	0.00	0.50	0.00	0.00	1.53	0.00	0.00	2.34	0.01	0.00	43007.5	491.9	-	44	17	39	H-AL
**Syria**	**SYR**	0.33	0.00	0.00	1.43	0.07	0.00	4.07	0.82	0.00	5.83	0.89	-0.01	4887.4	55.2	1.5	56	0	44	H-AL
**Tajikistan**	**TJK**	1.00	0.00	0.00	3.58	0.08	0.00	3.74	0.22	0.00	8.32	0.30	0.00	2103.6	5.1	1.5	64	0	36	H-AL
**Tanzania**	**TZA**	102.68	48.10	-0.17	139.66	22.56	-0.12	68.88	23.54	0.02	311.22	94.19	-0.27	1415.9	20.5	13.95	48	11	41	H-AL
**Thailand**	**THA**	46.77	2.43	-0.07	52.31	2.67	-0.13	40.15	6.22	-0.29	139.24	11.32	-0.49	8059.8	272.4	9.05	44	0	56	H-AL
**The Bahamas**	**BHS**	1.12	0.00	0.00	10.29	1.14	0.02	2.47	1.18	0.00	13.87	2.32	0.02	26473.7	7.2	4.26	-	-	-	-
**The Former Yugoslav Republic of Macedonia**	**MKD**	0.27	0.00	0.00	0.38	0.00	0.00	1.25	0.14	0.00	1.90	0.15	0.00	9170.7	9.5	1.5	27	16	57	H-AL
**Togo**	**TGO**	3.48	1.65	0.00	3.41	0.01	0.00	2.88	0.13	0.00	9.78	1.79	0.00	826.4	2.9	1.5	54	0	46	H-AL
**Tonga**	**TON**	0.00	0.00	0.00	3.09	1.31	0.05	0.17	0.02	0.00	3.26	1.34	0.05	7060.6	0.3	1.59	25	0	75	AL
**Trinidad and Tobago**	**TTO**	8.38	7.28	-0.13	1.82	1.01	0.00	3.09	0.02	0.00	13.28	8.31	-0.12	19817.9	24.1	2.74	0	100	0	I
**Tunisia**	**TUN**	0.57	0.00	0.00	3.62	0.21	-0.01	4.85	0.21	-0.01	9.04	0.42	-0.02	8254.4	40.3	1.5	52	0	48	H-AL
**Turkey**	**TUR**	16.67	10.23	-0.09	11.67	0.33	-0.06	23.06	1.80	-0.02	51.40	12.36	-0.17	12476.4	734.9	5.65	42	27	31	Mixed
**Turkmenistan**	**TKM**	0.44	0.00	0.00	7.18	0.35	-0.01	7.22	0.19	-0.02	14.84	0.54	-0.03	5971.2	15.3	1.7	49	0	51	H-AL
**Tuvalu**	**TUV**	0.00	0.00	0.00	0.79	0.27	0.00	0.19	0.02	0.00	0.97	0.29	0.00	-		1.5	-	-	-	-
**Uganda**	**UGA**	9.01	0.88	0.00	36.38	1.74	-0.01	22.81	6.16	-0.01	68.20	8.78	-0.02	1195.5	14.3	3.83	44	3	53	H-AL

**Ukraine**	**UKR**	1.58	0.00	0.00	6.47	0.15	-0.01	8.43	2.12	0.03	16.47	2.27	0.02	6339.1	180.4	1.5	47	9	43	H-AL
**United Arab Emirates**	**ARE**	0.07	0.00	0.00	0.81	0.10	0.00	0.60	0.27	0.00	1.48	0.37	0.00	36536.8	198.7	-	49	0	51	H-AL
**United Kingdom**	**GBR**	1.44	1.05	0.00	40.79	20.05	-0.20	10.23	0.51	0.01	52.46	21.61	-0.19	34619.0	2674.1	-	1	53	46	I-AL
**United States**	**USA**	260.08	68.30	-1.51	292.15	63.26	-1.55	263.06	27.37	0.01	815.28	158.93	-3.05	46380.9	14093.3	-	2	70	28	I
**Uruguay**	**URY**	8.77	2.83	-0.06	14.51	1.20	0.00	10.23	2.28	0.00	33.52	6.32	-0.06	13163.0	32.2	1.99	5	0	95	AL
**Uzbekistan**	**UBZ**	0.13	0.00	0.00	6.08	0.42	-0.02	6.62	0.49	-0.04	12.83	0.91	-0.06	2806.7	27.9	1.65	37	0	63	H-AL
**Vanuatu**	**VUT**	0.00	0.00	0.00	16.85	5.97	0.00	3.51	3.10	0.00	20.36	9.06	0.00	4737.5	0.6	2.55	25	0	75	AL
**Vatican City**	**VAT**	0.00	0.00	0.00	0.00	0.00	0.00	0.00	0.00	0.00	0.00	0.00	0.00	-		-	68	0	32	H
**Venezuela**	**VEN**	203.10	69.89	-1.26	228.18	17.86	-0.09	83.68	16.11	-0.12	514.96	103.87	-1.46	12201.0	314.2	14.49	2	65	33	I-AL
**Viet Nam**	**VNM**	73.09	9.91	-0.02	65.22	7.95	-0.02	42.27	11.28	-0.28	180.57	29.14	-0.32	2942.1	90.6	12.12	54	0	46	H-AL
**Yemen**	**YEM**	3.07	1.00	0.00	22.08	3.93	0.00	5.49	0.16	0.00	30.64	5.09	-0.01	2457.8	26.6	4.27	70	0	30	H
**Zambia**	**ZMB**	19.26	0.04	0.00	70.47	3.64	-0.02	31.49	2.38	-0.01	121.22	6.06	-0.03	1541.7	14.3	4.26	55	6	39	H-AL
**Zimbabwe**	**ZWE**	11.76	5.18	-0.08	31.36	1.00	-0.01	17.88	0.99	0.00	61.00	7.17	-0.09	354.9	3.4	1.72	63	4	33	H-AL
